# Hexahexyloxycalix[6]arene, a Conformationally Adaptive Host for the Complexation of Linear and Branched Alkylammonium Guests

**DOI:** 10.3390/molecules28124749

**Published:** 2023-06-13

**Authors:** Veronica Iuliano, Carmen Talotta, Paolo Della Sala, Margherita De Rosa, Annunziata Soriente, Placido Neri, Carmine Gaeta

**Affiliations:** Dipartimento di Chimica e Biologia “A. Zambelli”, Università di Salerno, Via Giovanni Paolo II 132, I-84084 Salerno, Italy; viuliano@unisa.it (V.I.); pdellasala@unisa.it (P.D.S.); maderosa@unisa.it (M.D.R.); titti@unisa.it (A.S.); neri@unisa.it (P.N.)

**Keywords:** conformation, calixarene, molecular recognition, alkylammonium guests

## Abstract

Hexahexyloxycalix[6]arene **2b** leads to the *endo*-cavity complexation of linear and branched alkylammonium guests showing a conformational adaptive behavior in CDCl_3_ solution. Linear *n*-pentylammonium guest **6a^+^** induces the cone conformation of **2b** at the expense of the 1,2,3-alternate, which is the most abundant conformer of **2b** in the absence of a guest. In a different way, branched alkylammonium guests, such as *tert*-butylammonium **6b^+^** and isopropylammonium **6c^+^**, select the 1,2,3-alternate as the favored **2b** conformation (**6b^+^**/**6c^+^**⊂**2b*^1,2,3-alt^***), but other complexes in which **2b** adopts different conformations, namely, **6b^+^**/**6c^+^**⊂**2b*^cone^***, **6b^+^**/**6c^+^**⊂**2b*^paco^***, and **6b^+^**/**6c^+^**⊂**2b*^1,2-alt^***, have also been revealed. Binding constant values determined via NMR experiments indicated that the 1,2,3-alternate was the best-fitting **2b** conformation for the complexation of branched alkylammonium guests, followed by cone > paco > 1,2-alt. Our NCI and NBO calculations suggest that the H-bonding interactions (^+^N–H···O) between the ammonium group of the guest and the oxygen atoms of calixarene **2b** are the main determinants of the stability order of the four complexes. These interactions are weakened by increasing the guest steric encumbrance, thus leading to a lower binding affinity. Two stabilizing H-bonds are possible with the 1,2,3-alt- and cone-**2b** conformations, whereas only one H-bond is possible with the other paco- and 1,2-alt-**2b** stereoisomers.

## 1. Introduction

Molecular recognition [1] is a fundamental process in living systems, and due to our understanding of the secondary interactions that stabilize the ligand@protein complexes, it has been possible to design novel biomimetic guest@host supramolecular systems. A natural process such as protein–substrate binding often involves conformational changes, which can occur prior to the binding event (‘*conformational selection model*’ [2,3]) or during the binding event (‘*induced-fit model*’ [4,5]). According to the *conformational selection model*, the protein conformational changes may take place prior to ligand binding, and then the stabilization of a specific protein structure is caused by its complexation with the substrate. In contrast, in the *induced-fit binding model*, the conformational change takes place upon substrate binding [6].

The design of artificial ammonium receptors is an exciting topic of research in supramolecular chemistry, which takes inspiration from natural systems [7,8]. Thus, the recognition of ammonium guests by macrocycles such as calixarenes [9], pillararenes [10,11], prismarenes [12,13,14], cucurbiturils [15], naphthotubes [16], oxatubarene [17], cycloparaphenylenes [18], and saucerarenes [19], has received substantial attention in recent years. In particular, very recently, Jiang [17] reported a new class of macrocycles named oxatub[4]arenes, which showed biomimetic conformational adaptation behavior [20,21]. In fact, oxatubarenes can take on four interconvertible conformations through the flipping of naphthalene rings. Jiang showed that according to the “conformational selection model”, a specific ammonium guest can select the best-fitting oxatubarene conformer, altering the initial equilibrium distribution of the conformers. Calix[2]naphth[2]arene [22] macrocycle, which we reported in 2020, is composed of two phenol and two naphthalene rings and can adopt five potential conformations, but the 1,2-alternate conformation is the only one that achieves the best binding when alkali metal cations are present.

Additionally, calix[5]arene macrocycles can show conformational response to ammonium guests [23]. In fact, very recently, we showed that the cone and partial cone are the best-fitting conformers of a calix[5]arene for secondary ammonium cations.

**Chart 1:**
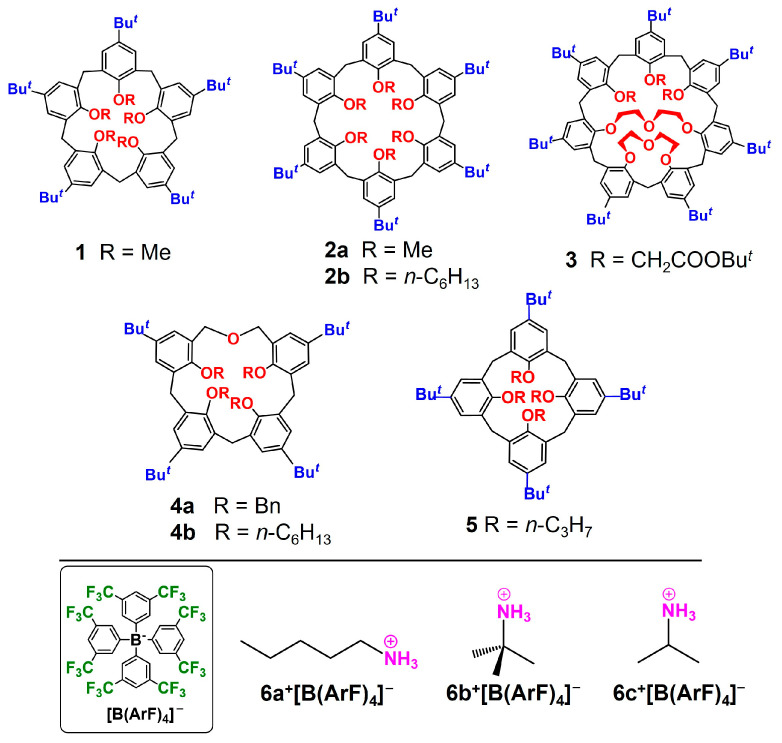
Structures of calix[*n*]arene derivatives **1–5** and alkylammonium **6a**−**d**^+^[B(ArF)_4_]^−^ salts.

In 2010 [24], our group showed that conformationally mobile hexamethoxycalix[6]arene macrocycle **2a** forms pseudorotaxane complexes via threading with dialkylammonium cations coupled with the weakly coordinating barfate anion (BArF^−^ = tetrakis [3,5-bis(trifluoromethyl)phenyl]borate) (Chart 1). The *endo*-cavity complexation of linear and branched alkylammonium cations, as barfate salts, was also observed with calix[5]arene **1** [23], doubly bridged calix[7]arene **3** [9], dihomooxacalix[4]arene **4** [25,26], and calix[4]arene **5** [27].

**Figure 1:**
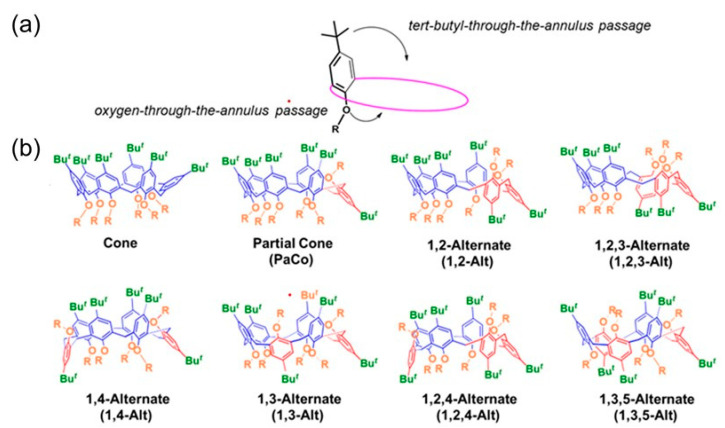
(**a**) Possible pathways for conformational interconversion in *p-tert*-butylcalix[6]arenes. (**b**) The eight basic conformations of calix[6]arene derivatives.

The calix[6]arene macrocycle can adopt eight distinct conformations (Figure 1), including cone, partial cone, 1,2-alternate, 1,3-alternate, 1,4-alternate, 1,2,3-alternate, 1,2,4-alternate, and 1,3,5-alternate [28,29,30], and conformational interconversion between them occurs by means of “*oxygen-through-the-annulus*” or/and “*tert-butyl-through-the-annulus*” passage.

**Figure 2:**
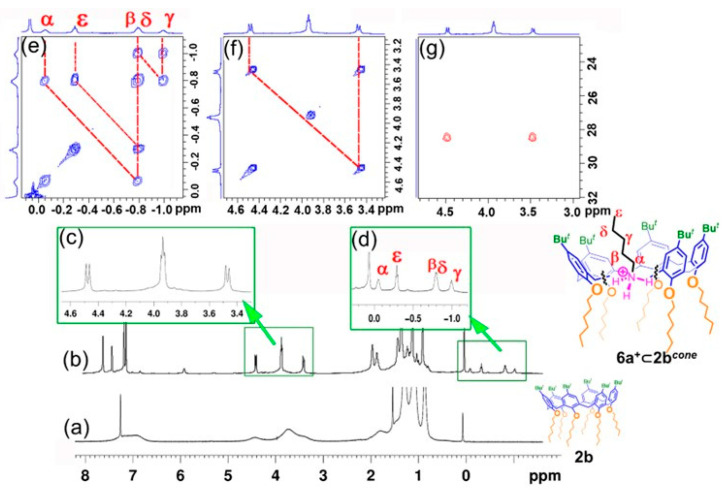
1D and 2D NMR spectra (CDCl_3_, 600 MHz, 298 K) for the complexation of **6a^+^** by **2b**. (**a**) ^1^H NMR spectrum of **2b**; (**b**) ^1^H NMR spectrum of an equimolar solution of **2b** and **6a^+^**[B(ArF)_4_]^−^ (3 mM); (**c**,**d**) enlargements of two different portions of (**b**); (**e**,**f**) portions of the COSY-45; and (**g**) HSQC spectrum (CDCl_3_, 600 MHz, 298 K) of the solution in (**b**).

As previously reported by us [30], the ^1^H NMR spectrum of hexahexyloxycalix[6]arene **2b** in CDCl_3_ at 298 K (Figure 2a) shows broad signals indicative of a slow conformational mobility of the macrocycle with respect to the NMR time scale. We showed that after lowering the temperature to 233 K, the ^1^H NMR resonances of **2b** decoalesced to form sharp signals compatible with the presence of 1,2,3-alternate (favored) and cone conformations of **2b**. Based on these considerations, hexahexyloxycalix[6]arene **2b** is the ideal candidate for studying the conformational response of the calix[6]arene skeleton to the presence of ammonium guests. The question to address now is specifically whether linear and branched alkylammonium cations **6a**–**c^+^** can form complexes with **2b,** as well as the possibility of complexation-induced selection of the 1,2,3-alternate/cone or alternative conformations of hexahexyloxycalix[6]arene **2b**.

Consequently, prompted by these considerations, we decided to investigate the molecular recognition properties of **2b** toward linear and branched alkylammonium ions **6a**–**c^+^** as barfate salts [B(ArF)_4_]^–^ (Chart 1).

## 2. Results and Discussion

### 2.1. Binding Ability of ***2b*** toward n-Pentylammonium Guest ***6a***^+^[B(ArF)_4_]^–^

**Scheme 1:**
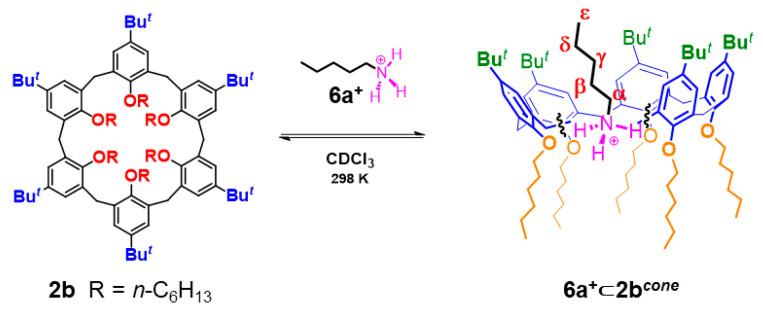
Formation of the **6a^+^**⊂**2b*^cone^*** complex.

The complexation ability of **2b** toward **6a^+^**[B(ArF)_4_]^–^ (Chart 1) was investigated at 298 K via 1D and 2D NMR experiments. The ^1^H NMR spectrum of an equimolar (3 mM) solution of **2b** and **6a^+^**[B(ArF)_4_]^–^ in CDCl_3_ at 298 K showed typical features [24] of the presence of the *endo*-cavity **6a^+^**⊂**2b** complex (Scheme 1). In particular, the formation of the complex was ascertained based on the appearance of a new set of slowly exchanging signals in the up-field negative region of the spectrum (Figure 2) attributable to the *n*-pentyl chain of **6a^+^** shielded inside the cavity of the calix[6]arene macrocycle.

The NMR signals of **6a^+^** complexed inside the cavity of **2b** were assigned via a COSY-45 experiment (Figure 2e). As a result, the NH_3_^+^ signal at 5.99 ppm correlated with the *α*-protons at –0.06 ppm, which coupled with the *β*-methylene group at –0.81 ppm. This, in turn, showed a cross peak with the *γ*-protons at –0.99 ppm. The *γ*-protons were coupled with the *δ*-methylene group at –0.81 ppm, which was correlated with the *ε*-methyl group at –0.28 ppm. The COSY-45 spectrum of the **6a^+^**⊂**2b** complex revealed the presence of an AX system (Figure 2f) at 3.47/4.48 ppm (Δ*δ* = 0.99 ppm), which correlated in the HSQC spectrum with a ^13^C resonance at 28.4 ppm, attributable to the ArCH_2_Ar groups. In agreement with Gutsche’s “^1^H NMR” rule [28,30] and the “^13^C NMR single rule” of de Mendoza [29,30], these results are only compatible with the formation of the *endo*-cavity **6a^+^**⊂**2b*^cone^*** complex, in which the calix[6]arene adopts a cone conformation. In conclusion, the pentylammonium cation **6a^+^** selected the cone as the best-fitting **2b** conformation at the expense of the 1,2,3-alternate, which was the most abundant species in the initial conformational equilibria of **2b** [30].

The calculation of the binding constant for the formation of the **6a^+^**⊂**2b*^cone^*** complex through direct peak integration was not possible, as the ^1^H NMR signals (Figure 2b) of the free **2b** and guest **6a^+^** were not detected in the ^1^H NMR spectrum of their 1:1 mixture, shown in Figure 2b. Consequently, a binding constant calculation was performed by means of a competition experiment in which 1 equiv of pentylammonium **6a^+^** was mixed with a 1:1 mixture of **2b** and *n*-butylammonium (in CDCl_3_) as barfate salt. Previously, we reported on the formation of the ***n*-BuNH_3_^+^⊂**2b***^cone^*** complex in CDCl_3_ with a *K*_ass_ value of 8.3 ± 0.1 × 10^6^ M^–1^ [12,13,24,31]. After the mixing of **2b**, ***n*-BuNH_3_^+^**, and **6a^+^** (1/1/1 molar ratio), the ***n*-BuNH_3_^+^⊂**2b***^cone^*** complex was preferentially formed over the **6a^+^**⊂**2b*^cone^*** one in a 1.6:1.0 ratio. Thus, from these data, a binding constant of 5.2 ± 0.1 × 10^6^ M^–1^ was calculated for the formation of the **6a^+^**⊂**2b*^cone^*** complex in CDCl_3_ at 298 K (see the Supplementary Materials for further details).

**Figure 3:**
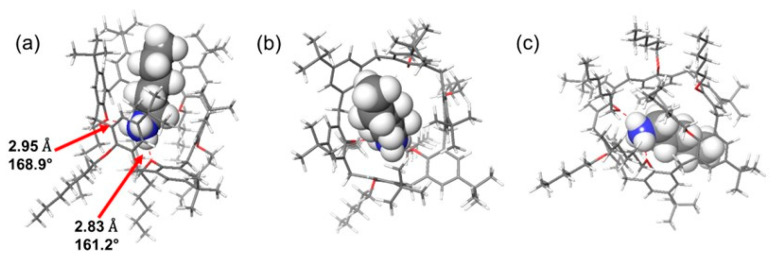
(**a**–**c**) Different views of the DFT-optimized structure of the **6a^+^**⊂**2b*^cone^*** complex. (**a**) Marked in red, ^+^N–H···O, H-bonding mean distance and angle.

The DFT-optimized structure of the **6a^+^**⊂**2b*^cone^*** complex (Figure 3), calculated on the B3LYP/6-31G(d,p) theoretical level, revealed that the N^+^ atom of **6a^+^** sits 0.30 Å above the mean plane of the ethereal oxygen atoms of **2b**. H-bonding interactions were detected between the ^+^NH_3_−ammonium group of **6a^+^** and the oxygen atoms of **2b**, with a ^+^N···O***^2b^*** average distance of 2.83 Å and an average ^+^N-H··· O***^2b^*** angle of 165.1°. In addition, CH···π interactions were detected between the aromatic rings of **2b** and the pentyl chain of **6a^+^** confined inside the cavity of **2b** (Figure 2a–c), with an average C−H···π*^centroid^* distance of 2.99 Å. A second-order perturbation theory SOPT analysis [32] of the Fock matrix in the NBO [33] basis and NCI (non-covalent interactions) studies were conducted in order to rationalize the energy contribution of secondary interactions.

Interestingly, the SOPT analysis conducted on **6a^+^**⊂**2b*^cone^*** indicated that a network of ^+^N–H···O***^2b^*** hydrogen bonding and C-H···π interactions stabilized the complex. We identified two lone-pair (LP) interactions between the oxygen atoms of **2b** and the N–H antibonding orbitals (i.e., H-bonding interactions, Figure 3) of **6a^+^** in the **6a^+^**⊂**2b** complex, which provided a 78% energetic contribution to the total stabilization energy of the complex.

### 2.2. Binding Ability of ***2b*** toward Tert-Butylammonium Guest ***6b***^+^[B(ArF)_4_]^–^

**Scheme 2:**
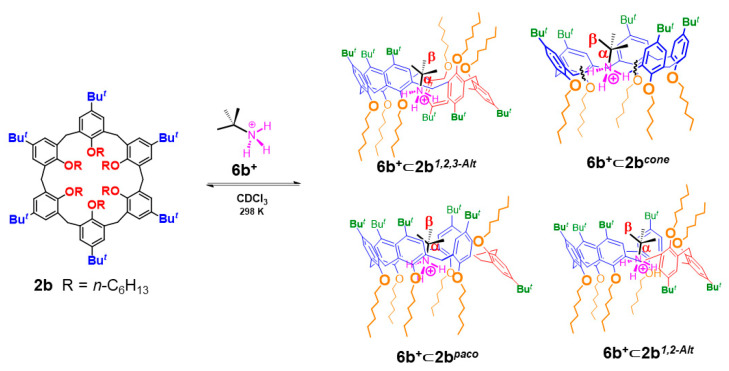
*Endo*-cavity complexation of *tert*-butylammonium **6b^+^** as barfate salt [B(ArF)_4_]^–^ by **2b**: formation of **6b^+^**⊂**2b*^1,2,3-Alt^***, **6b^+^**⊂**2b*^cone^***, **6b^+^**⊂**2b*^paco^***, and **6b^+^**⊂**2b*^1,2-Alt^*** complexes.

**Figure 4:**
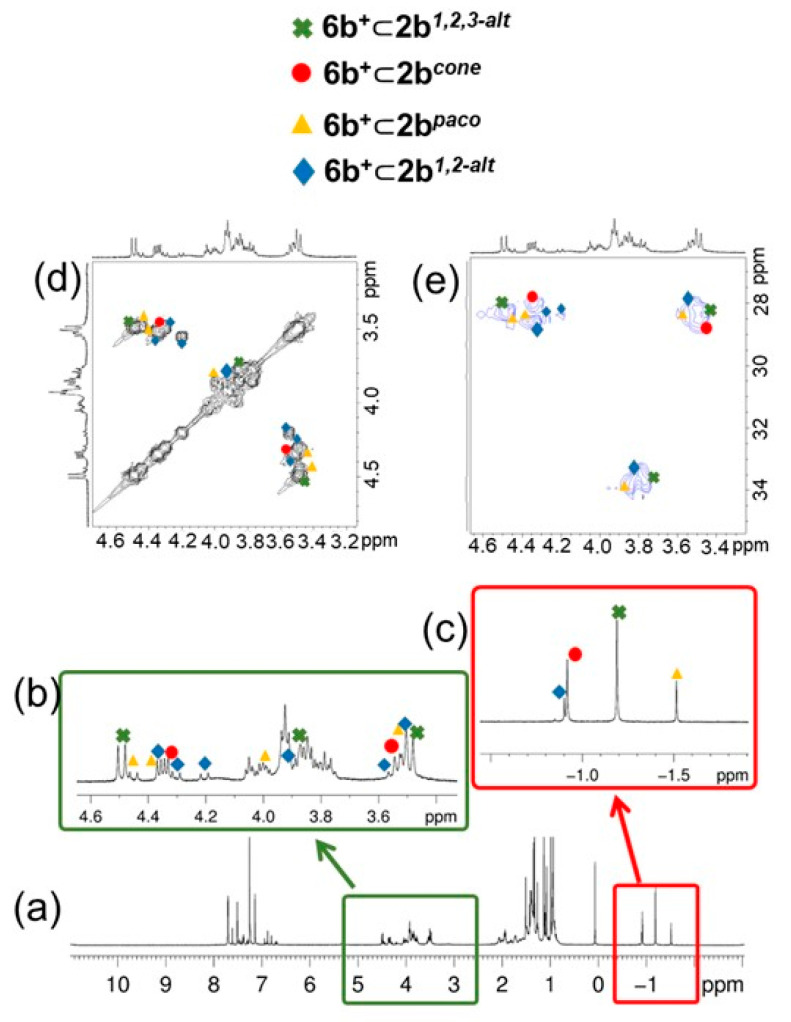
The 1D and 2D NMR spectra (CDCl_3_, 600 MHz, 298 K) for the complexation of **6b^+^** by **2b**. (**a**) ^1^H NMR spectrum of an equimolar solution of **2b** and **6b^+^**[B(ArF)_4_]^–^(3 mM). (**b**,**c**) Enlargements of two different portions of (**a**). (**d**,**e**) Portions of the COSY-45 and HSQC spectra (CDCl_3_, 600 MHz, 298 K) of the solution in (**a**).

With these results in hand, we focused our attention on a branched alkylammonium guest such as *tert*-butylammonium **6b^+^**[B(ArF)_4_]^–^, which revealed a very different behaviour compared to **6a^+^**[B(ArF)_4_]^–^. Close inspection of the ^1^H NMR spectrum (CDCl_3_, 600 MHz, 298 K) of the equimolar mixture **2b**/**6b^+^** (3 mM) in Figure 4 showed typical signals indicative of the *endo*-cavity complexation of **6b^+^** inside the cavity of **2b** (Scheme 2).

Closer inspection of the methylene region (4.5–3.4 ppm) in the COSY-45 spectrum of the equimolar mixture **2b**/**6b^+^** (3 mM) showed the presence of several AX systems (marked in red, blue, yellow, and green in Figure 4), which correspond to the formation of various **6b^+^**⊂**2b** complexes in which the calix[6]arene macrocycle adopts different conformations. The 1D and 2D NMR analyses of the equimolar mixture **2b**/**6b^+^** (3 mM), supported with DFT calculations, made it possible to identify four complexes, namely, **6b^+^**⊂**2b*^1,2,3-alt^***, **6b^+^**⊂**2b*^cone^***, **6b^+^**⊂**2b*^paco^***, and **6b^+^**⊂**2b*^1,2-alt^*** (Scheme 2), in which the calix[6]arene host adopts the 1,2,3-alternate, cone, partial cone, and 1,2-alternate conformations, respectively.

The integration of the ^1^H NMR signals attributable to **6b^+^**⊂**2b*^1,2,3-alt^***, **6b^+^**⊂**2b*^cone^***, **6b^+^**⊂**2b*^paco^***, and **6b^+^**⊂**2b*^1,2-alt^*** revealed that the four complexes were present in a 6:3:2:1 ratio. From these data and through a quantitative ^1^H NMR experiment, using trichloroethylene (TCE) as the internal standard (see the experimental section for further details), the following apparent association constants were calculated: 2.6 ± 0.2 × 10^3^ M^–1^ (**6b^+^**⊂**2b*^1,2,3-alt^***); 1.8 ± 0.2 × 10^3^ M^–1^ (**6b^+^**⊂**2b*^cone^***), 8.1 ± 0.2 × 10^2^ M^–1^ (**6b^+^**⊂**2b*^paco^***), and 4.3 ± 0.2 × 10^2^ M^–1^ (**6b^+^**⊂**2b*^1,2-alt^***).

These values clearly demonstrated that the binding of the branched *tert*-butylammonium cation is generally less favored than that of linear *n*-pentylammonium, probably due to the greater steric encumbrance of the *t*-Bu group.

For the sake of clarity, hereafter, we report on the analysis of the 1D and 2D NMR spectra in Figure 4, supporting the identification of the four complexes formed upon mixing **2b** and **6b^+^** in an equimolar ratio (3 mM) in CDCl_3_ at 298 K.

**6b^+^**⊂**2b*^1,2,3-alt^***: The presence of an ArCH_2_Ar AX system at 3.49/4.49 ppm and an AB system at 3.86/3.77 ppm provides evidence of the **6b^+^**⊂**2b*^1,2,3-alt^*** complex, in which the calix[6]arene adopts a 1,2,3-alternate conformation. The HSQC correlations of these ArCH_2_Ar signals with carbon resonances at 28.3 and 33.7 ppm (Figure 4e) confirmed the presence of the calixarene host in the 1,2,3-alternate conformation, according to Gutsche’s “^1^H NMR” rule [28,30] and the “^13^C NMR single rule” of de Mendoza [29,30]. Finally, the *tert*-butyl singlet of **6b^+^** shielded inside the cavity of **2b*^1,2,3-alt^*** was detected at −1.15 ppm (marked in green), a value significantly up-field-shifted as compared to the analogous signals of **6b^+^** hosted inside the cone-shaped cavity of **6b^+^**⊂**2b*^cone^*** (−0.92 ppm, marked in red) and the **6b^+^**⊂**2b*^1,2-alt^*** complex (−0.91 ppm, marked in blue) (*vide infra*).

**6b^+^**⊂**2b*^cone^***: The methylene region of the ^1^H NMR spectrum in Figure 4a,b showed the presence of an AX system (COSY spectrum, indicated in red in Figure 4d) at 3.52/4.34 ppm (Δ*δ* = 0.82 ppm), which correlated in the HSQC spectrum (Figure 4e) with a carbon resonance at 28.0 ppm, attributable to carbon atoms between *syn*-oriented aromatic rings. These signals can be assigned to the **6b^+^**⊂**2b*^cone^*** complex, in which calix[6]arene **2b** adopts the cone conformation. Interestingly, the *tert*-butyl signal of **6b^+^** shielded inside the aromatic cavity of **2b*^cone^*** was found at −0.92 ppm (marked in red in Figure 4c).

**6b^+^**⊂**2b*^paco^***: The ^1^H NMR spectrum in Figure 4a,b showed the presence of two less intense AX systems (COSY spectrum, Figure 4d) in a 1:1 ratio at 3.48/4.45 ppm (Δ*δ* = 0.97 ppm) and 3.51/4.35 ppm (Δ*δ* = 0.84 ppm), which correlated in the HSQC spectrum (Figure 4e) with carbon resonances at 28.3 and 28.6 ppm, respectively, attributable to ArCH_2_Ar carbon atoms between *syn*-oriented aryl rings. In addition, an AB system (COSY spectrum) was detected at 3.86/3.99 ppm, attributable to ArCH_2_Ar groups between *anti*-oriented aryl rings, which correlated with a carbon resonance at 33.8 ppm. According to the application of Gutsche’s and de Mendoza’s rules, these data were indicative of the presence of a **6b^+^**⊂**2b*^paco^*** complex in which the calix[6]arene adopted the partial cone conformation. Additionally, in this case, a singlet was detected at −1.51 ppm (marked in yellow in Figure 4), attributable to the -C(C*H*_3_)_3_ group of 6b+ shielded inside the cavity of 2b***^paco^***.

**6b^+^**⊂**2b*^1,2-alt^***: The presence of the **6b^+^**⊂**2b*^1,2-alt^*** complex was confirmed by three ArCH_2_Ar AX systems (COSY spectrum, Figure 4d) at 3.49/4.30, 3.51/4.20, and 3.51/4.36 ppm (Δ*δ* = 0.81, 0.69, and 0.85 ppm, respectively) in a 1:1:2 ratio, which correlated in the HSQC spectrum with carbon signals at 28.5, 28.6, and 28.4 ppm, respectively, and an AB ArCH_2_Ar system at 3.86/3.96 ppm, which correlated with a carbon resonance at 33.6 ppm. In this case, in the negative region of the ^1^H NMR, we observed the *tert*-butyl singlet of **6b^+^** at –0.90 ppm (marked in blue in Figure 4c).

To investigate the energy contribution of noncovalent interactions, a SOPT analysis of the Fock matrix in the NBO basis was carried out on the DFT-optimized structures of the four **6b^+^**⊂**2b** complexes.

**Figure 5:**
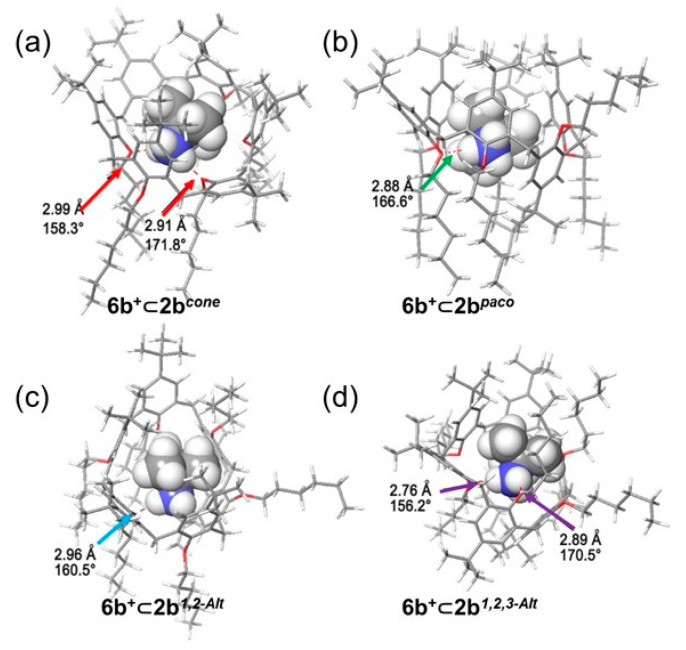
DFT-optimized structures of the complexes **6b^+^**⊂**2b^1,2,3-alt^** (**a**), **6b^+^**⊂**2b^cone^** (**b**), **6b^+^**⊂**2b^paco^** (**c**), and **6b^+^**⊂**2b^1,2-alt^** (**d**) calculated on the B3LYP/6-31G(d,p) theoretical level.

The DFT-optimized structure of the **6b^+^**⊂**2b*^1,2,3-alt^*** complex indicated the presence of stabilizing H-bonding and C−H∙∙∙π interactions between the guest **6b^+^** **and** **2b*^1,2,3-alt^***. A SOPT analysis indicated that the stabilization energy was mainly due to the formation of two ^+^N–H···O*^2b^* H-bonding interactions, which contributed 80% of the total binding energy (Table 1). This value is significantly higher than that calculated for the ^+^N−H···O***^2b^*** H−bonding interactions of the **6b^+^**⊂**2b*^cone^*** complex, which was 61% of the total energy of non-covalent interactions (Table 1) for this complex. The DFT-optimized structure of the **6b^+^**⊂**2b*^cone^*** complex calculated on the B3LYP/6-31G(d,p) theoretical level (Figure 5a) showed the presence of two ^+^N−H···O interactions with an average ^+^N···O***^2b^*** distance of 2.95 Å, being longer and weaker than that calculated for the **6b^+^**⊂**2b*^1,2,3-alt^*** complex (^+^N···O***^2b^*** average distance of 2.80 Å), while an average ^+^N–H···O***^2b^*** angle of 165.1° was calculated for the **6b^+^**⊂**2b*^cone^*** complex (163.3° calculated for **6b^+^**⊂**2b*^1,2,3-alt^***). Additionally, C−H∙∙∙π interactions were detected between the *tert*-butyl group of 6b+ and the aromatic rings of **2b**, with an average C-H···π centroid distance of 2.95 Å and an average C-H···π centroid angle of 153.3°.

Concerning the DFT-optimized structure of **6b^+^**⊂**2b*^paco^*** (Figure 5b), a single H-bonding interaction was detected between the NH_3_^+^ group of 6b+ and an oxygen atom of **2b*^paco^***, with a distance of 2.88 Å and an angle of 166.6°. The SOPT analysis revealed a lower value for the contribution of the ^+^N–H···O***^2b^*** hydrogen-bonding interaction (53%, Table 1).

Similarly, the DFT-optimized structure of the **6b^+^**⊂**2b*^1,2-alt^*** complex (Figure 5c) suggested the existence of a single H-bonding interaction between the ammonium group of 6b+ and an oxygen atom of **2b*^1,2-alt^***. Here, the SOPT analysis also revealed a lower 36% contribution of the ^+^N–H···O***^2b^*** hydrogen-bonding interaction (Table 1) to the total binding energy.

These results clearly indicate that the *tert*-butylammonium guest **6b^+^** prefers the 1,2,3-alt–**2b** as the best-fitting host conformation to a greater extent than the other conformations. In addition, the NCI analysis suggests that the stabilization induced by the H-bonding interactions between the ammonium group of **6b^+^** and the oxygen atoms of the calixarene **2b** (^+^N–H···O) plays a crucial role in determining the thermodynamic stabilities of the four complexes shown in Scheme 2. A careful comparison of the DFT-optimized structures of the **6a^+^**⊂**2b** and **6b^+^**⊂**2b** complexes reveals significant differences in the binding modes of the two guests. In particular, the greater steric requirements of the *tert*-butyl group of **6b^+^** force it to occupy a deeper position inside the calix[6]arene cavity (in each of the four conformations), leading to greater deformation of the host, which, in turn, implies a higher energetical cost and a lower binding constant. In this way, the deeply positioned **6b^+^** guest is able to form two stabilizing H-bonds with the 1,2,3-alt- and cone-**2b** conformations, whereas only one H-bond is possible with the other paco- and 1,2-alt-**2b** isomers.

### 2.3. Binding Ability of ***2b*** toward Isopropylammonium Guest ***6c***^+^[B(ArF)_4_]^–^

**Figure 6:**
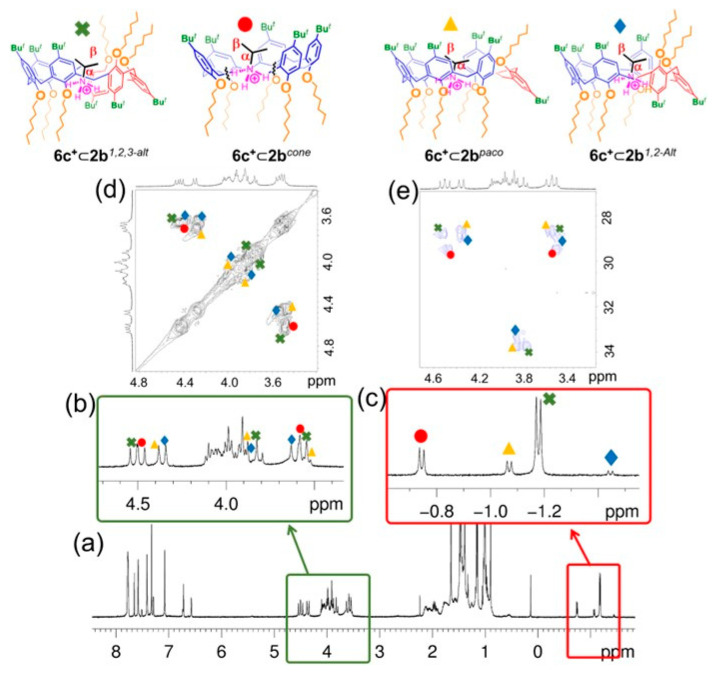
The 1D and 2D NMR spectra (CDCl_3_, 600 MHz, 298 K): (**a**) ^1^H NMR spectrum of an equimolar solution of **2b** and **6b^+^**[B(ArF)_4_]^–^ (3 mM); (**b**,**c**) enlargements of two different portions of (**a**); (**d**,**e**) portions of the COSY-45 and HSQC spectra (CDCl_3_, 600 MHz, 298 K) of the solution in (**a**).

The *endo*-cavity complexation of the isopropylammonium guest **6c^+^**[B(ArF)_4_]^–^ showed very similar features to those observed in the complexation of the *tert*-butylammonium guest **6b^+^** with **2b**. In fact, the 1D and 2D NMR analysis of an equimolar mixture of **6c^+^**[B(ArF)_4_]^–^ and **2b** resulted in the formation of a well-defined mixture of stereoisomeric complexes in which the calix[6]arene adopted different conformations, namely, **6c^+^**⊂**2b*^1,2,3-alt^***, **6c^+^**⊂**2b*^cone^***, **6c^+^**⊂**2b*^paco^***, and **6c^+^**⊂**2b*^1,2-alt^*** (Figure 6). The four complexes were found in a 9:3:2:1 ratio according to the integration of their ^1^H NMR signals.

Through a quantitative ^1^H NMR (CDCl_3_, 600 MHz, 298 K) experiment, using TCE as the internal standard, four binding constants were calculated for the slowly exchanging complexes: 3.1 ± 0.2 × 10^4^ M^−1^ for **6c^+^**⊂**2b*^1,2,3-alt^***, 9.1 ± 0.2 × 10^3^ M^−1^ for **6c^+^**⊂**2b*^cone^***, 6.1 ± 0.2 × 10^3^ M^−1^ for **6c^+^**⊂**2b*^paco^***, and 1.2 ± 0.2 × 10^3^ M^−1^ for **6c^+^**⊂**2b*^1,2-alt^*** (SI).

These results clearly confirmed the role of the steric encumbrance, since the binding of the more branched *tert*-butylammonium cation is less favored than that of the less branched *i*-propylammonium, which, in turn, is less favored with respect to linear *n*-pentylammonium.

The ^1^H NMR spectrum (600 MHz, 298 K, Figure 6a–c) of a 1:1 mixture of **6c^+^**[B(ArF)_4_]^–^and **2b** (3 mM) in CDCl_3_ showed a series of doublets shielded at the negative values of chemical shifts and attributable to *endo*-cavity complexed *β–*CH_3_ groups of 6c+: −1.50 (*J* = 6.8 Hz), −1.23 (*J* = 6.8 Hz), −1.13 (*J* = 6.8 Hz), −0.68 (*J* = 6.8 Hz) ppm, assigned to **6c^+^**⊂**2b*^1,2-alt^***, **6c^+^**⊂**2b*^1,2,3-alt^***, **6c^+^**⊂**2b*^paco^***, and **6c^+^**⊂**2b*^cone^***, respectively.

**6c^+^**⊂**2b*^1,2,3-alt^***: The formation of the **6c^+^**⊂**2b*^1,2,3-alt^*** complex, in which the calix[6]arene adopts a 1,2,3-alternate conformation, was ascertained according to the presence of an AX system at 3.47/4.46 ppm (Δ*δ* = 1.00 ppm; HSQC: ^1^*J* correlation with an Ar^13^*C*H_2_Ar signal at 28.3 ppm) and an AB system at 3.73/3.81 ppm (Δ*δ* = 0.08 ppm; HSQC: ^1^*J* correlation with an Ar*C*H_2_Ar signal at 34.0 ppm, marked in green in Figure 6).

**6b^+^**⊂**2b*^cone^***: The formation of the **6b^+^**⊂**2b***^cone^* complex was established based on the presence of an AX system at 3.48/4.41 ppm, which correlated in the HSQC spectrum (Figure 6e) with a carbon resonance at 28.0 ppm, attributable to carbon atoms between *syn*-oriented aromatic rings (red marked in Figure 6).

**6c^+^**⊂**2b*^paco^***: The ^1^H NMR and COSY-45 spectra of the **6c^+^**⊂**2b*^paco^*** complex showed two AX systems at 3.54/4.29 ppm (Δ*δ* = 0.75) and 3.53/4.29 ppm (Δ*δ* = 0.76) in 1:1 ratio, which correlated in the HSQC spectrum (Figure 6e) with carbon resonances at 28.3 and 28.6 ppm, respectively. Moreover, the HSQC spectrum of the complex showed a correlation between a carbon resonance at 34.0 ppm and an AB system at 3.81/4.01) ppm, attributable to ArCH_2_Ar groups between *anti*-oriented Ar rings.

**6c^+^**⊂**2b*^paco^***: The presence of three ArCH_2_Ar AX systems (COSY spectrum, Figure 6d) at 3.47/4.40, 3.52/4.34, and 3.57/4.20 ppm (Δ*δ* = 0.93, 0.82, and 0.63 ppm, respectively) in a 1:1:2 ratio (indicated in blue in Figure 6) is compatible with the presence of the **6c^+^**⊂**2b*^1,2-alt^*** complex.

The H-bonding contribution to the total binding energy calculated for the four **6c^+^**⊂**2b** complexes (Table 2) is in agreement with their thermodynamic stability order evaluated based on *K*_ass_ values in CDCl_3_ at 298 K: **6c^+^**⊂**2b*^1,2,3-alt^*** > **6c^+^**⊂**2b*^cone^*** > **6c^+^**⊂**2b*^paco^*** > **6c^+^**⊂**2b*^1,2-alt^***. These results clearly indicate that, once again, the 1,2,3-alt-**2b** was selected as the best-fitting host conformation in the presence of a branched alkylammonium guest such as **6c^+^**. The stability order of the **6c^+^**⊂**2b** complexes is in full agreement with that observed in the presence of the *tert*-butylammonium guest 6b+. The natural bond orbital and noncovalent interaction analyses indicate that H-bonding interactions between the ammonium group of **6c^+^** and the oxygen atoms of the calixarene **2b** (^+^N–H···O) provide the key stabilization factor for the **6c^+^**⊂**2b** complexes. In fact, they account for 87%, 77%, 66%, and 52% of the total binding energy for the **6c^+^**⊂**2b*^1,2,3-alt^***, **6c^+^**⊂**2b*^cone^***, **6c^+^**⊂**2b*^paco^***, and **6c^+^**⊂**2b*^1,2-alt^*** complexes, respectively (Table 2).

At this point, it was important to verify whether the thermodynamic stability of the individual conformational complexes was in accordance with the Expanding Coefficient (EC) parameter recently proposed by our group [34]. In fact, it was found that the EC parameter can be conveniently correlated with the thermodynamic stability of supramolecular complexes obeying the induced-fit or conformational selection models and governed by weak secondary interactions. EC is defined as the ratio between the volume of the host cavity after complexation and that of the host cavity before complexation [34].

The EC values were thus calculated from the cavity volumes of the complexed and free host using the above DFT-optimized structures and that of the 1,2,3-alternate ground 2b host for all the **6b^+^**⊂**2b** and **6c^+^**⊂**2b** complexes (SI) with the Caver software [35,36,37]. In detail, taking the **6c^+^**⊂**2b** complexes as an example, the EC values of 6.03, 6.37, 4.84, and 6.03 were found for **6c^+^**⊂**2b^1,2,3-alt^**, **6c^+^**⊂**2b*^cone^***, **6c^+^**⊂**2b*^paco^***, and **6c^+^**⊂**2b^1,2*-alt*^**, respectively, whose log K_app_ values were 4.49, 3.96, 3.79, and 3.08, respectively (SI). As is clearly evident, the previously observed linear correlation between the EC and log K_app_ [34] is not a factor here [30]. This can be easily explained by considering the number of H-bonding interactions, which, as stated above, is the main determinant of the thermodynamic stability of these complexes. Thus, if we only consider those complexes with two H-bonding interactions, **6c^+^**⊂**2b^1,2,3-*alt*^** (EC = 6.03, log K_app_ = 4.49) and **6c^+^**⊂**2b*^cone^***, (EC = 6.37, log K_app_ = 3.96), we find a good correlation between an increasing EC and decreasing log K_app_. In the same way, considering only those complexes with one H-bonding interaction, **6c^+^**⊂**2b*^paco^*** (EC = 4.84, log K_app_ = 3.79) and **6c^+^**⊂**2b^1,2-*alt*^** (EC = 6.03, log K_app_ = 3.08), we again find a good correlation between an increasing EC and decreasing log K_app_. Therefore, these findings are in accordance with our previous statement [34] that “the EC parameter can be considered of general applicability in all those instances in which no new strong intermolecular interactions (e.g., H-bonds) are generated during the induced-fit process”.

## 3. Conclusions

This study clearly shows that hexahexyloxycalix[6]arene **2b** can complex alkylammonium guests and shows a conformational adaptive behavior. Thus, in the presence of *n-*pentylammonium **6a^+^**, the cone-**2b** is the best-fitting conformation at the expense of the 1,2,3-alternate-**2b**, which is the most abundant conformer in the absence of a guest. In a different way, branched alkylammonium guests, such as *tert*-butylammonium **6b^+^** and isopropylammonium **6c^+^**, select a combination of conformers of **2b**. Four complexes were revealed and characterized using 1D and 2D NMR spectra, in which **2b** adopted different conformations, namely, **6b^+^**/**6c^+^**⊂**2b^1,2,3-*alt*^**, **6b^+^**/**6c^+^**⊂**2b*^cone^***, **6b^+^**/**6c^+^**⊂**2b*^paco^***, and **6b^+^**/**6c^+^**⊂**2b^1,2*-alt*^**. The binding constant values determined through NMR experiments indicated that the 1,2,3-alternate was the best-fitting **2b** conformation for the complexation of branched alkylammonium guests, followed by cone > paco > 1,2-alt. The NCI and NBO calculations suggest that the H-bonding interactions between the ammonium group of the guest and the oxygen atoms of the calixarene **2b** (^+^N–H···O) played a crucial role in determining the thermodynamic stability order of the four complexes. In addition, it was found that an increase in branching from *n*-Pent to *i*-Pr and in *t*-Bu groups leads to a corresponding decrease in binding affinity. Therefore, it can be concluded that higher steric encumbrance leads to weaker H-bonding interactions and, hence, to a lower binding energy.

## Figures and Tables

**Table 1:** Contribution of ^+^N–H···O hydrogen-bonding interactions to the total binding energy, as calculated via SOPT analysis of the Fock matrix in the natural bond orbital (NBO) basis for the complexes between **6b^+^** and **2b** in different conformations.

Complex	K*_ass_*	^+^N–H···O H-Bonding Percentage of Total Binding Energy (%)
**6b^+^**⊂**2b*^1,2,3-alt^***	2.6 ± 0.2 × 10^3^ M^–1^	80
**6b^+^** **⊂**2b***^cone^***	1.8 ± 0.2 × 10^3^ M^–1^	61
**6b^+^**⊂**2b*^paco^***	8.1 ± 0.2 × 10^2^ M^–1^	53
**6b^+^**⊂**2b*^1,2-alt^***	4.3 ± 0.2 × 10^2^ M^–1^	36

**Table 2:** Contribution of ^+^N–H···O hydrogen-bonding interactions to the total binding energy, as calculated via SOPT analysis of the Fock matrix in the natural bond orbital (NBO) basis for the complexes between 6c+ and 2b in different conformations.

Complex	K*_ass_*	^+^N–H···O H-Bonding Percentage of Total Binding Energy (%)
**6c^+^**⊂**2b*^1,2,3-alt^***	3.1 ± 0.2 × 10^4^ M^–1^	87
**6c^+^**⊂**2b*^cone^***	9.1 ± 0.2 × 10^3^ M^–1^	77
**6c^+^**⊂**2b*^paco^***	6.1 ± 0.2 × 10^3^ M^–1^	66
**6c^+^**⊂**2b*^1,2-alt^***	1.2 ± 0.2 × 10^3^ M^–1^	52

## Data Availability

Not applicable.

## Supplementary Materials

The following supporting information can be downloaded at: https://www.mdpi.com/article/10.3390/molecules28124749/s1, Supplementary Materials containing the procedure for the formation of alkylammonium@calixarene complexes; 1D and 2D NMR spectra of complexes (Figures S1–S21); details of DFT, NBO/NCI, calculations and stability constant determination [38].
