# Supplementary material for: Hexahexyloxycalix[6]arene, a Conformationally Adaptive Host for the Complexation of Linear and Branched Alkylammonium Guests
Source: Molecules. 2023 Jun 13;28(12):4749. doi: 10.3390/molecules28124749 (PMC10304928; doi:10.3390/molecules28124749)
Supplement: Supplementary file 1 [file molecules-28-04749-s001.zip › molecules-2416041-supplementary.pdf]

Supplementary Information

Hexahexyloxycalix[6]arene, a Conformationally Adaptive Host for the Complexation of Linear and Branched Alkylammonium Guests

Veronica Iuliano, Carmen Talotta,* Paolo Della Sala, Margherita De Rosa, Annunziata Soriente, Placido Neri and Carmine Gaeta,*

Dipartimento di Chimica e Biologia "A. Zambelli", Università di Salerno, Via Giovanni Paolo II 132, I-4084 Fisciano (Salerno), Italy.

* Correspondence: ctalotta@unisa.it (C.T.); cgaeta@unisa.it (C. G.).

Table of Contents

Formation of pseudo[2]rotaxanes S3
^{1}H NMR spectrum of complex **6a^{+}⊂2b** S4
2D COSY spectrum of complex **6a^{+}⊂2b** S5
2D HSQC spectrum of complex **6a^{+}⊂2b** S6
^{1}H NMR spectrum of complex **6b^{+}⊂2b** S7
2D COSY spectrum of complex **6b^{+}⊂2b** S8
2D HSQC spectrum of complex **6b^{+}⊂2b** S9
^{1}H NMR spectrum of complex **6c^{+}⊂2b** S10
2D COSY spectrum of complex **6c^{+}⊂2b** S11
2D HSQC spectrum of complex **6c^{+}⊂2b** S12
^{1}H NMR determination of K_{ass} values S13
Determination of K_{ass} values for complex **6a^{+}⊂2b** S14
Determination of K_{ass} values for complex **6b^{+}⊂2b** S15
Determination of K_{ass} values for complex **6c^{+}⊂2b** S16
Computational Studies S17
References S47

Formation of ammonium complexes.

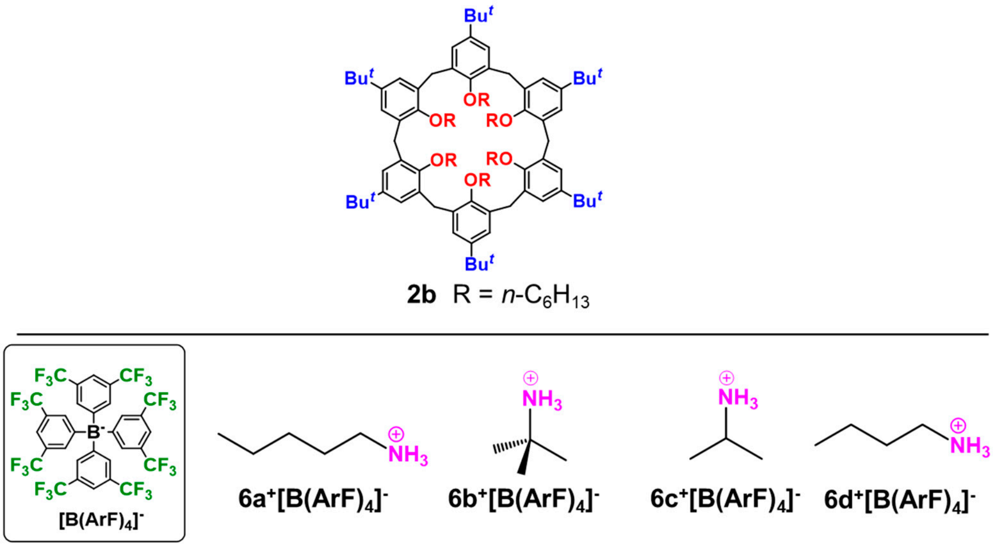

Scheme S1.

General procedure:

A 1:1 mixture of calix[6]arene **2b** and alkylammonium cation **6a-c**$^{+}$ as barfate salt, was dissolved in 0.4 mL of $CDCl_3$. We report in **Table S1** the concentration used for the preparation of the complexes. Then, the solution was transferred in an NMR tube for 1D and 2D NMR acquisition.

Table S1.

**Pseudo[2] rotaxanes**	**Calix[6]arene, 2b**		**Axles, 6a-c^{+}**		**$CDCl_3$**
	mg	mmol	mg	mmol	ml
**6a^{+}⊂2b**	2.00	1.56 x 10^{-3}	1.29	1.56 x 10^{-3}	0.4
**6b^{+}⊂2b**	2.00	1.56 x 10^{-3}	1.27	1.56 x 10^{-3}	0.4
**6c^{+}⊂2b**	2.00	1.56 x 10^{-3}	1.25	1.56 x 10^{-3}	0.4

^{1}H NMR spectrum of complex 6a^{+}⊂2b

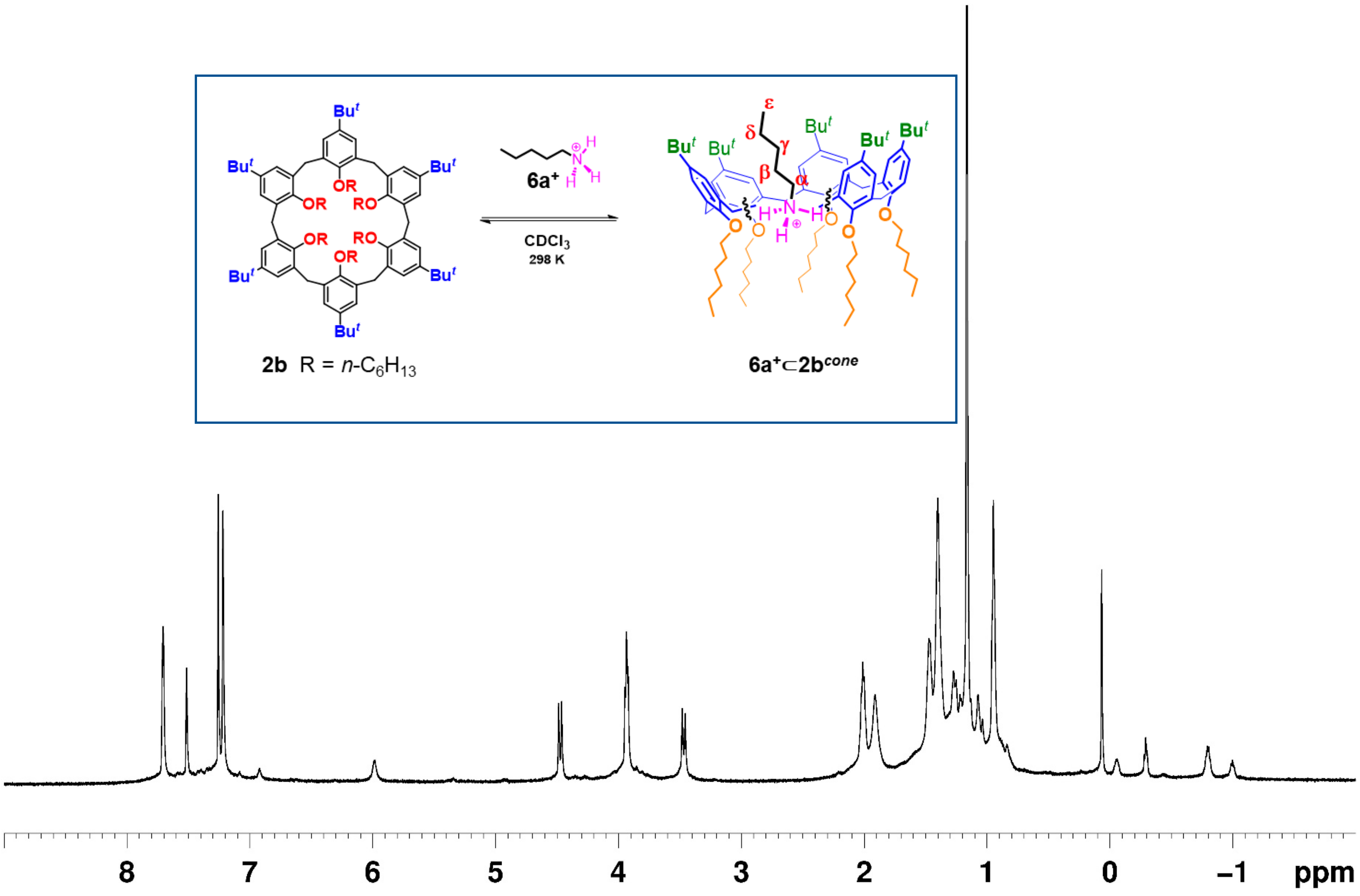

Figure S1. ^{1}H NMR spectrum of **6a^{+}⊂2b** complex (600 MHz, $CDCl_3$, 298 K).

2D COSY spectrum of 6a$^+$⊂2b^{cone} complex

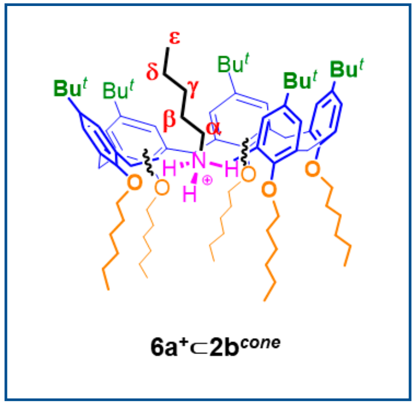

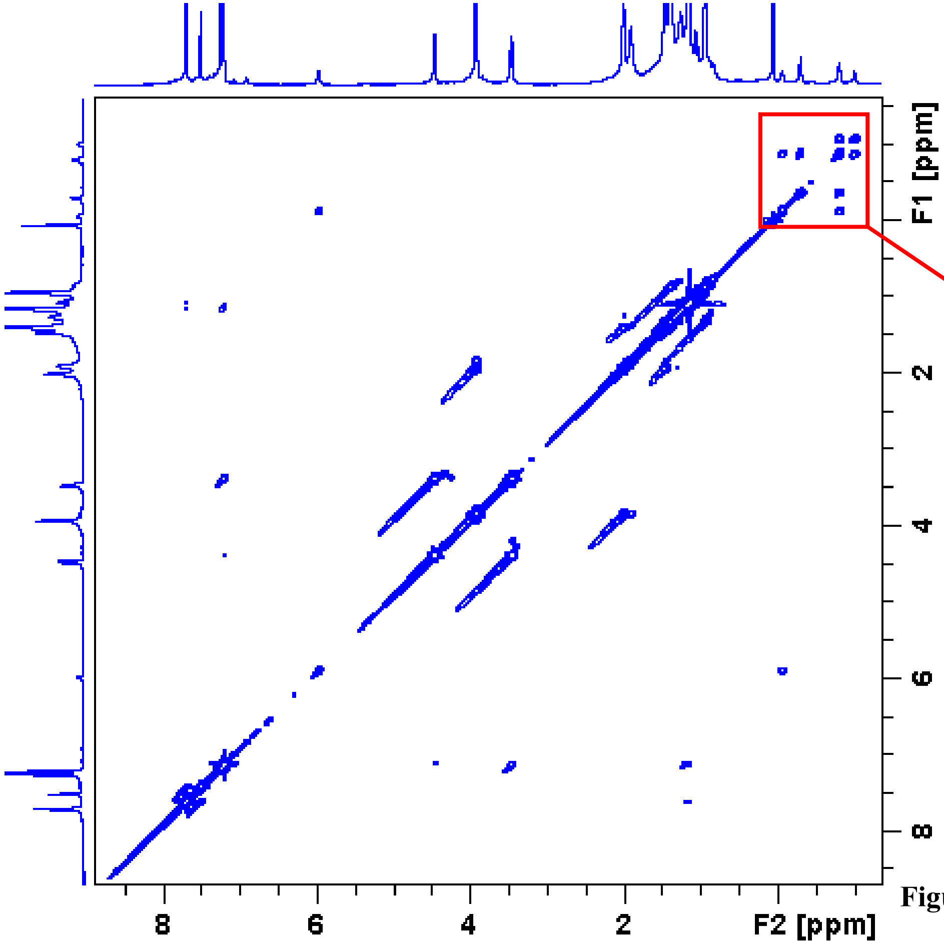

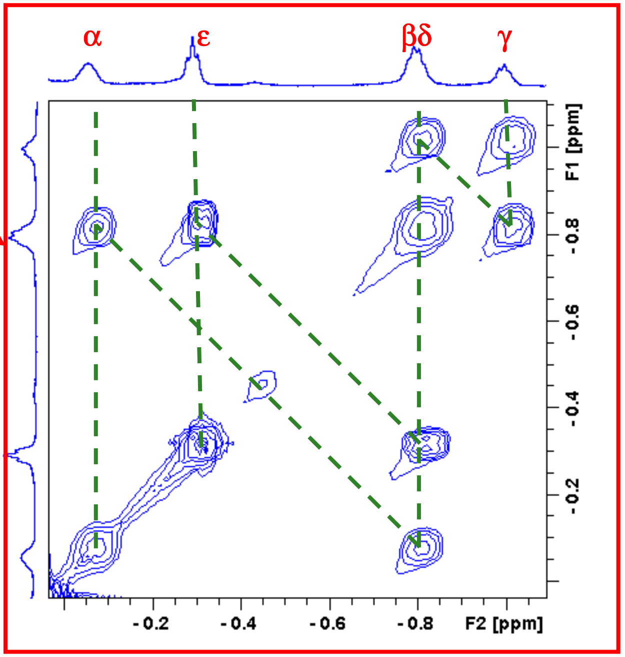

Figure S3. Portion of 2D COSY spectrum of **6a$^+$⊂2b^{cone}** complex (600 MHz, $CDCl_3$, 298 K).

Figure S2. 2D COSY spectrum of **6a$^+$⊂2b^{cone}** complex (600 MHz, $CDCl_3$, 298 K).

2D HSQC spectrum of $6a^{+}\subset 2b^{cone}$ complex

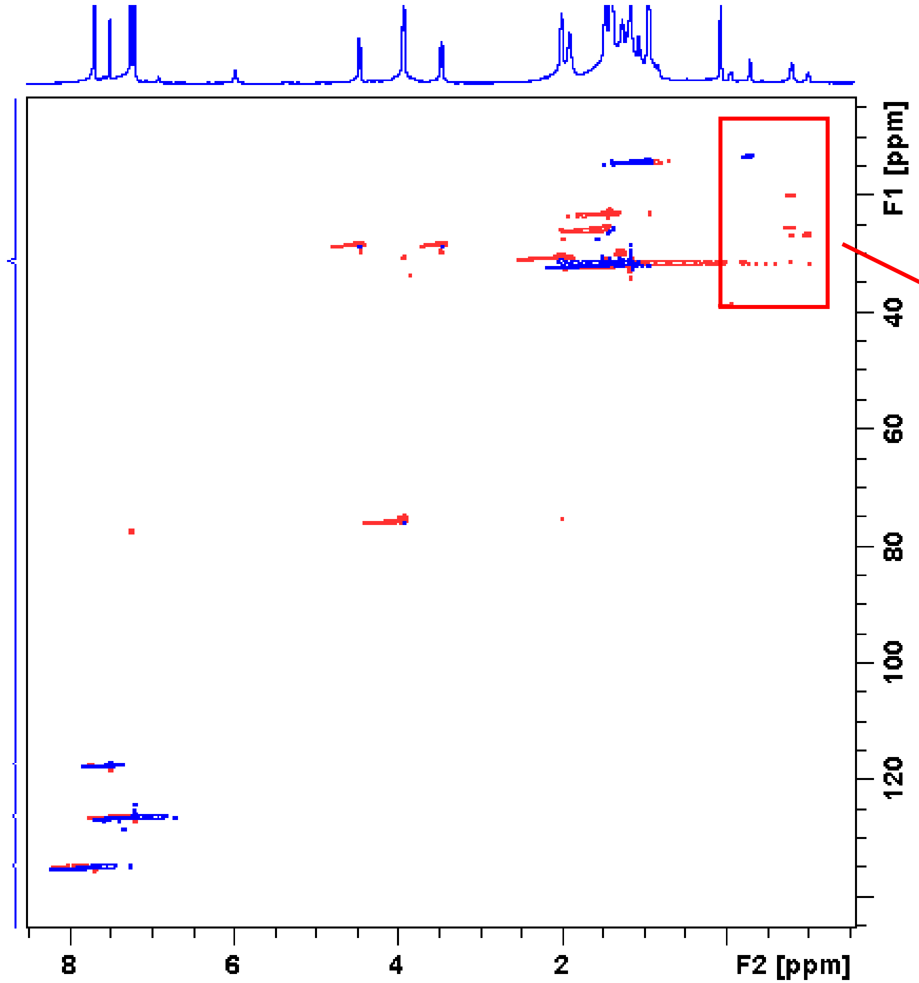

Figure S4. 2D HSQC spectrum of $\mathbf{6a^{+}\subset 2b}^{cone}$ complex (600 MHz, $CDCl_3$, 298 K).

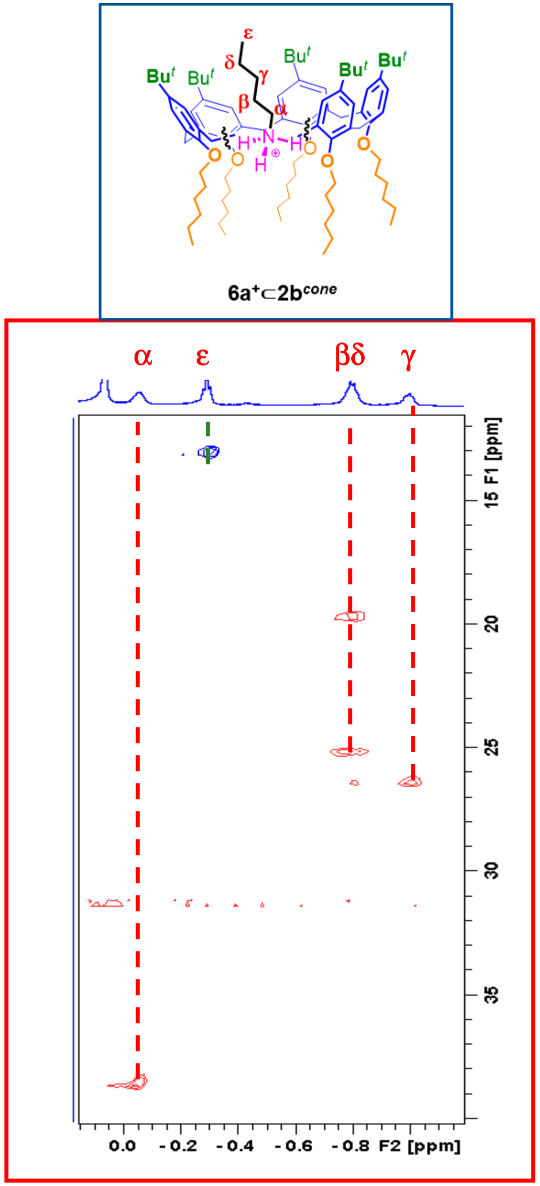

Figure S5. Portion of 2D HSQC spectrum of $\mathbf{6a^{+}\subset 2b}^{cone}$ complex (600 MHz, $CDCl_3$, 298 K).

^{1}H NMR spectrum of 6b$^+$⊂2b complex

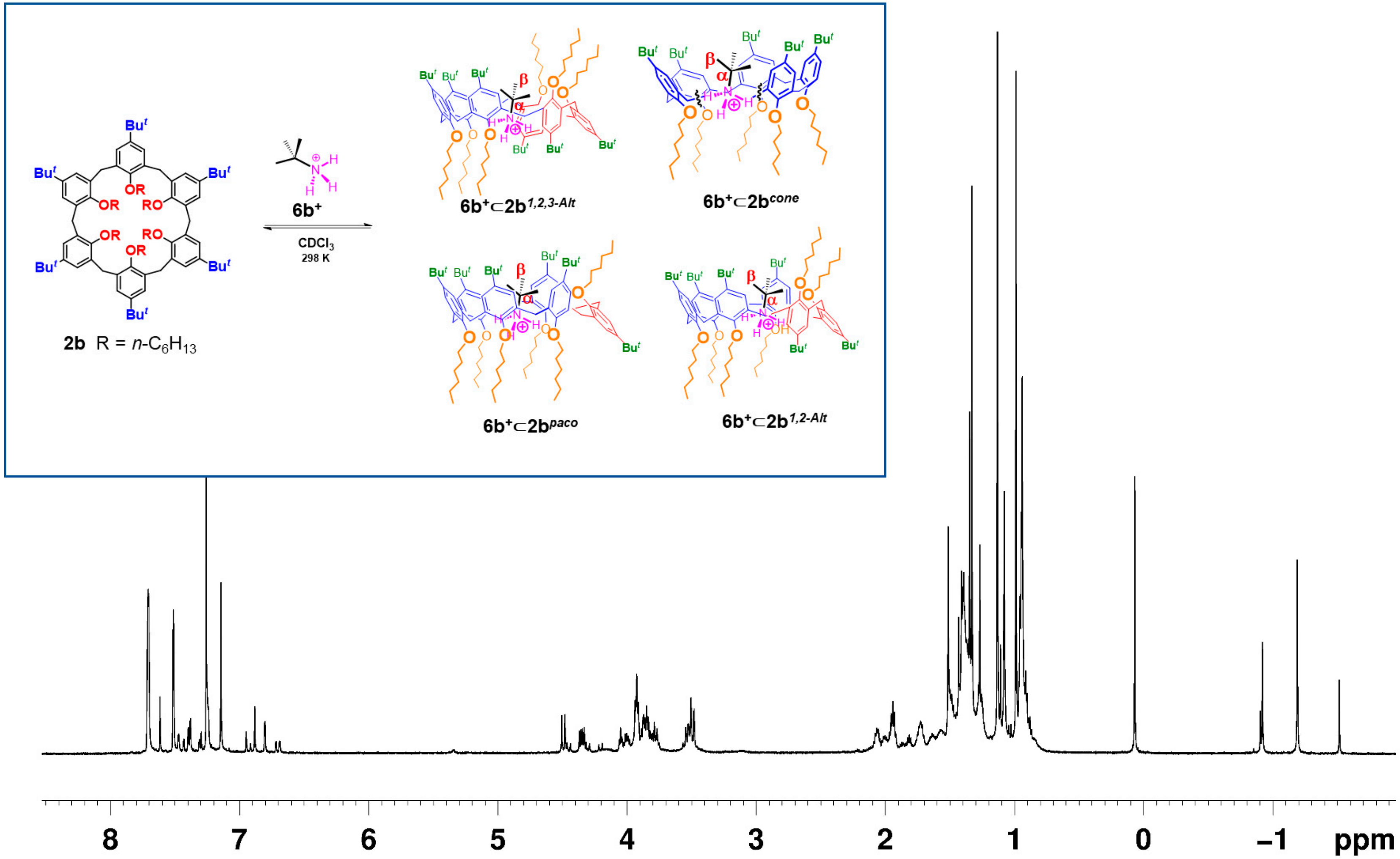

Figure S6. ^{1}H NMR spectrum of **6b$^+$⊂2b** complex (600 MHz, $CDCl_3$, 298 K).

2D COSY spectrum of 6b$^+$⊂2b complex

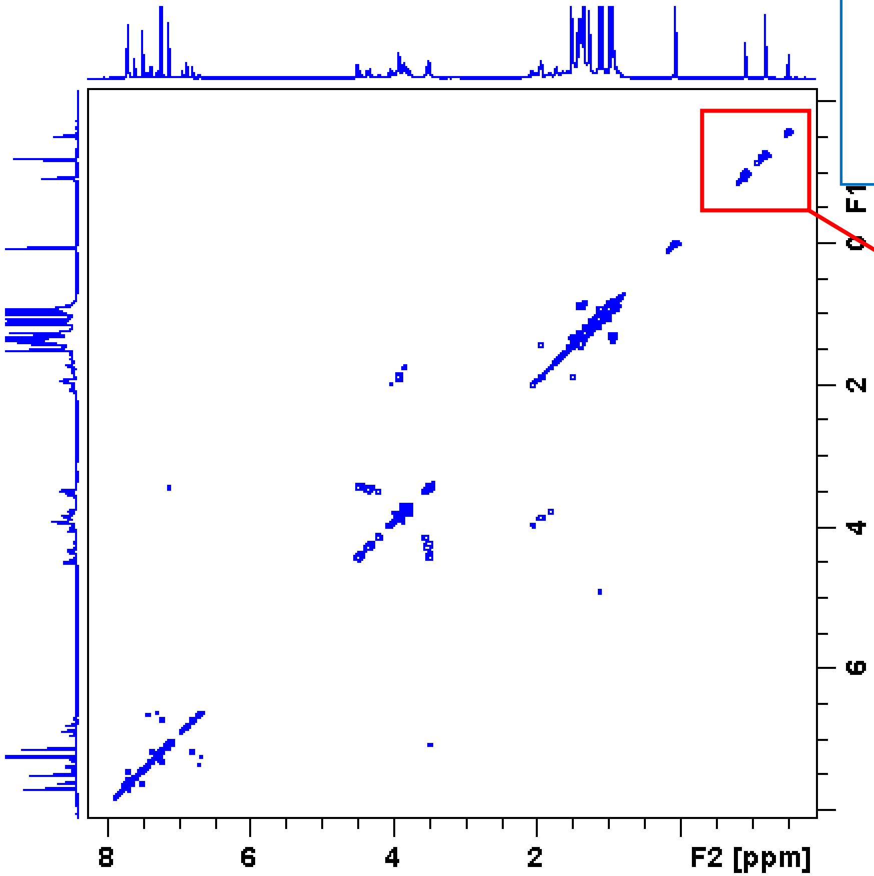

Figure S7. 2D COSY spectrum of **6b$^+$⊂2b** complex (600 MHz, $CDCl_3$, 298 K).

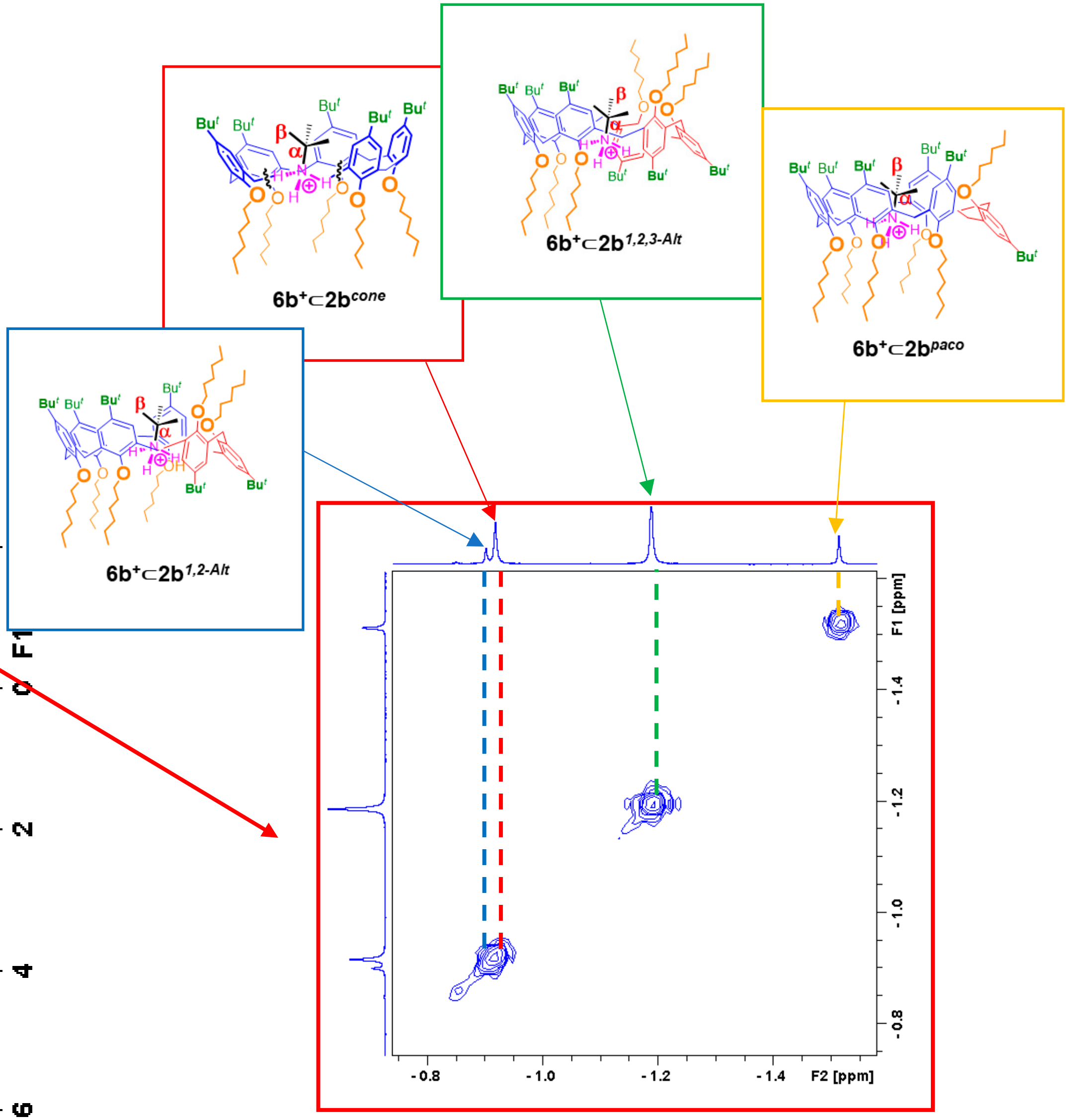

Figure S8. Portion of 2D COSY spectrum of **6b$^+$⊂2b** complex (600 MHz, $CDCl_3$, 298 K).

2D HSQC spectrum of complex 6b^{+}⊂2b

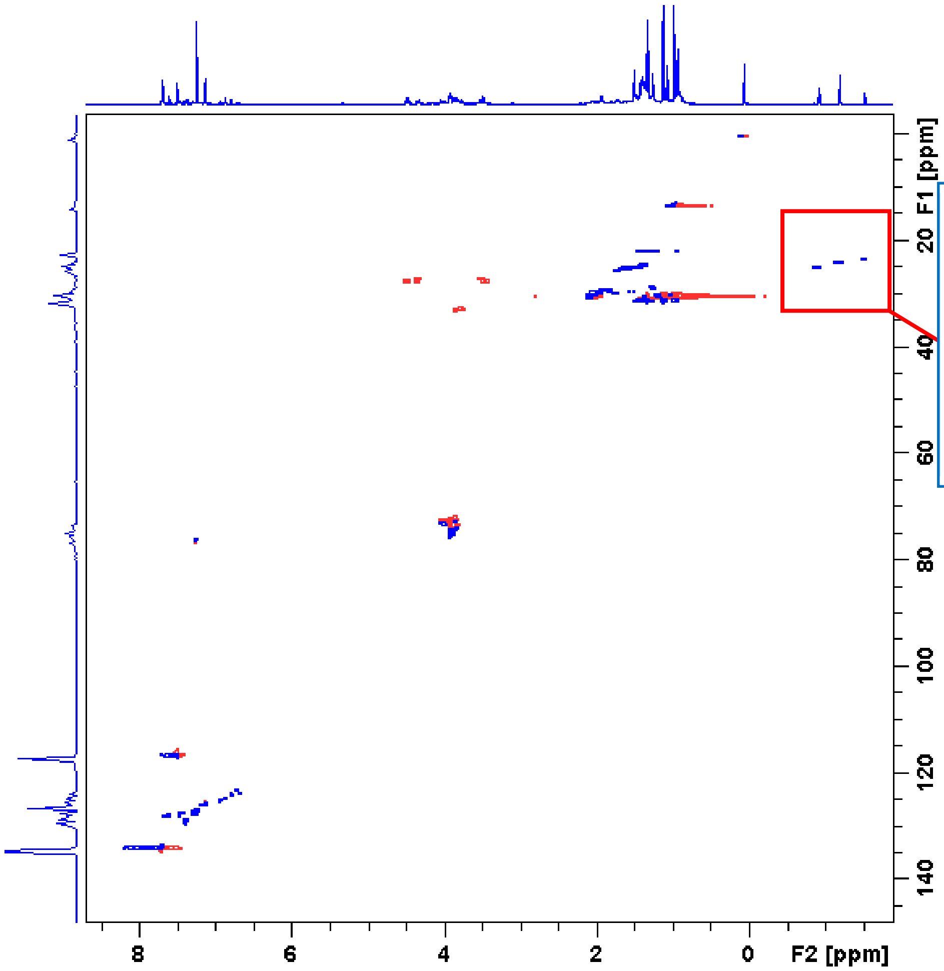

Figure S9. 2D HSQC spectrum of **6b^{+}⊂2b** (600 MHz, CDCl$_3$, 298 K).

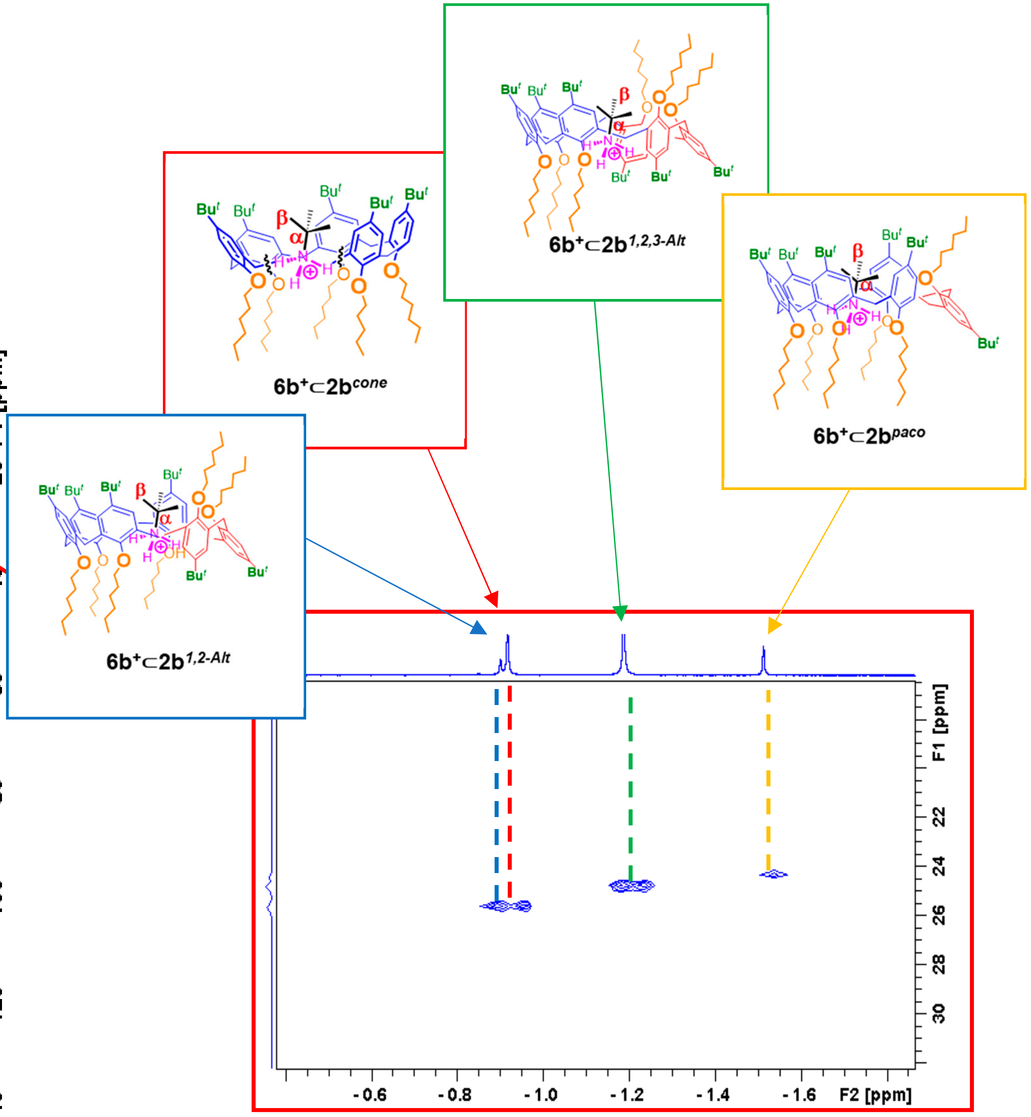

Figure S10. Portion of 2D HSQC spectrum of **6b^{+}⊂2b** (600 MHz, CDCl$_3$, 298) K).

^{1}H NMR spectrum of 6c$^+$⊂2b

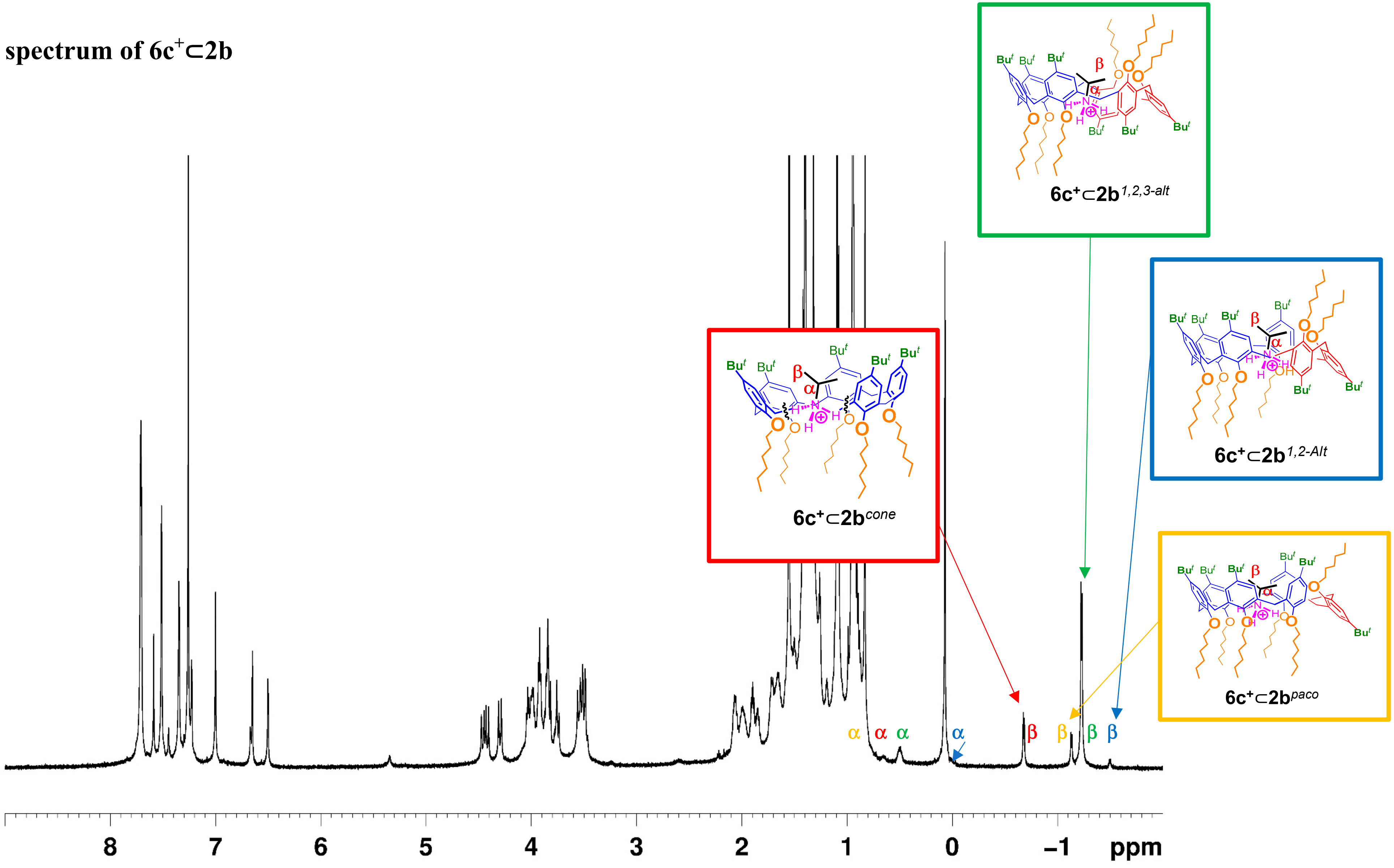

Figure S11. ^{1}H NMR spectrum of **6c$^+$⊂2b** (600 MHz, $CDCl_3$, 298 K).

2D COSY spectrum of 6c$^+$⊂2b

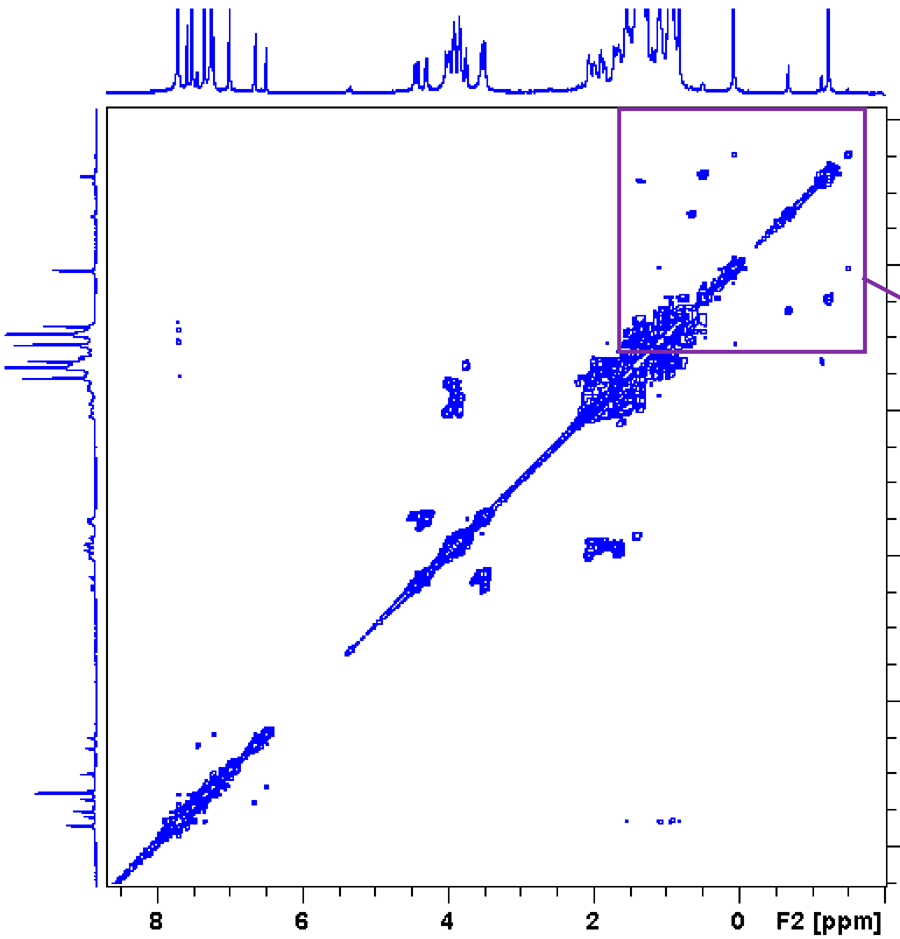

Figure S12. 2D COSY spectrum of **6c$^+$⊂2b** (600 MHz, $CDCl_3$, 298 K).

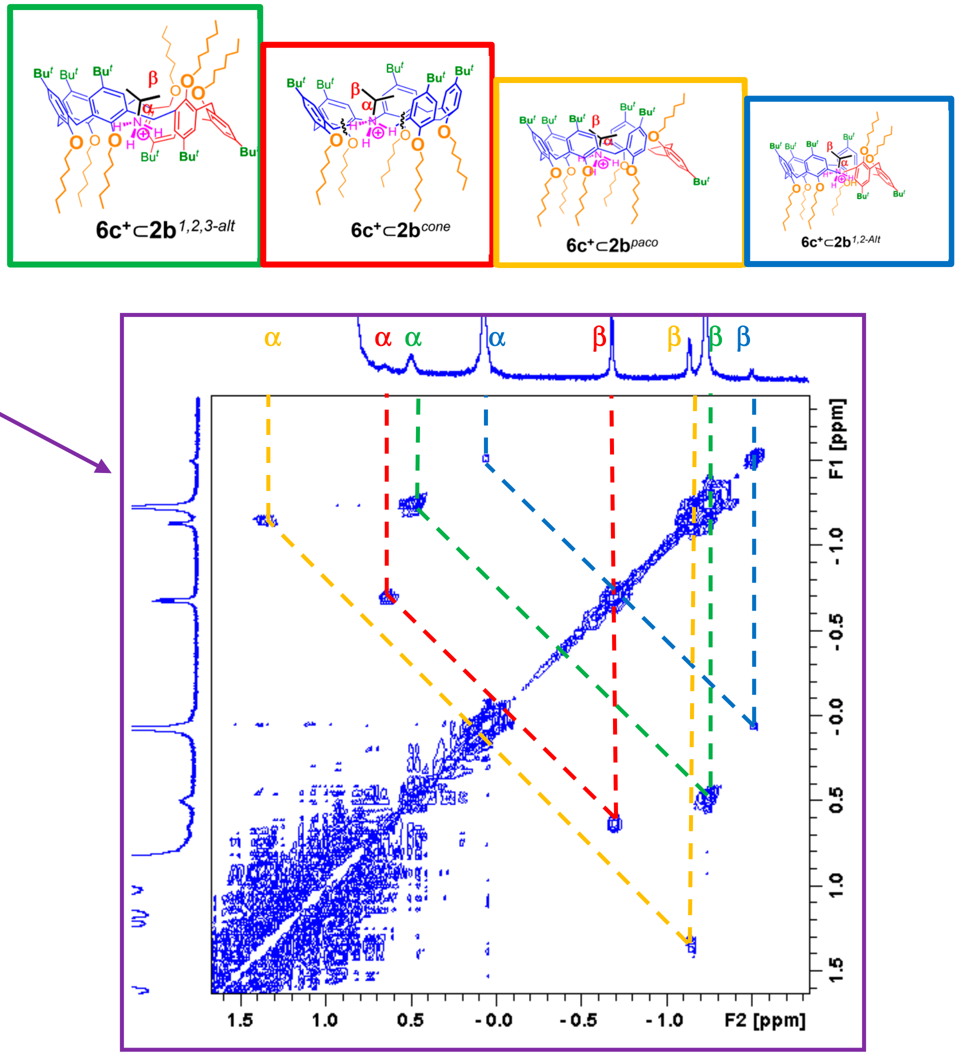

Figure S13. 2D COSY spectrum of **6c$^+$⊂2b** (600 MHz, $CDCl_3$, 298 K).

2D HSQC spectrum of 6c$^+$⊂2b

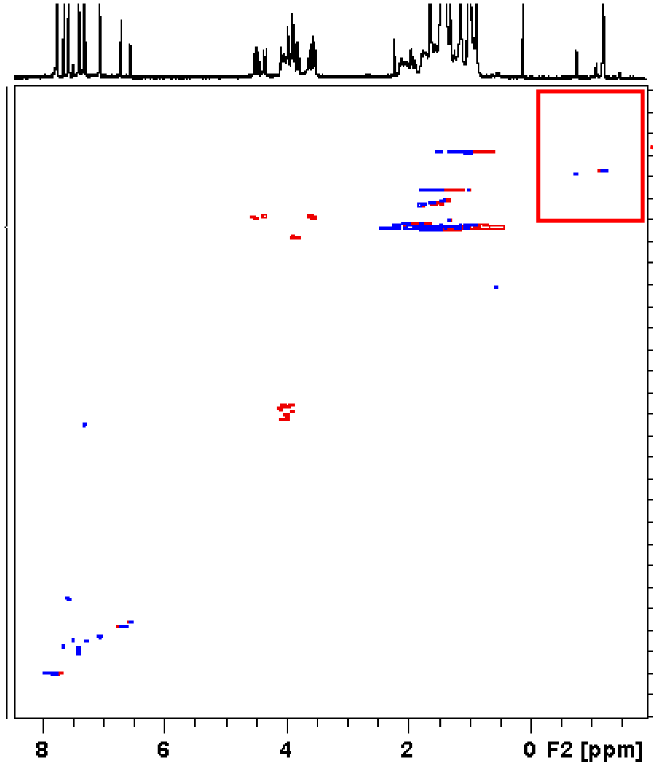

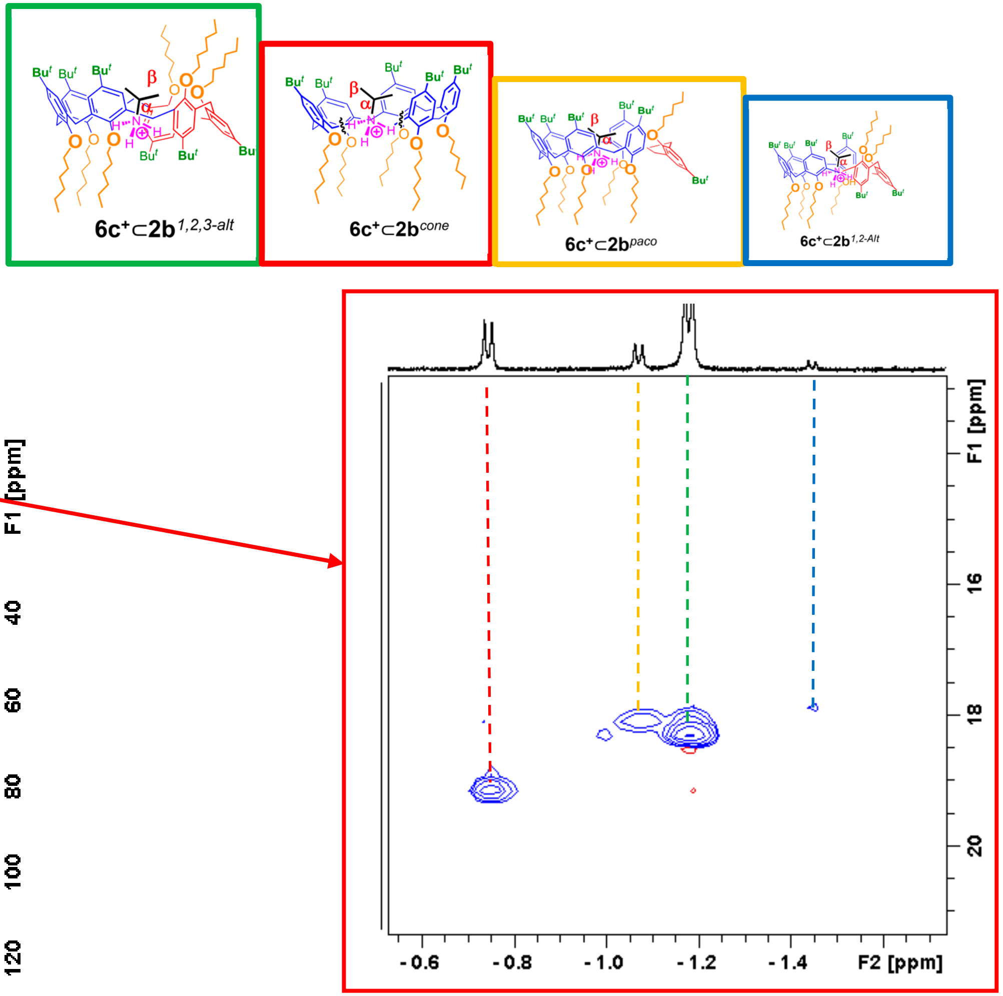

Figure S15. 2D HSQC spectrum of spectrum of **6c$^+$@2b** (600 MHz, $CDCl_3$, 298 K).

Figure S14. 2D HSQC spectrum of spectrum of **6c$^+$⊂2b** (600 MHz, $CDCl_3$, 298 K).

^{1}H NMR determination of K_{ass} values

The association constant values for the formation of complexes were calculated by means of two methods:

a) ^{1}H NMR competition experiments. In this case, was performed an analysis of a 1:1:1 mixture of host, and two guests (or two host and one guest) in an NMR tube using 0.5 mL of $CDCl_3$ as solvent. The following equation was used to obtain K_{ass} value.

$$K_{A\subset H} = \frac{[HG_A]}{[H][G_A]} \text{ and } K_{B\subset H} = \frac{[HG_B]}{[H][G_B]} \rightarrow K_{rel} = \frac{K_{A\subset H}}{K_{B\subset H}} = \frac{[HG_A][H][G_B]}{[HG_B][H][G_A]} \rightarrow$$

$$\begin{matrix}[HG_A]=[G_B]\\ [HG_B]=[G_A]\end{matrix} \rightarrow K_{rel} = \frac{K_{A\subset H}}{K_{B\subset H}} = \frac{[HG_A]^2}{[HG_B]^2}$$

b) Quantitative ^{1}H NMR experiments using TCE as the internal standard. In this case, ^{1}H NMR experiments were carried out on a 1:1 mixture of host and guest in 0.5 mL of $CDCl_3$ containing 1 µL of 1,1,2,2-tetrachloroethane (d= 1.586 g/mL) as internal standard. The following equation was used to obtain the moles of the complex:

$$\frac{G_a}{G_b} = \frac{F_a}{F_b} \times \frac{N_b}{N_a} \times \frac{M_a}{M_b}$$

Where:

G_a = grams of 1,1,2,2-Tetrachloroethane; G_b= grams of pseudorotaxane.

F_a and F_b = areas of the signal related of 1,1,2,2-tetrachloroethane and pseudorotaxane.

N_a and N_b = numbers of nuclei which cause the signals (N_a for 1,1,2,2-tetrachloroethane; N_b for pseudorotaxane)

M_a and M_b = molecular masses of 1,1,2,2-tetrachloroethane (a) and pseudorotaxane (b).

Table S2. K_{app} values for the formation of the pseudorotaxanes.

	**$6a^{+}\subset 2b$**	**$6b^{+}\subset 2b$**	**$6c^{+}\subset 2b$**
**K_{app}**	**5.2±0.2 x 10^{6} M^{-1}**	**5.6±0.2 x 10^{3} M^{-1}**	**4.7±0.2 x 10^{4} M^{-1}**
**1,2,3-Alt**		2.6±0.2 x 10^{3} M^{-1}	3.1±0.2 x 10^{4} M^{-1}
Cone	5.2±0.2 x 10^{6} M^{-1}	1.8±0.2 x 10^{3} M^{-1}	9.1±0.2 x 10^{3} M^{-1}
**PaCo**		8.1±0.2 x 10^{2} M^{-1}	6.1±0.2 x 10^{3} M^{-1}
**1,2-Alt**		4.3±0.2 x 10^{2} M^{-1}	1.2±0.2 x 10^{3} M^{-1}

Determination of K_{ass} values for complex $6a^+ \subset 2b^{cone}$

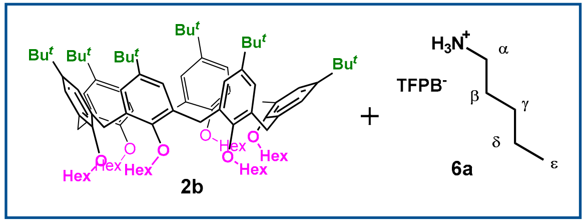

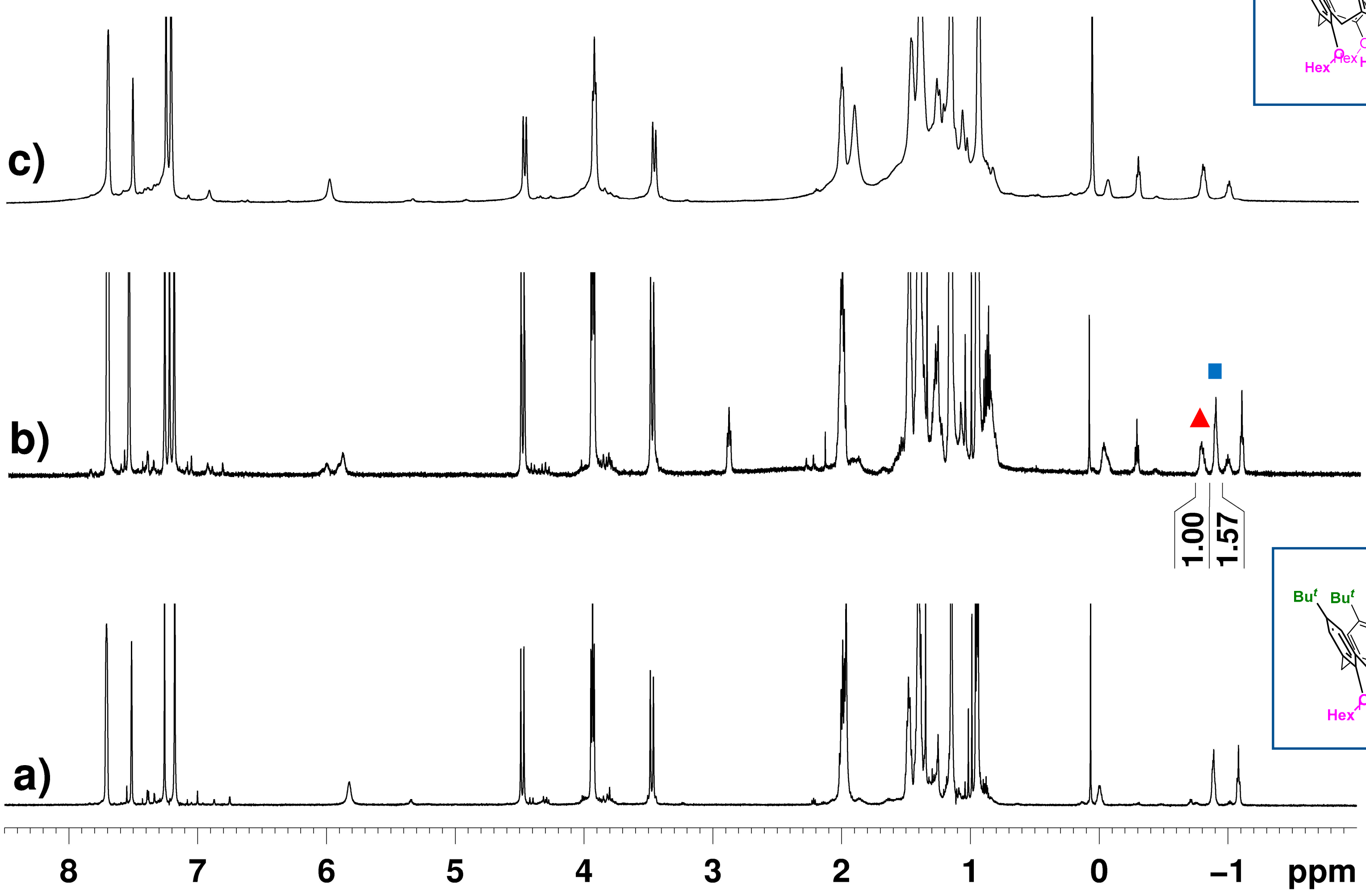

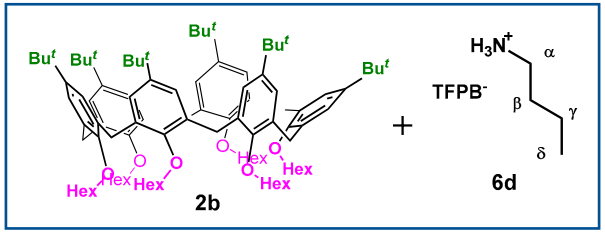

Figure S16. ^{1}H NMR spectrum of **(a)** an equimolar solution (3.9 mM) of **6d**$^+$ and **2b** in 0.4 mL of $CDCl_3$;[1] **(b)** an equimolar solution (3.9 mM) of **6d**$^+$, **6a**$^+$ and **2b** in 0.4 mL of $CDCl_3$. **(c)** an equimolar solution (4.5 mM) of **6a**$^+$ and **2b** in 0.4 mL of $CDCl_3$.The association constant K_a value was calculated by integration of signal of complex **6a**$^+ \subset$ **2b** (▲) and complex **6d**$^+ \subset$ **2b** (■) (600 MHz, $CDCl_3$, 298 K).

Determination of K_{ass} values for complexes $6b^+ \subset 2b$

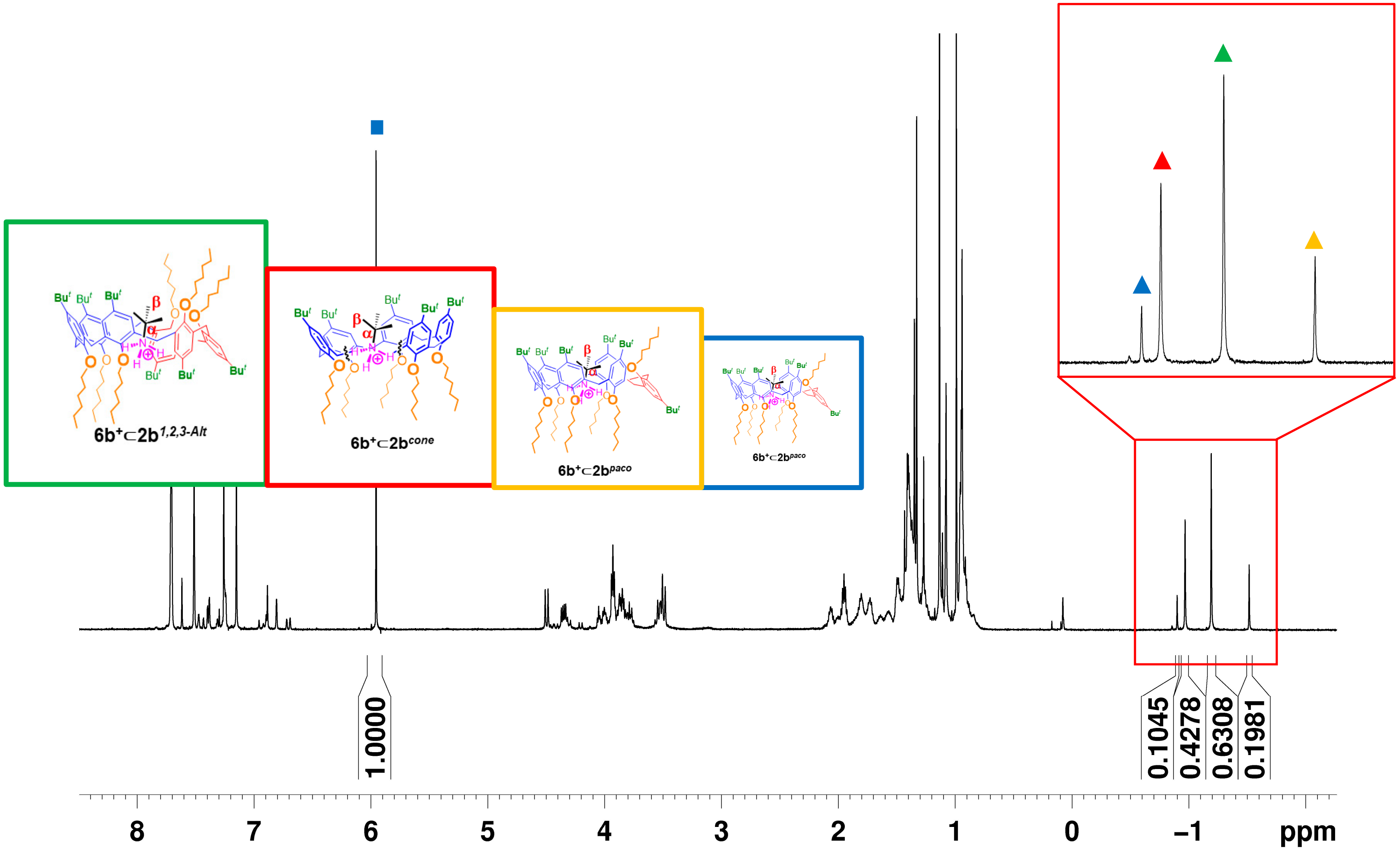

Figure S17. ^{1}H NMR spectrum of an equimolar solution (3.9 mM) of $\mathbf{6b}^+$ and **2b** in 0.4 mL of $CDCl_3$ containing 1 μL of 1,1,2,2-tetrachloroethane. The association constant K_a values were calculated by integration of signals of complexes $\mathbf{6b}^+ \subset \mathbf{2b}$ (▲=1,2,3-Alt; ▲=Cone; ▲=PaCo; ▲=1,2-Alt) and 1,1,2,2-tetrachloroethane (■) (600 MHz, $CDCl_3$, 298 K).

Determination of K_{ass} values for complexes $6c^{+}\subset 2b$

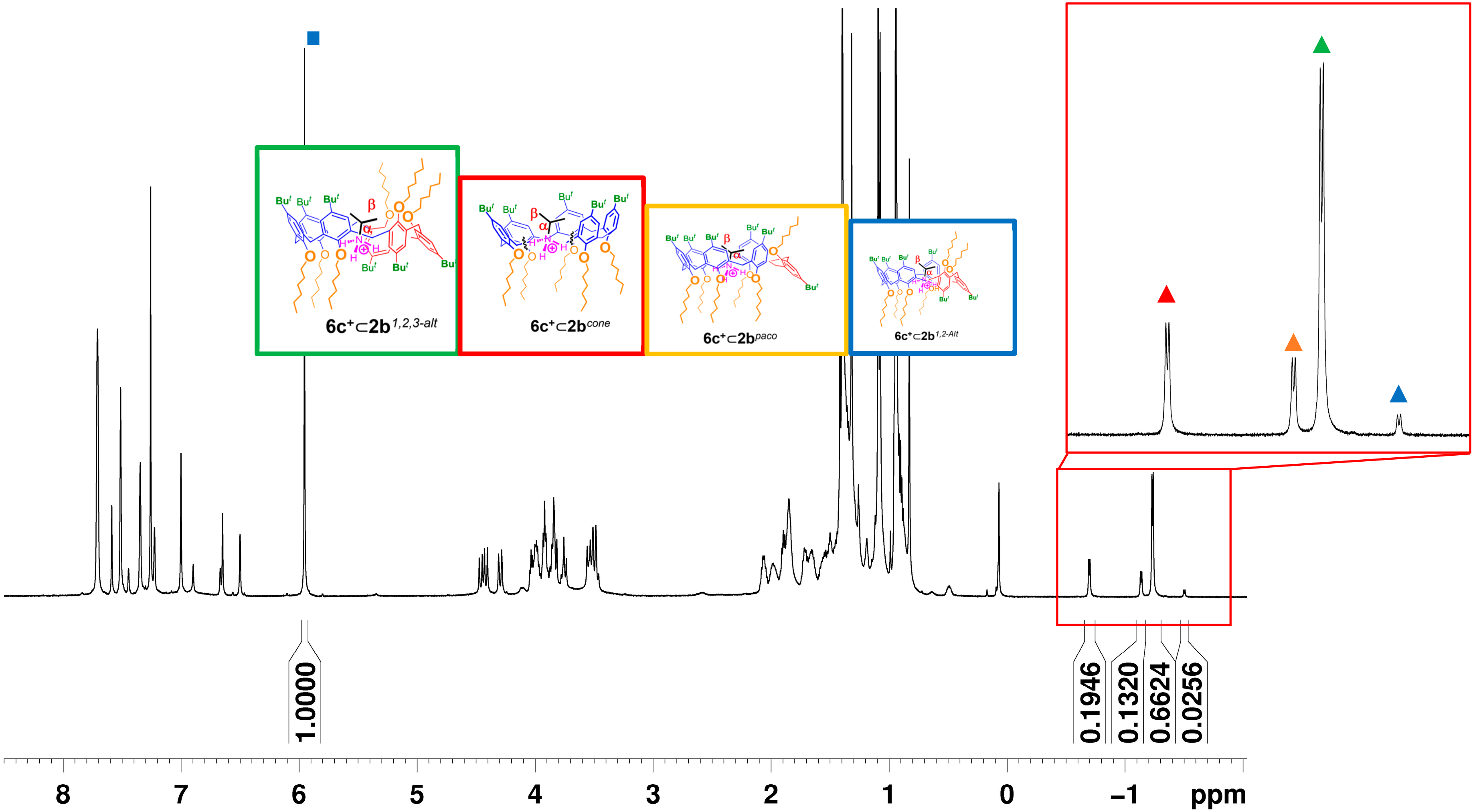

Figure S18. ^{1}H NMR spectrum of an equimolar solution (3.9 mM) of $\mathbf{6c}^{+}$ and **2b** in 0.4 mL of $CDCl_3$ containing 1 μL of 1,1,2,2-tetrachloroethane. The association constant K_a values were calculated by integration of signals of complexes $\mathbf{6c}^{+}\subset\mathbf{2b}$ (▲=1,2,3-Alt; ▲=Cone; ▲=PaCo; ▲=1,2-Alt) and 1,1,2,2-tetrachloroethane (■) (600 MHz, $CDCl_3$, 298 K).

Computational Studies

Computational studies have been performed using the DFT method incorporated in the Gaussian 16 package[2] and using b3lyp/6-31g(d,p) level of theory without solvent. The starting structure for DFT calculations were obtained by molecular dynamics calculation performed by YASARA software.[3] All optimized structures were characterized by 0 imaginary frequency.

Natural bond orbital (NBO) studies were performed with NBO 3.1 version implemented in Gaussian 16 and second-order perturbation theory analysis was performed on optimized structures using the b3lyp/6-31g(d,p) level of theory.[4]

The non-covalent interaction (NCI)[5] investigations were carried out with the Multiwfn program [6] and its plot was graphed with ChemCraft 1.8 program.
Plots for the complexes show RDG values versus electron density multiplied by the sign of the second Hessian eigenvalue and gradient isosurfaces.
The colour scheme was used to help discern the amplitude of the electron density due to various sorts of interactions. Marked in blue-green colors represent medium-strong (H-bond, cation···π, Van der Waals and C-H···π) interactions whereas the red color represents the repulsive ones.

The Expanding Coefficient (EC)[7] was calculated using the follow equation:

$$\mathrm{EC} = \frac{V_{\text{complexed Host}}}{V_{\text{free Host}}}$$

The values of $V_{\text{complexed\_Host}}$ and $V_{\text{free\_Host}}$ were measured with the Caver analyst software[8] employing 1.000.000 of number of samples. In all the cases a probe of 1.0 Å was used.

Table S3. Volumes, EC and log(K_{app}) calculate for the considered complexes

Complex	Volume host complexed	Volume host free	EC	log (K_{app})
$6b^+ \subset 2b^{cone}$	155.7	24.4	6.38	3.26
$6b^+ \subset 2b^{PaCo}$	143	24.4	5.86	2.91
$6b^+ \subset 2b^{1,2\text{-}alt}$	156.3	24.4	6.41	2.63
$6b^+ \subset 2b^{1,2,3\text{-}alt}$	253.1	24.4	10.37	3.41
$6c^+ \subset 2b^{cone}$	155.5	24.4	6.37	3.96
$6c^+ \subset 2b^{PaCo}$	118.2	24.4	4.84	3.79
$6c^+ \subset 2b^{1,2\text{-}alt}$	147.1	24.4	6.03	3.08
$6c^+ \subset 2b^{1,2,3\text{-}alt}$	147.1	24.4	6.03	4.49

$6a^+ \subset 2b^{cone}$

O	0.1466	3.8357	2.2784
O	-2.6735	3.6816	-0.0899
O	2.7318	-0.1595	1.0635
O	3.3798	-3.517	-0.8474
O	0.3273	-2.5145	-2.4355
O	-2.2426	0.6908	-2.1513
C	-3.7207	3.0266	0.5448
C	-1.4084	-5.6763	-1.7434
H	-1.163	-6.6618	-1.3579
C	-4.6867	2.3556	-0.2199
C	-2.1135	1.6342	4.1689
H	-3.1633	1.5866	4.4369
C	-0.3762	-4.744	-1.8624
C	-5.7801	1.775	0.4376
H	-6.5195	1.2642	-0.1682
C	-0.6857	-3.4804	-2.3952
C	-3.8112	3.066	1.9492
C	2.5824	-3.9161	0.2143
C	2.8994	0.6702	3.325
C	-4.9427	2.5105	2.5517
H	-5.0453	2.6071	3.6288
C	-4.6306	0.9555	-2.3972
C	0.5766	1.7754	3.4457
C	0.6685	-5.1082	1.0659
H	-0.2101	-5.7147	0.8688
C	-4.5873	2.3271	-1.7411
H	-5.4164	2.9156	-2.153
H	-3.6679	2.839	-2.0205
C	-1.6979	2.6718	3.3277
C	-0.3303	2.7629	3.0173
C	-1.2309	0.6817	4.6904
C	-3.4636	0.193	-2.5984
C	0.1067	0.7503	4.2749
H	0.8172	0.0126	4.6238
C	1.43	-4.6896	-0.0295
C	3.4034	0.5145	4.6268
H	3.1831	1.2997	5.3414
C	-1.9838	-3.1535	-2.82
C	1.0162	-4.8047	2.3922
C	1.0415	-5.1047	-1.4445
H	1.7597	-4.6651	-2.1359
C	-5.9548	1.8672	1.8217
C	3.1953	-0.3195	2.3781
C	2.914	-3.5138	1.5206
C	-3.5121	-1.0493	-3.2517
C	-2.9864	-4.1192	-2.6474
H	-3.9929	-3.8624	-2.9457
C	-2.7064	3.6874	2.7924
H	-2.1657	4.4248	2.2017
C	3.9064	-1.483	2.7372
C	-2.2334	-1.8233	-3.5322
H	-1.3878	-1.176	-3.3102
H	-2.1983	-2.0279	-4.6119
C	2.1244	-3.9702	2.5853
H	2.4094	-3.6699	3.5869
C	-7.2116	1.3442	2.5442
C	4.3789	-1.5835	4.0475
H	4.9427	-2.4734	4.3126
C	-4.7522	-1.5002	-3.7316
H	-4.7725	-2.4234	-4.2982
C	-2.7312	-5.3857	-2.1108
C	-5.9367	-0.7817	-3.5473
C	4.1594	-0.592	5.0174
C	-5.8407	0.4438	-2.8703
H	-6.7333	1.0475	-2.7326
C	-1.7342	-0.3519	5.717
C	-7.3015	-1.2628	-4.0753
C	-3.8271	-6.4539	-1.9351
C	-2.8904	-1.1868	5.1212
H	-3.7359	-0.5619	4.8201
H	-3.2593	-1.9079	5.8588
H	-2.5589	-1.7468	4.2402
C	4.4718	-4.4185	-1.1384
H	5.0959	-4.5365	-0.2423
H	4.0678	-5.4079	-1.3928
C	-2.247	0.3972	6.9712
H	-1.4501	0.9965	7.4227
H	-2.6037	-0.3158	7.7227
H	-3.075	1.0703	6.7308
C	-5.2127	-5.952	-2.383
H	-5.9551	-6.7414	-2.2323
H	-5.2275	-5.688	-3.4455
H	-5.5383	-5.08	-1.8061
C	-2.9819	5.0535	-0.4034
C	-1.8237	5.6657	-1.1747
H	-3.9072	5.0964	-0.9951
C	-7.9795	2.5471	3.1443
H	-7.3673	3.1008	3.8625
H	-8.8795	2.2043	3.6666
H	-8.2875	3.2457	2.3599
C	-8.1632	0.5931	1.5937
H	-8.5391	1.242	0.7963
H	-9.0301	0.2279	2.153
H	-7.6797	-0.2732	1.1298
C	-0.622	-1.3189	6.1621
H	-1.0229	-2.0388	6.8822
H	0.2064	-0.7928	6.6472
H	-0.2168	-1.8855	5.3174
C	0.2168	-5.4224	3.5561
C	-6.8135	0.3798	3.6857
H	-6.265	-0.4856	3.2997

H	-7.7076	0.0108	4.1997
H	-6.1852	0.8694	4.4358
C	-3.4692	-7.7003	-2.7795
H	-2.5133	-8.1389	-2.4784
H	-3.4017	-7.4482	-3.8426
H	-4.2384	-8.471	-2.6622
C	-3.92	-6.8549	-0.4434
H	-2.9787	-7.2724	-0.0731
H	-4.6955	-7.6151	-0.3022
H	-4.175	-5.9916	0.1802
C	-8.2823	-1.4208	-2.8891
H	-8.4311	-0.4783	-2.354
H	-7.9129	-2.1593	-2.1699
H	-9.2614	-1.7566	-3.2471
C	-7.866	-0.2222	-5.0717
H	-8.0072	0.7569	-4.6049
H	-8.8389	-0.5505	-5.453
H	-7.1937	-0.0918	-5.9258
C	0.8027	-5.0474	4.93
H	0.2124	-5.5187	5.7216
H	0.7838	-3.9679	5.1077
H	1.8352	-5.3944	5.0388
C	-1.2535	-4.948	3.5019
H	-1.7335	-5.2099	2.5539
H	-1.3216	-3.8624	3.6291
H	-1.8334	-5.4137	4.3056
C	-7.2058	-2.6175	-4.8019
H	-8.1981	-2.9172	-5.1524
H	-6.8384	-3.4108	-4.1423
H	-6.5513	-2.5669	-5.6783
C	0.2563	-6.9652	3.4286
H	-0.2	-7.3122	2.4971
H	-0.2916	-7.4257	4.2573
H	1.286	-7.3354	3.4561
H	1.1474	-6.1929	-1.5265
C	4.7358	-0.7591	6.4351
C	4.1665	-2.0476	7.0752
C	6.2772	-0.8667	6.3505
C	4.3855	0.4284	7.3513
H	4.8191	0.2673	8.3427
H	4.7857	1.3724	6.9677
H	3.304	0.5408	7.4809
H	4.5711	-2.1801	8.0841
H	3.0752	-1.9992	7.1526
H	4.4231	-2.9405	6.4973
H	6.7037	-0.9899	7.3516
H	6.5952	-1.7224	5.7476
H	6.7101	0.0355	5.9067
H	-3.1648	4.2105	3.6404
H	-3.1659	5.6105	0.5265
C	5.2819	-3.8482	-2.2912

C	-1.5503	1.5093	-3.1145
H	-1.2969	0.904	-3.9969
H	-2.2096	2.3173	-3.4568
C	-0.2979	2.0787	-2.4676
C	3.7255	0.2257	0.0713
C	4.276	1.6379	0.2298
H	4.5489	-0.4944	0.093
H	3.2108	0.1118	-0.8869
C	4.1375	-2.6395	1.7768
H	4.9327	-3.2664	2.1978
H	4.506	-2.2909	0.8125
C	2.039	1.876	3.0045
H	2.4911	2.7556	3.4797
H	2.0538	2.0818	1.9338
C	-0.9376	-0.8056	0.7161
N	0.3713	-1.464	0.3114
H	0.6395	-2.2734	1.0063
C	-2.0733	-1.8455	0.7223
H	0.303	-1.8891	-0.7008
C	0.5226	2.9256	-3.45
H	-0.5942	2.6713	-1.5949
H	0.3167	1.2464	-2.0965
C	1.7915	3.5189	-2.8235
H	0.8034	2.3109	-4.3179
H	-0.1077	3.7332	-3.8435
C	2.7071	4.2587	-3.8125
H	1.5068	4.1997	-2.0092
H	2.3652	2.7071	-2.3539
C	2.0839	5.5033	-4.4541
H	3.6242	4.5511	-3.2852
H	3.0222	3.5614	-4.6016
H	2.8069	6.0168	-5.0954
H	1.2177	5.254	-5.0753
H	1.7514	6.2168	-3.6916
C	-2.0651	7.1461	-1.5001
H	-0.9072	5.5478	-0.5834
H	-1.6751	5.0995	-2.1031
C	-0.9376	7.7835	-2.3209
H	-3.0116	7.2495	-2.0491
H	-2.195	7.7092	-0.5654
C	-1.1751	9.2645	-2.6405
H	0.0118	7.6791	-1.7758
H	-0.8111	7.2256	-3.2596
C	-0.0434	9.8948	-3.4571
H	-2.1225	9.3683	-3.1862
H	-1.3038	9.8204	-1.7021
H	-0.2434	10.9496	-3.6688
H	0.9111	9.84	-2.9209
H	0.087	9.3825	-4.417
C	6.476	-4.7355	-2.6684
H	4.6227	-3.7218	-3.1594

H	5.6333	-2.8457	-2.0149
C	7.2996	-4.1711	-3.8327
H	7.1274	-4.8646	-1.7929
H	6.1171	-5.74	-2.9318
C	8.4948	-5.0512	-4.2189
H	6.6478	-4.0403	-4.7083
H	7.6592	-3.1662	-3.5693
C	9.3114	-4.4809	-5.382
H	9.145	-5.1816	-3.3434
H	8.1343	-6.055	-4.4811
H	10.155	-5.1311	-5.6326
H	8.6971	-4.3724	-6.2829
H	9.7157	-3.4925	-5.1364
C	5.3923	1.9079	-0.7915
H	3.47	2.3706	0.106
H	4.6727	1.7617	1.2443
C	6.0252	3.2974	-0.6497
H	6.1757	1.1446	-0.6814
H	4.9963	1.7918	-1.8103
C	7.1447	3.5603	-1.6647
H	5.2477	4.0659	-0.7578
H	6.4241	3.4107	0.3682
C	7.7816	4.9449	-1.5156
H	7.9186	2.7883	-1.5566
H	6.7445	3.4478	-2.6816
H	8.5751	5.1002	-2.253
H	7.0401	5.7399	-1.6533
H	8.2229	5.0736	-0.5211
C	0.4774	4.9763	3.098
C	0.8897	6.144	2.2163
H	-0.3944	5.249	3.7072
H	1.2898	4.7084	3.7894
C	2.1605	5.9123	1.3909
H	0.0543	6.4	1.5522
H	1.0347	7.0092	2.8773
C	2.5984	7.1613	0.6159
H	2.9742	5.5969	2.0597
H	1.9929	5.0799	0.6958
C	3.8557	6.9543	-0.2362
H	1.775	7.4951	-0.0318
H	2.7744	7.983	1.3246
C	4.2917	8.2159	-0.9871
H	4.6762	6.6092	0.4075
H	3.6727	6.1444	-0.9565
H	5.1903	8.0362	-1.586
H	3.5055	8.5657	-1.6658
H	4.5148	9.0329	-0.2922
C	1.062	-2.4314	-3.6813
C	2.1501	-1.3801	-3.5447
H	1.4926	-3.4148	-3.9062
H	0.3663	-2.1782	-4.4901
C	2.9891	-1.2425	-4.823
H	1.6833	-0.4153	-3.305
H	2.7915	-1.6559	-2.6995
C	4.0763	-0.1658	-4.7187
H	3.4581	-2.2082	-5.0574
H	2.3312	-1.0107	-5.6719
C	4.9299	-0.0331	-5.986
H	3.606	0.8031	-4.4964
H	4.7298	-0.391	-3.8637
C	6.0021	1.0549	-5.8788
H	5.4069	-0.9984	-6.2022
H	4.2753	0.1801	-6.8416
H	6.5941	1.1228	-6.7965
H	5.5527	2.0386	-5.7009
H	6.6929	0.8523	-5.0526
H	-1.8507	-2.6393	1.4395
H	-2.1541	-2.329	-0.2542
H	1.1926	-0.7322	0.3157
C	-3.4347	-1.2226	1.0822
H	-0.7622	-0.3693	1.7035
H	-1.1021	-0.0031	-0.008
C	-4.5678	-2.263	1.1381
H	-3.3709	-0.7171	2.0485
H	-3.6949	-0.4499	0.3554
H	-4.6393	-2.7915	0.1858
H	-4.3389	-3.0226	1.8881
C	-5.9325	-1.6366	1.4627
H	-6.7138	-2.398	1.5047
H	-5.9244	-1.1285	2.4282
H	-6.2312	-0.9089	0.7068

________________________--

Energy = -4686.064584 a.u.

0 imaginary frequency

$6b^{+}\subset 2b^{1,2,3\text{-}Alt}$

C	9.8294	13.8476	4.9648
N	8.4431	13.9114	5.4439
H	8.2098	14.866	5.6911
C	9.9053	14.4915	3.5775
C	10.2952	12.3926	4.8971
C	10.7402	14.5944	5.9384
H	7.8037	13.5598	4.743
H	9.5913	15.5359	3.5889
H	9.2871	13.9705	2.8447
H	8.3375	13.3425	6.2787
H	11.3518	12.3435	4.6365
H	10.1705	11.8722	5.8432
H	9.7538	11.831	4.1449

H	10.9285	14.4659	3.2011
H	11.7765	14.5562	5.6015
H	10.4668	15.643	6.0015
H	10.7072	14.1858	6.947
O	8.0977	12.2858	7.7044
O	7.6221	10.2808	4.8684
O	8.2222	16.2928	6.9889
O	9.6271	18.8104	3.8051
O	10.2867	15.3771	0.5895
O	10.8657	11.8228	1.7689
C	6.8965	11.2811	4.2786
C	13.7091	16.526	1.3375
H	14.1682	17.3843	1.8025
C	5.9213	11.9824	5.0323
C	6.0212	14.9523	9.2001
H	6.1649	15.79	9.8657
C	12.3036	16.4209	1.4024
C	5.1984	13.0342	4.4346
H	4.4358	13.5257	5.0186
C	11.6507	15.3633	0.7141
C	7.1485	11.6401	2.931
C	10.6976	18.2413	4.4538
C	8.9411	16.0459	9.2958
C	6.4187	12.7107	2.3698
H	6.5768	12.9861	1.3416
C	10.4168	12.6657	-0.4585
C	5.7536	12.7602	7.4474
C	12.8114	17.0285	4.347
H	13.557	16.5078	3.7783
C	7.1557	14.1961	8.8431
C	7.0119	13.0666	8.0062
C	4.7447	14.6434	8.6719
C	9.9947	12.0076	0.7259
C	4.633	13.5432	7.7918
H	3.6703	13.294	7.3699
C	11.6824	17.5713	3.686
C	9.5057	16.6225	10.454
H	9.5332	16.0305	11.355
C	12.4219	14.3361	0.1117
C	13.0028	17.1741	5.74
C	11.5197	17.5011	2.1563
H	10.4584	17.4021	1.9334
C	5.4358	13.4129	3.0968
C	8.876	16.8003	8.0925
C	10.8178	18.3013	5.8672
C	8.6697	11.5094	0.814
C	13.8297	14.4294	0.1438
H	14.4115	13.6511	-0.3261
C	9.505	18.0767	8.0482
C	11.9925	17.818	6.4815
H	12.1094	17.9196	7.5495
C	4.6196	14.5105	2.3917
C	10.0514	18.6298	9.2241
H	10.5138	19.6045	9.1785
C	7.8189	11.5941	-0.3085
H	6.8379	11.1535	-0.2292
C	14.496	15.5285	0.724
C	8.2139	12.2526	-1.4922
C	10.0465	17.9266	10.4451
C	9.5211	12.784	-1.5432
H	9.8738	13.2618	-2.4425
C	3.4925	15.4755	9.0035
C	7.269	12.3389	-2.7076
C	16.0338	15.6012	0.6777
C	3.7847	16.6454	9.9685
H	4.1733	16.2924	10.9246
H	2.88	17.2142	10.1863
H	4.5057	17.3474	9.5476
C	9.5515	20.2325	3.8933
H	8.5629	20.5134	4.2578
H	10.2712	20.6408	4.6053
C	2.4343	14.5724	9.6676
H	2.1121	13.7624	9.0135
H	1.5412	15.1372	9.9375
H	2.822	14.1192	10.5808
C	16.5207	15.4948	-0.7822
H	17.6057	15.5827	-0.8472
H	16.0916	16.2868	-1.3972
H	16.2547	14.5437	-1.2423
C	7.0352	8.9925	4.7436
C	7.7565	8.0425	5.7057
H	5.9695	9.0238	4.9781
C	5.5694	15.5079	1.7029
H	6.2887	15.9316	2.4019
H	5.0199	16.3303	1.2449
H	6.14	15.0423	0.9036
C	3.7246	13.8589	1.3207
H	3.0368	13.1385	1.7653
H	4.3085	13.3271	0.5694
H	3.1254	14.6026	0.7939
C	2.9139	16.0742	7.7049
H	2.6065	15.306	6.9956
H	2.0348	16.6869	7.9084
H	3.6439	16.7098	7.2029
C	14.2675	16.6678	6.463
C	3.705	15.2981	3.3559
H	2.9399	14.6607	3.8005
H	3.1761	16.0962	2.8332
H	4.2652	15.764	4.1656
C	16.5924	16.9199	1.2562
H	16.338	17.0411	2.3096
H	16.2157	17.7881	0.7139

H	17.6805	16.951	1.1911
C	16.6237	14.441	1.5007
H	16.3156	14.5086	2.5439
H	17.7139	14.4517	1.4824
H	16.3044	13.468	1.1272
C	7.4947	11.0989	-3.589
H	7.2896	10.1793	-3.0398
H	8.5261	11.044	-3.9392
H	6.849	11.1083	-4.4678
C	5.7837	12.3819	-2.2824
H	5.4499	11.4484	-1.8307
H	5.1302	12.5513	-3.1388
H	5.599	13.1847	-1.5695
C	15.2314	15.9016	5.5304
H	16.1071	15.5427	6.0723
H	15.6005	16.5327	4.7212
H	14.7516	15.0285	5.0868
C	15.0295	17.8761	7.0402
H	14.4319	18.4266	7.7671
H	15.3096	18.5764	6.2524
H	15.9455	17.5676	7.5452
C	7.5325	13.6037	-3.5543
H	6.7907	13.7152	-4.3458
H	8.5015	13.5812	-4.0517
H	7.4915	14.5027	-2.941
C	13.8885	15.715	7.6169
H	13.257	16.1936	8.3651
H	14.7761	15.3567	8.1395
H	13.3578	14.8369	7.2526
H	11.8152	18.4658	1.7461
C	10.6667	18.5759	11.6965
C	9.9527	19.9099	11.9948
C	10.5482	17.6921	12.9578
C	12.1649	18.841	11.4476
H	12.6426	19.2813	12.3235
H	12.6945	17.9161	11.2161
H	12.3298	19.5265	10.6165
H	10.3517	20.3835	12.8925
H	10.0613	20.6286	11.1829
H	8.8846	19.7564	12.1536
H	10.9771	18.1879	13.8294
H	9.5075	17.4712	13.1984
H	11.0794	16.7467	12.8419
H	7.1313	8.6355	3.7179
C	9.8115	20.8657	2.5208
C	11.6442	10.6346	1.6577
H	11.0064	9.7601	1.791
H	12.0899	10.5366	0.6687
C	12.7476	10.6343	2.7239
C	7.0065	16.9863	6.6929
C	6.8566	17.2131	5.1846
H	6.1557	16.4035	7.0346
H	6.9363	17.9368	7.2264
C	9.7001	18.8602	6.7481
H	9.9472	19.8964	6.9794
H	8.7655	18.9074	6.1999
C	13.6313	11.8935	2.6699
H	12.2984	10.5332	3.7106
H	13.3629	9.7452	2.586
C	14.704	11.9508	3.7684
H	14.1017	11.9738	1.6897
H	12.9999	12.7782	2.7653
C	15.858	10.9603	3.5491
H	15.1109	12.9612	3.7932
H	14.248	11.7909	4.7463
C	16.9969	11.1532	4.5569
H	15.4896	9.9363	3.6166
H	16.2563	11.079	2.5404
H	17.7998	10.4375	4.3776
H	17.4239	12.1539	4.4832
H	16.6507	11.0118	5.5803
C	7.2155	6.6069	5.6392
H	8.8231	8.0422	5.4773
H	7.6578	8.4203	6.7237
C	7.9392	5.6605	6.612
H	6.1478	6.6175	5.8598
H	7.3164	6.2278	4.6212
C	7.4699	4.1989	6.5166
H	9.0098	5.6923	6.4041
H	7.8216	6.0166	7.6365
C	6.0341	3.976	7.0124
H	7.5651	3.8433	5.4897
H	8.1409	3.5776	7.1115
H	5.7783	2.9163	6.9885
H	5.9118	4.3211	8.0395
H	5.3066	4.4993	6.3921
C	8.6741	20.621	1.5189
H	9.9337	21.9412	2.6546
H	10.758	20.5041	2.1207
C	8.9496	21.2606	0.1499
H	8.5124	19.5491	1.3952
H	7.7495	21.0275	1.9301
C	7.7281	21.2141	-0.7785
H	9.2526	22.3003	0.2829
H	9.7926	20.7524	-0.3206
C	8.0336	21.7457	-2.1832
H	7.3602	20.1921	-0.8538
H	6.9161	21.798	-0.343
H	7.1467	21.7035	-2.8161
H	8.3676	22.7833	-2.1496
H	8.8136	21.1575	-2.6677
C	5.965	18.4306	4.8854

H	7.8343	17.3697	4.7334
H	6.4526	16.3147	4.721
C	5.7305	18.6621	3.388
H	5.0025	18.3161	5.3864
H	6.4273	19.3212	5.3138
C	5.0216	19.9899	3.0917
H	6.6803	18.6288	2.8521
H	5.1185	17.8522	2.9997
C	4.6997	20.1545	1.6017
H	4.0959	20.0514	3.6655
H	5.6449	20.822	3.4221
H	4.2255	21.1171	1.4085
H	5.6019	20.101	0.9933
H	4.0201	19.375	1.2552
C	8.3029	11.2498	8.6643
C	9.545	10.4342	8.2914
H	8.413	11.6623	9.6688
H	7.4347	10.5902	8.6932
C	10.8565	11.1992	8.5258
H	9.5608	9.5213	8.8886
H	9.4675	10.1114	7.2528
C	12.084	10.4283	8.0213
H	10.8133	12.1686	8.0338
H	10.9689	11.408	9.5908
C	13.3878	11.2185	8.199
H	12.1582	9.4779	8.5524
H	11.9552	10.1812	6.9663
C	14.6158	10.4268	7.7364
H	13.3321	12.154	7.6405
H	13.5138	11.4922	9.2476
H	15.5263	11.0131	7.8632
H	14.733	9.507	8.3105
H	14.5391	10.1554	6.6831
C	9.8609	16.1465	-0.5276
C	8.3403	16.3089	-0.4856
H	10.3338	17.1302	-0.5164
H	10.163	15.6581	-1.455
C	7.8239	17.2485	-1.5885
H	7.8815	15.3246	-0.5766
H	8.0567	16.7037	0.4903
C	6.3196	17.5565	-1.4931
H	8.3715	18.1891	-1.5215
H	8.0603	16.841	-2.5721
C	5.4183	16.3873	-1.9172
H	6.0739	17.8743	-0.4788
H	6.0993	18.4112	-2.1346
C	3.9272	16.7183	-1.79
H	5.636	16.1131	-2.9501
H	5.638	15.5079	-1.315
H	3.3139	15.8692	-2.0932
H	3.6618	16.9677	-0.7623
H	3.6554	17.5652	-2.4208
C	11.8525	13.1516	-0.674
H	11.9793	13.4062	-1.7261
H	12.5123	12.3003	-0.5236
C	8.1339	10.8265	2.0745
H	7.6109	9.9252	1.7546
H	8.957	10.4636	2.6844
C	8.5217	14.5792	9.3934
H	8.5371	14.2581	10.4353
H	9.3076	14.0065	8.9049
C	5.5894	11.6007	6.4721
H	4.5579	11.2474	6.5012
H	6.1886	10.7553	6.8031

________________________--

Energy = -4646.726064 a.u.

0 imaginary frequency

$6b^{+}\subset 2b^{cone}$

O	5.671	14.675	2.3808
O	5.3162	15.4086	5.5712
O	5.7715	10.2004	1.2607
O	5.9374	6.5727	2.4744
O	5.3021	7.6382	5.4399
O	4.6096	11.6039	6.8161
C	6.5408	15.0434	6.0698
C	8.628	6.904	6.8662
H	9.5076	6.3478	6.5827
C	6.6347	14.3588	7.3058
C	9.3481	15.0004	2.4344
H	10.1053	15.5156	3.0051
C	7.5108	6.8638	6.0088
C	7.9008	13.9694	7.7935
H	7.9418	13.4602	8.7427
C	6.3346	7.5779	6.3433
C	7.7115	15.3104	5.3232
C	7.2572	6.932	2.4136
C	6.6704	11.639	-0.5041
C	8.9651	14.9081	5.8283
H	9.8525	15.1276	5.2563
C	5.3627	12.7111	8.8205
C	7.3453	13.6686	0.9625
C	9.4322	7.1684	3.4989
H	10.0598	6.9586	4.3508
C	5.3816	14.0681	8.1268
H	5.2774	14.8576	8.8709
H	4.5062	14.1513	7.4941
C	7.9956	15.1623	2.7941
C	6.99	14.5162	2.0399
C	9.7288	14.1788	1.3522
C	5.0156	11.5294	8.1251

C	8.7109	13.5195	0.6254
H	8.9761	12.8831	-0.1999
C	8.0953	6.7223	3.5309
C	7.3171	11.7401	-1.7547
H	7.4384	12.726	-2.1761
C	6.2594	8.2736	7.5778
C	9.9562	7.8442	2.374
C	7.5893	5.9912	4.7643
H	6.6224	5.5288	4.5835
C	9.0826	14.2286	7.0607
C	6.4598	10.3593	0.0781
C	7.7626	7.603	1.2771
C	5.1325	10.2778	8.779
C	7.3968	8.2949	8.4162
H	7.3641	8.8253	9.3524
C	7.6478	16.0427	3.9875
H	6.67	16.4954	3.8399
C	7.0077	9.2188	-0.5579
C	4.9609	8.9584	8.0264
H	4.319	9.111	7.165
H	4.4213	8.2623	8.669
C	9.1034	8.041	1.2637
H	9.4704	8.5197	0.3688
C	10.4746	13.8006	7.5612
C	7.6862	9.3509	-1.7863
H	8.097	8.4704	-2.2576
C	5.4339	10.2392	10.1576
H	5.4724	9.2731	10.6358
C	8.5971	7.6403	8.0696
C	5.7309	11.4168	10.8806
C	7.8428	10.6056	-2.4103
C	5.7062	12.6451	10.186
H	5.9602	13.5574	10.7046
C	11.2218	14.0237	1.0086
C	6.1129	11.3971	12.3719
C	9.8092	7.7309	9.0154
C	11.9715	13.4453	2.2257
H	11.9252	14.1073	3.0901
H	13.0274	13.2858	2.0045
H	11.5514	12.4869	2.5297
C	5.6423	5.3097	1.8913
H	6.1736	5.169	0.9482
H	5.9534	4.5082	2.5621
C	11.8128	15.4023	0.6533
H	11.2882	15.8477	-0.1929
H	12.8664	15.3269	0.3821
H	11.7472	16.1061	1.4829
C	10.1926	9.2102	9.2217
H	11.0674	9.3099	9.8652
H	9.3916	9.7842	9.6879
H	10.4289	9.6918	8.2721
C	4.8141	16.6335	6.0846
C	3.3089	16.6757	5.7994
H	4.9944	16.7223	7.1571
C	10.4206	13.0293	8.8979
H	10.0053	13.6401	9.7004
H	11.4156	12.7194	9.2193
H	9.8157	12.1261	8.8153
C	11.1469	12.8807	6.5203
H	10.5589	11.9796	6.3492
H	12.1356	12.5628	6.853
H	11.28	13.3723	5.5569
C	11.4736	13.0851	-0.1927
H	12.539	12.9905	-0.4053
H	11.0021	13.4595	-1.1023
H	11.0966	12.0791	-0.0091
C	11.4331	8.287	2.3317
C	11.3477	15.0526	7.774
H	11.4958	15.612	6.8503
H	12.3366	14.7905	8.1516
H	10.8909	15.7311	8.4954
C	9.4499	7.1006	10.3753
H	9.1569	6.0564	10.2596
H	8.6244	7.6181	10.8641
H	10.2954	7.1288	11.0635
C	11.0559	7.	8.47
H	10.8763	5.9317	8.3442
H	11.9008	7.0973	9.1528
H	11.3754	7.4076	7.5101
C	5.1329	12.2813	13.1689
H	4.1059	11.934	13.0504
H	5.1631	13.323	12.8502
H	5.3615	12.2688	14.2351
C	6.0717	9.9807	12.9866
H	5.077	9.5392	12.915
H	6.3341	10.0014	14.045
H	6.7789	9.3077	12.5001
C	11.6448	9.5096	1.411
H	12.6684	9.8813	1.4709
H	10.985	10.3324	1.683
H	11.473	9.2775	0.3601
C	11.9638	8.6771	3.7307
H	12.0122	7.8294	4.4135
H	11.3524	9.4492	4.1968
H	12.9806	9.0673	3.6705
C	7.5464	11.9395	12.5393
H	7.8609	11.9194	13.5833
H	7.6393	12.9704	12.1981
H	8.2607	11.3432	11.97
C	12.2742	7.1166	1.7949
H	12.184	6.2361	2.4321
H	13.3324	7.3746	1.7402

H	11.9558	6.8255	0.7931
H	8.2606	5.1535	4.9585
C	8.5996	10.7008	-3.7477
C	10.051	10.222	-3.5467
C	7.9063	9.8134	-4.8011
C	8.6533	12.1385	-4.3099
H	9.1836	12.171	-5.2624
H	7.6547	12.5388	-4.489
H	9.1764	12.8178	-3.6356
H	10.6265	10.2957	-4.4701
H	10.5622	10.8232	-2.7936
H	10.1006	9.1827	-3.2222
H	8.4048	9.8808	-5.7687
H	7.9031	8.7608	-4.5193
H	6.8684	10.1154	-4.9467
H	8.3444	16.8806	4.0235
H	5.3202	17.4697	5.6011
C	4.1294	5.2592	1.6566
C	3.2113	11.4113	6.5972
H	3.0988	10.8578	5.6647
H	2.7487	10.8044	7.3777
C	2.4837	12.7567	6.4871
C	4.3561	10.39	1.1651
C	3.6367	9.0478	0.9896
H	4.0186	10.8714	2.0835
H	4.0766	11.0536	0.345
C	6.8874	7.8288	0.0501
H	7.1592	7.0769	-0.6914
H	5.8448	7.636	0.2783
C	6.2495	12.9423	0.1755
H	5.8771	13.6266	-0.5872
H	5.4062	12.7649	0.8357
C	7.391	10.8847	4.2196
N	6.8704	9.5835	3.7845
H	6.4952	9.667	2.8446
C	8.604	11.2567	3.3572
C	7.8202	10.8038	5.6897
C	6.2842	11.9277	4.0448
H	7.6082	8.8918	3.7623
C	1.0188	12.6309	6.0469
H	2.512	13.2569	7.4549
H	3.0194	13.3921	5.78
C	0.3308	14.0014	5.9509
H	0.9672	12.1278	5.0804
H	0.4797	11.9997	6.755
C	-1.1453	13.9026	5.5426
H	0.4044	14.5119	6.9124
H	0.8624	14.6256	5.2307
C	-1.8221	15.2768	5.47
H	-1.2269	13.4091	4.5732
H	-1.6832	13.2752	6.2546

H	-2.8672	15.1815	5.1739
H	-1.7968	15.7815	6.4363
H	-1.3294	15.9235	4.743
C	2.633	17.9572	6.3068
H	3.1449	16.5741	4.7261
H	2.8378	15.8111	6.2665
C	1.1198	17.9632	6.0408
H	2.8136	18.0632	7.3775
H	3.0922	18.8235	5.8285
C	0.4365	19.2428	6.5439
H	0.938	17.8512	4.9708
H	0.6626	17.097	6.5212
C	-1.0736	19.2453	6.2785
H	0.6128	19.3572	7.6143
H	0.8867	20.1126	6.0635
H	-1.5315	20.1656	6.6423
H	-1.2872	19.1684	5.2118
H	-1.5647	18.4102	6.779
C	3.6398	3.9226	1.0851
H	3.6123	5.4609	2.595
H	3.8546	6.06	0.9709
C	2.1253	3.9302	0.8268
H	4.167	3.7081	0.1543
H	3.8903	3.1162	1.7758
C	1.6128	2.5993	0.2595
H	1.5976	4.1549	1.7551
H	1.8794	4.735	0.1324
C	0.1037	2.6222	-0.0107
H	2.1392	2.367	-0.6674
H	1.8416	1.7902	0.9545
H	-0.2349	1.6654	-0.4094
H	-0.4592	2.8184	0.9025
H	-0.1567	3.3935	-0.7362
C	2.1071	9.1617	1.0495
H	3.9105	8.6235	0.0239
H	3.9772	8.3525	1.7567
C	1.4217	7.8038	0.8333
H	1.8042	9.5754	2.0125
H	1.7659	9.8656	0.2887
C	-0.1098	7.8984	0.8306
H	1.7535	7.3746	-0.1134
H	1.7383	7.1091	1.6118
C	-0.775	6.5287	0.6493
H	-0.4561	8.3461	1.763
H	-0.4359	8.5652	0.0312
H	-1.8615	6.6202	0.6501
H	-0.4826	6.0678	-0.2947
H	-0.5007	5.8452	1.4538
C	4.9893	15.6658	1.6248
C	3.5016	15.2994	1.5802
H	5.1253	16.6401	2.0948

H	5.3839	15.7421	0.6098
C	2.6718	16.303	0.766
H	3.3921	14.3035	1.1503
H	3.1151	15.2391	2.5983
C	1.1893	15.9117	0.6748
H	2.7605	17.294	1.2136
H	3.0861	16.3819	-0.2401
C	0.3715	16.9052	-0.164
H	1.1018	14.9146	0.2407
H	0.7669	15.8487	1.679
C	-1.1068	16.5115	-0.2684
H	0.4469	17.9025	0.272
H	0.7945	16.9757	-1.1671
H	-1.661	17.2357	-0.8662
H	-1.2244	15.5347	-0.7384
H	-1.5744	16.4685	0.7159
C	4.2459	6.7164	5.6876
C	2.9584	7.2792	5.0745
H	4.4829	5.7531	5.2361
H	4.1037	6.5406	6.7556
C	1.7608	6.3358	5.2561
H	2.7334	8.2425	5.5324
H	3.1131	7.4709	4.0122
C	0.4484	6.9435	4.741
H	1.9572	5.3956	4.7383
H	1.6495	6.0875	6.3127
C	-0.7439	5.9893	4.8976
H	0.2421	7.8716	5.2763
H	0.5581	7.2128	3.6911
C	-2.0603	6.6106	4.4163
H	-0.5551	5.071	4.3395
H	-0.848	5.698	5.9437
H	-2.8872	5.9097	4.5349
H	-2.3047	7.5091	4.9837
H	-2.0085	6.8844	3.3625
H	8.3602	11.2666	2.2945
H	9.4271	10.5582	3.4973
H	6.1494	9.2475	4.4098
H	8.2244	11.757	6.0275
H	6.9801	10.5798	6.3429
H	8.5859	10.0455	5.86
H	8.9774	12.2501	3.613
H	6.5885	12.8938	4.4422
H	6.0374	12.0809	2.9978
H	5.3619	11.6458	4.545

____________________________--

Energy = -4646.754029 a.u.

0 imaginary frequency

$6b^{+}\subset 2b^{paco}$

O	5.5877	13.7716	8.199
O	7.8392	9.7182	5.2373
O	7.6812	16.7561	7.2285
O	9.718	19.0018	3.8441
O	9.9327	15.8904	1.11
O	8.2631	12.1848	0.3245
C	6.9891	10.6762	4.748
C	13.5708	16.5758	1.0262
H	14.2413	17.3576	1.3462
C	6.1731	11.4211	5.6353
C	8.466	11.4586	8.1201
H	8.6854	10.4812	7.7173
C	12.199	16.726	1.3234
C	5.3144	12.4206	5.1319
H	4.6813	12.9428	5.8339
C	11.2786	15.7358	0.8964
C	6.9525	10.9598	3.3618
C	10.8043	18.4012	4.4275
C	8.4718	16.4445	9.4857
C	6.1062	11.9797	2.8838
H	6.1046	12.1863	1.824
C	9.1512	10.7863	2.0762
C	7.7934	13.9924	9.1515
C	12.9588	17.2732	4.2283
H	13.7413	16.887	3.5986
C	7.1746	11.9807	7.9064
C	6.8279	13.2425	8.4409
C	9.4287	12.1691	8.8743
C	9.3313	11.7724	1.0784
C	9.0784	13.4486	9.363
H	9.7978	14.0153	9.9367
C	11.8352	17.8691	3.6155
C	9.3371	16.814	10.5364
H	9.2483	16.2904	11.4755
C	11.7551	14.5711	0.2495
C	13.0704	17.1827	5.6341
C	11.7494	17.969	2.0907
H	10.7335	18.2193	1.7994
C	5.2629	12.7109	3.7489
C	8.5482	17.1059	8.2367
C	10.8732	18.2879	5.8359
C	10.6081	12.3518	0.8824
C	13.1312	14.4394	-0.0269
H	13.4773	13.5483	-0.5287
C	9.5276	18.1137	8.0428
C	10.8104	13.447	-0.166
H	9.8508	13.8793	-0.442
H	11.1911	12.979	-1.0738
C	12.017	17.6976	6.4193
H	12.0973	17.6194	7.4903

C	4.3229	13.7808	3.1644
C	10.3535	18.4938	9.1208
H	11.0791	19.2788	8.965
C	11.701	11.8943	1.6521
H	12.6671	12.3369	1.4791
C	14.0597	15.4304	0.3572
C	11.5421	10.8957	2.6411
C	10.2864	17.8477	10.373
C	10.253	10.3544	2.8403
H	10.0948	9.6058	3.6024
C	10.809	11.5714	9.2176
C	12.7114	10.4107	3.5165
C	15.5559	15.2196	0.0634
C	10.9756	10.1193	8.718
H	10.2296	9.4543	9.1554
H	11.9537	9.718	8.9864
H	10.8952	10.049	7.6335
C	9.879	20.4012	3.654
H	9.6087	20.9313	4.5678
H	10.9196	20.6516	3.4367
C	10.9958	11.5523	10.7487
H	10.9903	12.5521	11.1819
H	11.9452	11.0943	11.0289
H	10.2038	10.9812	11.2347
C	15.7685	15.058	-1.4551
H	16.8238	14.9309	-1.6984
H	15.411	15.9347	-1.9966
H	15.2435	14.1913	-1.856
C	7.265	8.421	5.3488
C	8.1774	7.5751	6.2441
H	6.2591	8.4634	5.769
C	5.1717	14.9711	2.6877
H	5.7369	15.4146	3.5053
H	4.5504	15.7576	2.2604
H	5.8855	14.669	1.9214
C	3.5211	13.2104	1.9752
H	2.9398	12.3375	2.275
H	4.1531	12.9091	1.1423
H	2.8211	13.9464	1.5776
C	11.9299	12.4168	8.5848
H	12.9147	12.0416	8.8659
H	11.8844	13.4603	8.8974
H	11.8819	12.3896	7.4974
C	14.2895	16.5447	6.3278
C	3.2912	14.2933	4.1891
H	2.6904	13.4799	4.5986
H	2.5964	15.	3.7334
H	3.7689	14.8138	5.0153
C	16.4354	16.3972	0.5381
H	16.3647	16.5453	1.6165
H	16.1578	17.3308	0.0475
H	17.4877	16.2212	0.312
C	16.0434	13.9491	0.789
H	15.887	14.0278	1.8656
H	17.1078	13.7792	0.6239
H	15.5215	13.0544	0.45
C	12.8646	8.882	3.3832
H	13.0396	8.595	2.3455
H	11.9807	8.3444	3.7253
H	13.7061	8.5131	3.9705
C	14.0638	11.0503	3.1306
H	14.3338	10.8283	2.0973
H	14.873	10.6755	3.7583
H	14.0493	12.1345	3.2506
C	15.3559	16.0402	5.3307
H	16.2059	15.5965	5.8502
H	15.7492	16.8509	4.7159
H	14.957	15.2737	4.6647
C	14.9621	17.5856	7.245
H	14.2971	17.9308	8.0363
H	15.2767	18.4627	6.6781
H	15.848	17.1757	7.7312
C	12.4216	10.7729	4.9852
H	13.2296	10.4531	5.6442
H	11.5064	10.3041	5.3456
H	12.3064	11.8507	5.1051
C	13.8358	15.3381	7.1744
H	13.1186	15.6198	7.9454
H	14.6798	14.8694	7.6814
H	13.3681	14.5745	6.553
H	12.3565	18.8177	1.7758
C	11.2472	18.2764	11.4972
C	11.034	19.7693	11.8185
C	11.039	17.4817	12.8049
C	12.7027	18.054	11.0388
H	13.4124	18.3229	11.8219
H	12.8793	17.0088	10.7819
H	12.955	18.6514	10.163
H	11.6843	20.099	12.6295
H	11.2441	20.4109	10.963
H	10.0054	19.962	12.1255
H	11.7305	17.8106	13.5816
H	10.0318	17.6142	13.2017
H	11.2108	16.4143	12.6613
H	7.1729	7.9571	4.3675
C	8.9999	20.8606	2.4843
C	8.1652	11.5299	-0.9343
H	9.1475	11.403	-1.3928
H	7.7487	10.531	-0.8018
C	7.274	12.3575	-1.8682
C	6.5416	17.6096	7.1608
C	5.8477	17.4054	5.811

H	5.8513	17.3788	7.9727
H	6.8213	18.6582	7.2777
C	9.7264	18.8186	6.7008
H	9.9082	19.8775	6.886
H	8.8025	18.7772	6.1279
C	7.4424	15.3543	9.7426
H	7.3023	15.226	10.8165
H	6.4847	15.7069	9.3708
C	5.8564	12.5682	-1.3112
H	7.7423	13.3251	-2.0521
H	7.2261	11.8519	-2.8323
C	4.9165	13.3197	-2.2679
H	5.4135	11.6109	-1.0334
H	5.9376	13.1438	-0.389
C	4.4591	12.4824	-3.4731
H	4.0348	13.6348	-1.7078
H	5.3981	14.2355	-2.6127
C	3.4631	13.2351	-4.363
H	5.3184	12.1898	-4.0766
H	3.9983	11.5572	-3.1243
H	3.1547	12.62	-5.2088
H	2.565	13.509	-3.8084
H	3.9025	14.15	-4.7618
C	7.6626	6.1399	6.4277
H	9.1794	7.5525	5.8136
H	8.2714	8.0554	7.2185
C	8.5776	5.2975	7.3324
H	6.657	6.1729	6.8478
H	7.5738	5.659	5.4524
C	8.1285	3.8333	7.4746
H	9.5879	5.3092	6.9206
H	8.6507	5.7559	8.3198
C	6.8176	3.6605	8.2549
H	8.0342	3.3754	6.4889
H	8.9128	3.2763	7.9891
H	6.5834	2.6037	8.3857
H	6.8854	4.1081	9.2468
H	5.9754	4.1175	7.7357
C	7.4958	20.6781	2.7398
H	9.2053	21.9143	2.2913
H	9.2886	20.3234	1.5802
C	6.6415	21.2524	1.5991
H	7.2742	19.6175	2.8682
H	7.2244	21.1659	3.6769
C	5.1366	21.0868	1.8451
H	6.8703	22.3116	1.4723
H	6.9111	20.7667	0.6606
C	4.285	21.6684	0.7113
H	4.9023	20.0297	1.9581
H	4.8609	21.5668	2.7849
H	3.2225	21.5291	0.9136
H	4.4621	22.7377	0.5918
H	4.507	21.1832	-0.2396
C	4.6454	18.3403	5.6233
H	6.5616	17.5836	5.0061
H	5.5215	16.3694	5.7266
C	3.9506	18.139	4.2701
H	3.9269	18.1802	6.4288
H	4.9759	19.3769	5.7075
C	2.7697	19.0973	4.0641
H	4.6729	18.2681	3.4636
H	3.5926	17.1141	4.1966
C	2.037	18.8503	2.7404
H	2.0617	18.992	4.8871
H	3.1223	20.1291	4.0941
H	1.2072	19.5475	2.6205
H	2.7016	18.9804	1.8861
H	1.6283	17.8403	2.697
C	4.6441	13.5547	9.2465
C	3.6519	14.7255	9.297
H	4.1089	12.622	9.0685
H	5.1334	13.455	10.2177
C	2.9299	14.9577	7.9623
H	2.9172	14.5283	10.0788
H	4.1698	15.6372	9.5941
C	1.8896	16.0841	8.0326
H	3.6699	15.2078	7.2043
H	2.4484	14.0333	7.6398
C	1.1839	16.3122	6.6881
H	1.1466	15.8476	8.796
H	2.3737	17.0088	8.3504
C	0.1542	17.4457	6.75
H	1.9212	16.5428	5.9192
H	0.6877	15.3936	6.3717
H	-0.3236	17.5901	5.7805
H	-0.6296	17.2294	7.4766
H	0.6211	18.3901	7.0318
C	9.2688	16.6097	0.0784
C	7.7827	16.7093	0.438
H	9.6954	17.6062	-0.0357
H	9.3885	16.1068	-0.8821
C	7.0098	17.6535	-0.4965
H	7.3516	15.709	0.4116
H	7.687	17.0672	1.4638
C	5.5106	17.7552	-0.1687
H	7.4504	18.6482	-0.4195
H	7.1424	17.3485	-1.5356
C	4.6958	16.5516	-0.6666
H	5.3732	17.8877	0.905
H	5.1139	18.6584	-0.6348
C	3.2005	16.6779	-0.3536
H	4.8228	16.4513	-1.7452

H	5.0769	15.63	-0.2279
H	2.6515	15.8141	-0.73
H	3.0209	16.7408	0.7193
H	2.7736	17.5682	-0.8164
C	7.786	10.1766	2.3518
H	7.2257	10.0932	1.4215
H	7.923	9.1502	2.6837
C	6.1673	11.143	7.1328
H	5.1637	11.3077	7.522
H	6.3643	10.0905	7.329
C	9.1609	14.5092	5.0748
N	7.9371	14.2861	5.8493
H	7.711	15.1275	6.3705
C	8.9432	15.6976	4.1368
C	9.4736	13.2668	4.2499
C	10.325	14.7741	6.0312
H	7.1738	14.0264	5.2408
H	8.6792	16.6043	4.6789
H	8.1643	15.5065	3.3983
H	8.0796	13.5363	6.5145
H	10.4063	13.3926	3.6982
H	9.5761	12.3709	4.8645
H	8.6953	13.085	3.5112
H	9.8569	15.9092	3.5841
H	11.252	14.9348	5.4793
H	10.1602	15.6545	6.6464
H	10.4863	13.9363	6.7048

______________________--

Energy = -4646.745445 a.u.

0 imaginary frequency

$6b^{+}\subset 2b^{1,2\text{-}Alt}$

C	0.5643	0.6506	0.5572
N	-0.9472	0.6043	0.7111
H	-1.4478	1.2586	-0.0177
C	0.8824	-0.0361	-0.7676
C	1.1863	-0.081	1.742
C	0.9573	2.1233	0.571
H	-1.3362	-0.4192	0.6064
H	0.4002	0.4629	-1.6131
H	0.5847	-1.0883	-0.7551
H	-1.2658	0.9568	1.7032
H	2.2715	-0.0073	1.6596
H	0.8771	0.3596	2.6938
H	0.9337	-1.1382	1.7412
H	1.9585	-0.0119	-0.9374
H	2.0367	2.2047	0.4325
H	0.4862	2.6754	-0.2397
H	0.6953	2.5966	1.5211
O	-4.2821	0.9504	2.4183
O	-0.2246	-2.3092	4.1167
O	-2.3798	2.7606	-0.7279
O	0.3534	2.0886	-3.8847
O	2.4332	-1.7038	-2.9304
O	3.2473	-2.5731	0.8116
C	-0.9818	-2.5077	2.9747
C	5.365	0.5226	-3.0267
H	5.6233	1.4858	-3.4503
C	-2.2325	-1.8792	2.8633
C	-1.2586	0.9587	4.5188
H	-0.725	0.2554	5.1459
C	4.0298	0.1008	-3.1261
C	-2.9815	-2.0753	1.6902
H	-3.953	-1.6006	1.6342
C	3.7091	-1.2063	-2.7034
C	-0.4471	-3.2634	1.9047
C	1.2716	2.7769	-3.1013
C	-2.4741	4.4901	0.9478
C	-1.2245	-3.4079	0.7552
H	-0.813	-3.9799	-0.0668
C	3.2203	-4.0743	-1.0855
C	-2.6369	2.692	2.8049
C	3.4916	3.0122	-2.2054
H	4.4962	2.6303	-2.1003
C	-2.3886	0.481	3.8374
C	-3.1103	1.3782	3.0264
C	-0.8202	2.2845	4.4131
C	2.6852	-3.6555	0.1496
C	-1.506	3.1149	3.5111
H	-1.1627	4.1321	3.3521
C	2.5861	2.2829	-2.9876
C	-2.1608	5.7978	1.3427
H	-2.5573	6.1458	2.2901
C	4.674	-2.0197	-2.0788
C	3.1433	4.205	-1.5617
C	2.9858	1.0141	-3.7562
H	2.0754	0.4456	-3.9422
C	-2.5132	-2.8612	0.6317
C	-1.993	4.0366	-0.292
C	0.8555	3.9262	-2.399
C	1.5992	-4.3365	0.7288
C	5.97	-1.5104	-1.9416
H	6.7145	-2.1491	-1.4741
C	-1.1897	4.8502	-1.1135
C	1.8125	4.6292	-1.6643
H	1.5008	5.5197	-1.1316
C	-3.3474	-3.1644	-0.6272
C	-0.9388	6.1572	-0.678
H	-0.3692	6.8066	-1.3363
C	1.1199	-5.4971	0.0994
H	0.3219	-6.0417	0.5925

C	6.3614	-0.2611	-2.4405
C	1.6404	-5.967	-1.1072
C	-1.4074	6.6636	0.5427
C	2.6712	-5.2113	-1.6857
H	3.1085	-5.5458	-2.6225
C	0.3336	2.8515	5.264
C	1.1678	-7.2711	-1.7771
C	7.8379	0.1734	-2.3628
C	0.9869	1.7756	6.1522
H	0.274	1.3445	6.8622
H	1.797	2.2242	6.7349
H	1.418	0.9607	5.5613
C	0.3212	2.5385	-5.2558
H	-0.0748	3.5639	-5.2917
H	1.3447	2.5653	-5.6506
C	-0.2371	3.9581	6.1848
H	-0.6726	4.7801	5.6088
H	0.5564	4.3749	6.8141
H	-1.0166	3.5595	6.8417
C	8.7086	-0.8391	-3.1455
H	9.7636	-0.5474	-3.1022
H	8.4111	-0.88	-4.1982
H	8.6266	-1.85	-2.7357
C	-0.3417	-3.3416	5.1192
C	0.6132	-3.0177	6.257
H	-1.3803	-3.3876	5.4762
C	-2.6664	-2.5374	-1.8654
H	-2.6125	-1.4465	-1.7768
H	-3.2335	-2.7692	-2.7735
H	-1.6477	-2.9117	-2.0031
C	-3.4447	-4.6965	-0.8245
H	-3.908	-5.1753	0.0439
H	-2.4635	-5.1544	-0.9769
H	-4.0565	-4.9248	-1.7037
C	1.4308	3.4616	4.3618
H	2.2219	3.901	4.9784
H	1.0429	4.2535	3.7143
H	1.8902	2.6984	3.7252
C	4.155	5.0477	-0.7597
C	-4.7799	-2.6137	-0.5185
H	-5.3143	-3.0496	0.3317
H	-5.3414	-2.8611	-1.4243
H	-4.7965	-1.5253	-0.4113
C	8.071	1.5703	-2.9685
H	7.5053	2.3449	-2.4405
H	7.7988	1.6071	-4.0283
H	9.1312	1.8308	-2.8941
C	8.2982	0.2059	-0.8869
H	7.7063	0.9212	-0.3061
H	9.3498	0.506	-0.823
H	8.2055	-0.7738	-0.4089
C	2.3534	-8.264	-1.8455
H	2.7271	-8.4978	-0.8435
H	3.1877	-7.8624	-2.428
H	2.0385	-9.201	-2.3174
C	0.0226	-7.947	-0.9998
H	0.3252	-8.2249	0.0149
H	-0.2796	-8.8645	-1.5138
H	-0.8614	-7.3039	-0.9299
C	5.5651	4.4285	-0.7588
H	6.2459	5.0632	-0.1832
H	5.9724	4.3437	-1.7715
H	5.5749	3.4339	-0.3002
C	4.2512	6.4577	-1.3903
H	3.2878	6.9762	-1.3771
H	4.5846	6.3998	-2.4314
H	4.9683	7.0746	-0.838
C	0.6684	-6.9751	-3.2098
H	0.3471	-7.901	-3.6993
H	1.4496	-6.5273	-3.8312
H	-0.1835	-6.2878	-3.1951
C	3.6887	5.1789	0.7096
H	2.6992	5.6393	0.7859
H	4.3893	5.8022	1.2759
H	3.6425	4.199	1.1968
H	3.3611	1.3275	-4.7413
C	-1.1101	8.1223	0.9389
C	-1.73	9.0731	-0.1131
C	-1.694	8.4842	2.3177
C	0.4199	8.3451	0.9913
H	0.643	9.3795	1.2733
H	0.8892	7.6857	1.7291
H	0.8942	8.1596	0.0229
H	-1.5255	10.1159	0.1515
H	-1.3213	8.8989	-1.1128
H	-2.8157	8.9441	-0.1678
H	-1.452	9.5238	2.5571
H	-2.7845	8.3896	2.3385
H	-1.2785	7.8591	3.1152
H	-0.1037	-4.3178	4.6773
C	-0.5395	1.5998	-6.0861
C	4.29	-2.9392	1.7399
H	3.9369	-3.7635	2.3736
H	5.1669	-3.3021	1.1856
C	4.6585	-1.7345	2.5943
C	-3.5884	2.8182	-1.5356
C	-4.2577	1.4581	-1.6282
H	-4.2701	3.5431	-1.0758
H	-3.3285	3.1939	-2.5327
C	-0.6131	4.3672	-2.4403
H	-0.7221	5.1794	-3.1708
H	-1.1928	3.5263	-2.8203

C	-3.3613	3.627	1.8351
H	-4.0053	4.3004	2.4146
H	-4.0265	3.0182	1.2211
C	5.2684	-0.5688	1.8046
H	3.762	-1.3977	3.1319
H	5.3628	-2.0855	3.3585
C	5.5252	0.6955	2.6397
H	6.2112	-0.8995	1.3488
H	4.6054	-0.3236	0.9675
C	6.5802	0.5412	3.7439
H	5.8411	1.5026	1.9643
H	4.5796	1.0337	3.0901
C	6.8537	1.8475	4.4954
H	6.2624	-0.2261	4.4613
H	7.514	0.1726	3.298
H	7.6097	1.7103	5.2747
H	7.2148	2.6277	3.8158
H	5.9449	2.2254	4.9779
C	0.5635	-4.0603	7.3819
H	1.6306	-2.9471	5.8526
H	0.3659	-2.0257	6.6563
C	1.5251	-3.7378	8.5344
H	-0.4635	-4.1328	7.7621
H	0.8096	-5.0511	6.9744
C	1.5492	-4.7866	9.658
H	2.5393	-3.6312	8.1265
H	1.2637	-2.7582	8.9594
C	0.2391	-4.9081	10.4445
H	1.8145	-5.7643	9.2333
H	2.3584	-4.5305	10.3529
H	0.3397	-5.6265	11.2639
H	-0.0492	-3.9454	10.8823
H	-0.5897	-5.2478	9.8148
C	-2.0236	1.5751	-5.7026
H	-0.4387	1.914	-7.1337
H	-0.1201	0.5875	-6.0248
C	-2.8645	0.7075	-6.6473
H	-2.1248	1.2105	-4.6722
H	-2.4154	2.6027	-5.7092
C	-4.3537	0.6604	-6.2874
H	-2.7539	1.0816	-7.6749
H	-2.4629	-0.3159	-6.6555
C	-5.1803	-0.2024	-7.2458
H	-4.4648	0.2783	-5.263
H	-4.7553	1.6831	-6.2772
H	-6.2375	-0.2163	-6.9626
H	-5.1172	0.175	-8.2723
H	-4.8243	-1.2388	-7.2537
C	-5.5736	1.5645	-2.4177
H	-3.583	0.7456	-2.1201
H	-4.4499	1.0828	-0.6148

C	-6.3432	0.2447	-2.54
H	-6.2204	2.3119	-1.9374
H	-5.3595	1.9499	-3.4235
C	-7.6437	0.3761	-3.3429
H	-5.7007	-0.5123	-3.0112
H	-6.5754	-0.1367	-1.5359
C	-8.4191	-0.9397	-3.4539
H	-8.2811	1.139	-2.8761
H	-7.4103	0.7504	-4.3486
H	-9.3394	-0.8123	-4.0318
H	-7.821	-1.7131	-3.9489
H	-8.699	-1.3204	-2.4649
C	-5.4732	1.3889	3.1211
C	-6.7173	0.889	2.4033
H	-5.436	1.0109	4.1522
H	-5.4759	2.4845	3.1693
C	-6.9695	-0.6194	2.5114
H	-7.5714	1.4282	2.8342
H	-6.6636	1.1931	1.3498
C	-8.2718	-1.0598	1.8312
H	-6.1274	-1.168	2.0728
H	-7.0019	-0.9015	3.5734
C	-8.5552	-2.5614	1.961
H	-9.1128	-0.4952	2.2579
H	-8.2358	-0.7882	0.7661
C	-9.8553	-2.9909	1.2753
H	-7.7138	-3.1272	1.5376
H	-8.5957	-2.8301	3.0251
H	-10.0295	-4.0654	1.3871
H	-10.7192	-2.4685	1.7008
H	-9.8309	-2.7682	0.2024
C	2.2935	-2.3507	-4.2086
C	0.8236	-2.6458	-4.4674
H	2.6962	-1.6962	-4.9943
H	2.8825	-3.2785	-4.2207
C	0.6097	-3.2651	-5.8579
H	0.4536	-3.311	-3.6792
H	0.259	-1.7076	-4.3859
C	-0.8613	-3.4525	-6.2627
H	1.0949	-2.6232	-6.6055
H	1.126	-4.2337	-5.9122
C	-1.6286	-4.5135	-5.4635
H	-1.3833	-2.4881	-6.1789
H	-0.898	-3.7226	-7.3262
C	-3.076	-4.6829	-5.9357
H	-1.1027	-5.474	-5.5463
H	-1.6239	-4.2541	-4.3972
H	-3.6006	-5.4492	-5.3561
H	-3.6385	-3.7468	-5.8366
H	-3.1169	-4.9791	-6.9896
C	-2.7906	-0.9852	3.9703

H	-3.88	-1.0669	3.9587
H	-2.4459	-1.346	4.9413
C	4.4813	-3.4901	-1.7107
H	4.6636	-4.0688	-2.6253
H	5.3209	-3.751	-1.055
C	0.9502	-3.8853	2.0276
H	0.8753	-4.7574	2.691
H	1.5922	-3.1719	2.5473

________________________--

Energy = -4646.731859 a.u.

0 imaginary frequency

6c$^+$⊂2b$^{1,2,3\text{-}Alt}$

C	0.8159	0.4955	1.058
N	1.7205	0.272	-0.0803
H	1.7723	-0.7898	-0.3626
C	-0.6086	0.3624	0.5252
C	1.1379	-0.5753	2.0991
H	1.4021	0.8612	-0.9529
H	-0.7955	-0.639	0.1273
H	-0.8177	1.0991	-0.2557
H	2.7449	0.6029	0.146
H	-1.3188	0.5384	1.3327
H	0.4895	-0.4372	2.9662
H	0.9613	-1.5813	1.7143
H	2.1763	-0.5045	2.4407
O	4.426	0.9499	-0.259
O	2.2998	3.9701	-0.9906
O	2.1901	-2.5163	-0.7883
O	-1.8075	-3.7628	-0.1351
O	-3.8131	0.0955	0.2234
O	-1.7913	3.5212	1.2394
C	1.6768	3.2878	-2.0256
C	-4.0024	-0.8862	3.7655
H	-3.9693	-1.801	4.3442
C	2.4411	2.4605	-2.871
C	5.2377	-1.7826	-2.5913
H	5.5051	-2.8318	-2.5346
C	-3.736	-0.969	2.3905
C	1.7872	1.7829	-3.9113
H	2.3867	1.1605	-4.5639
C	-3.9221	0.1886	1.6036
C	0.2743	3.3996	-2.1856
C	-1.2716	-3.6363	1.1405
C	4.3308	-3.1575	0.1972
C	-0.3171	2.6966	-3.2362
H	-1.3876	2.78	-3.3614
C	-3.9178	3.1607	0.1589
C	4.4869	0.92	-2.6735
C	-1.4467	-2.8411	3.4066
H	-1.9945	-2.29	4.1563
C	4.9435	-1.1215	-1.3905
C	4.6337	0.2438	-1.4502
C	5.2109	-1.1285	-3.8284
C	-2.5698	3.5475	0.0868
C	4.8079	0.2187	-3.8388
H	4.7531	0.7504	-4.7843
C	-1.9887	-2.9131	2.1149
C	5.0743	-4.1448	0.8666
H	6.1257	-3.9435	1.0371
C	-4.2365	1.4252	2.2
C	-0.2405	-3.4544	3.7596
C	-3.3513	-2.3004	1.7563
H	-3.3837	-2.1981	0.6736
C	0.4147	1.9049	-4.1354
C	2.9689	-3.4112	-0.0396
C	0.0056	-4.1695	1.4272
C	-2.0176	4.0242	-1.1206
C	-4.4078	1.4556	3.5892
H	-4.6598	2.41	4.0435
C	2.3527	-4.5779	0.459
C	0.4779	-4.0896	2.7384
H	1.4462	-4.5267	2.9578
C	-0.2846	1.2813	-5.3574
C	3.1484	-5.5231	1.1105
H	2.6656	-6.4292	1.4658
C	-2.8862	4.2625	-2.1926
H	-2.4746	4.7254	-3.085
C	-4.3411	0.3134	4.3968
C	-4.2539	3.9633	-2.1433
C	4.5234	-5.3461	1.3159
C	-4.7261	3.3689	-0.9684
H	-5.7749	3.1066	-0.8858
C	5.6076	-1.821	-5.1465
C	-5.1765	4.3558	-3.3137
C	-4.6604	0.4112	5.9012
C	6.0004	-3.2956	-4.9371
H	6.861	-3.3985	-4.2685
H	6.2768	-3.7412	-5.8972
H	5.1743	-3.8864	-4.5275
C	-2.2362	-5.0928	-0.5134
H	-2.2878	-5.0659	-1.607
H	-1.4653	-5.8175	-0.2323
C	6.8205	-1.0805	-5.7601
H	6.5926	-0.0328	-5.9774
H	7.1193	-1.557	-6.6996
H	7.6788	-1.1028	-5.0811
C	-6.092	0.9684	6.0883
H	-6.335	1.0425	7.1539
H	-6.8319	0.3137	5.6169

H	-6.2033	1.9658	5.6533
C	2.7181	5.3142	-1.3126
C	3.3004	5.953	-0.0619
H	3.4625	5.2791	-2.1201
C	-1.6267	0.6351	-4.9487
H	-1.4875	-0.116	-4.1654
H	-2.0854	0.1453	-5.8139
H	-2.3439	1.372	-4.5787
C	-0.563	2.408	-6.3833
H	0.3682	2.8819	-6.7098
H	-1.2031	3.1859	-5.9562
H	-1.0675	2.0042	-7.268
C	4.4245	-1.7741	-6.142
H	4.1241	-0.7487	-6.3763
H	4.7032	-2.2585	-7.0837
H	3.55	-2.2977	-5.7409
C	0.3063	-3.4642	5.2008
C	0.5836	0.2078	-6.0416
H	1.5044	0.6276	-6.4584
H	0.0303	-0.2394	-6.873
H	0.8578	-0.5965	-5.3509
C	-4.5873	-0.956	6.6069
H	-3.5852	-1.3936	6.551
H	-5.2998	-1.6721	6.1847
H	-4.8327	-0.8359	7.6666
C	-3.6502	1.3616	6.5853
H	-2.6268	0.9851	6.482
H	-3.8724	1.4493	7.6544
H	-3.6826	2.3671	6.1557
C	-5.2211	5.9006	-3.4091
H	-4.2277	6.3228	-3.5888
H	-5.6087	6.3395	-2.4843
H	-5.872	6.2139	-4.2327
C	-4.6383	3.7837	-4.6448
H	-3.6349	4.1535	-4.8766
H	-5.2917	4.0761	-5.4738
H	-4.6004	2.6903	-4.6171
C	-0.5999	-2.6924	6.1781
H	-0.1727	-2.7291	7.1849
H	-1.6032	-3.1271	6.2317
H	-0.6975	-1.6375	5.8999
C	0.4015	-4.9301	5.6892
H	1.0736	-5.5231	5.0619
H	-0.5812	-5.4122	5.6751
H	0.7832	-4.967	6.7154
C	-6.6165	3.8432	-3.1215
H	-7.2263	4.127	-3.9847
H	-7.0899	4.2739	-2.2336
H	-6.6529	2.7521	-3.0351
C	1.7144	-2.8253	5.2459
H	2.4219	-3.3433	4.592

H	2.1155	-2.8645	6.2645
H	1.6799	-1.7745	4.9392
H	-4.1238	-3.0367	2.0205
C	5.3487	-6.4397	2.0182
C	5.2603	-7.7497	1.1994
C	6.8342	-6.0558	2.1532
C	4.7819	-6.6832	3.4372
H	5.3586	-7.462	3.9472
H	4.8344	-5.7725	4.043
H	3.7381	-7.0102	3.4108
H	5.8368	-8.542	1.6885
H	4.2293	-8.1022	1.1022
H	5.6635	-7.611	0.191
H	7.3786	-6.865	2.649
H	7.3048	-5.8937	1.178
H	6.9708	-5.1522	2.7564
H	1.859	5.8904	-1.6799
C	-3.578	-5.525	0.0754
C	-1.7771	4.8073	1.9014
H	-1.4261	5.5711	1.1947
H	-2.8025	5.0785	2.1896
C	-0.8723	4.7672	3.1236
C	1.9874	-2.9569	-2.1672
C	0.5816	-2.6522	-2.6734
H	2.75	-2.4791	-2.7898
H	2.1645	-4.0362	-2.1957
C	0.8572	-4.831	0.3389
H	0.7066	-5.9174	0.3724
H	0.4847	-4.5045	-0.6309
C	-1.3578	3.8359	4.2416
H	0.1402	4.4836	2.8084
H	-0.7999	5.7968	3.4952
C	-0.4025	3.7386	5.4417
H	-2.3409	4.1832	4.5905
H	-1.5254	2.8361	3.8257
C	-0.2358	5.0373	6.2431
H	-0.7693	2.9549	6.1174
H	0.5849	3.3991	5.0943
C	0.6635	4.87	7.4716
H	0.1767	5.8257	5.6008
H	-1.2262	5.3913	6.5606
H	0.76	5.8075	8.0278
H	0.2615	4.1162	8.1577
H	1.6723	4.5504	7.1849
C	3.7692	7.3946	-0.3012
H	2.5419	5.9307	0.7308
H	4.1405	5.3411	0.2911
C	4.3598	8.0446	0.9579
H	4.5116	7.4038	-1.1094
H	2.9234	8.0004	-0.6559
C	4.7855	9.511	0.7799

H	3.617	7.9864	1.765
H	5.2241	7.4556	1.297
C	5.9539	9.7224	-0.1896
H	3.9203	10.0996	0.446
H	5.0618	9.9126	1.7625
H	6.2452	10.7765	-0.2271
H	6.8338	9.1471	0.1209
H	5.7021	9.4187	-1.211
C	-4.8106	-4.8466	-0.5335
H	-3.6572	-6.6104	-0.0777
H	-3.5547	-5.3785	1.163
C	-6.1252	-5.3351	0.088
H	-4.7303	-3.7592	-0.4184
H	-4.8266	-5.0393	-1.6158
C	-7.3762	-4.6872	-0.5174
H	-6.1945	-6.4265	-0.0238
H	-6.1082	-5.1453	1.171
C	-8.6808	-5.2006	0.0992
H	-7.3164	-3.5973	-0.3895
H	-7.3878	-4.866	-1.6012
H	-9.5533	-4.7181	-0.3522
H	-8.7877	-6.2815	-0.0444
H	-8.7139	-5.0063	1.1771
C	0.308	-3.4524	-3.9603
H	-0.155	-2.921	-1.9082
H	0.4668	-1.5768	-2.8632
C	-1.1288	-3.349	-4.4836
H	1.0059	-3.1386	-4.7495
H	0.5328	-4.5111	-3.7671
C	-1.3806	-4.2341	-5.7114
H	-1.8269	-3.6294	-3.682
H	-1.3594	-2.3065	-4.7345
C	-2.8131	-4.1403	-6.2435
H	-0.676	-3.9571	-6.5071
H	-1.1526	-5.2779	-5.4564
H	-2.9605	-4.7912	-7.1107
H	-3.5415	-4.4352	-5.4796
H	-3.0543	-3.1171	-6.5526
C	5.6157	1.6113	0.2492
C	5.2964	2.3164	1.5577
H	6.4031	0.859	0.3837
H	5.9586	2.3265	-0.5073
C	5.0855	1.3813	2.7554
H	6.1354	2.9912	1.7722
H	4.4183	2.9562	1.4025
C	4.798	2.1273	4.064
H	4.2604	0.6876	2.5452
H	5.9803	0.7563	2.8828
C	4.6239	1.1976	5.2714
H	5.6164	2.8326	4.265
H	3.894	2.7418	3.944
C	4.3335	1.9482	6.5741
H	3.8105	0.4872	5.0683
H	5.5315	0.5912	5.3925
H	4.2218	1.2577	7.4156
H	5.1437	2.6432	6.8204
H	3.4088	2.5317	6.4988
C	-5.0577	-0.2487	-0.4132
C	-4.8221	-0.5395	-1.8877
H	-5.4894	-1.1282	0.0852
H	-5.7731	0.577	-0.2933
C	-6.1257	-0.9802	-2.5737
H	-4.4063	0.3558	-2.3628
H	-4.0661	-1.3303	-1.9764
C	-5.9879	-1.3818	-4.0506
H	-6.5392	-1.8338	-2.0206
H	-6.8724	-0.1776	-2.4896
C	-5.6608	-0.2271	-5.0058
H	-5.221	-2.1636	-4.1465
H	-6.9308	-1.8437	-4.3718
C	-5.6443	-0.6519	-6.4774
H	-6.4015	0.5725	-4.867
H	-4.6881	0.2076	-4.7433
H	-5.4068	0.19	-7.1354
H	-4.8998	-1.436	-6.6564
H	-6.6177	-1.0494	-6.7853
C	-4.5876	2.7137	1.4508
H	-5.6642	2.6715	1.2417
H	-4.4797	3.5243	2.1807
C	-0.5337	4.3279	-1.2645
H	-0.4274	5.3505	-1.6532
H	-0.0592	4.3301	-0.283
C	5.0354	-1.8241	-0.0477
H	6.0967	-1.9867	0.1748
H	4.688	-1.1256	0.7195
C	3.963	2.349	-2.7253
H	4.4278	2.8572	-3.5788
H	4.2576	2.8927	-1.8286
H	0.9723	1.4897	1.4765

________________________--

Energy = -4607.415004 a.u.

0 imaginary frequency

$6c^{+}\subset 2b^{cone}$

O	-1.0409	3.0581	2.4387
O	-3.9818	2.4604	0.3128
O	2.6073	0.7305	1.3849
O	4.3527	-1.925	-0.9847
O	1.0429	-1.8979	-2.8419
O	-2.5005	0.1606	-2.5387
C	-4.4471	1.2726	0.8615

C	0.5798	-5.49	-2.1745
H	1.1762	-6.317	-1.8058
C	-5.0181	0.3023	0.0189
C	-2.4268	0.7416	4.9338
H	-3.398	0.4157	5.2914
C	1.1949	-4.2352	-2.2747
C	-5.5183	-0.872	0.5926
H	-5.959	-1.6088	-0.0671
C	0.4485	-3.1657	-2.795
C	-4.3221	1.0478	2.2474
C	3.7084	-2.6621	-0.0015
C	2.6115	1.3399	3.741
C	-4.8567	-0.1354	2.7674
H	-4.8007	-0.2943	3.8385
C	-4.7715	-0.6319	-2.3605
C	0.0571	1.6621	4.062
C	2.2099	-4.4402	0.6466
H	1.5572	-5.2521	0.3506
C	-5.1436	0.5514	-1.4842
H	-6.1813	0.8304	-1.7078
H	-4.5226	1.4117	-1.7316
C	-2.3868	1.6377	3.8601
C	-1.1288	2.1285	3.4646
C	-1.2736	0.27	5.5755
C	-3.4489	-0.8241	-2.8013
C	-0.0397	0.7259	5.0971
H	0.8767	0.3652	5.5449
C	2.849	-3.7113	-0.3699
C	3.2335	1.2282	4.9959
H	2.8349	1.8325	5.803
C	-0.8841	-3.3349	-3.2212
C	2.4051	-4.1642	2.006
C	2.6429	-4.0692	-1.8399
H	3.1335	-3.3052	-2.4418
C	-5.4797	-1.1064	1.9704
C	3.1509	0.6085	2.6716
C	3.8795	-2.3125	1.3528
C	-3.0837	-1.9652	-3.531
C	-1.4541	-4.6008	-3.0594
H	-2.4859	-4.7362	-3.3565
C	-3.67	2.0854	3.164
H	-3.4568	2.9733	2.5718
C	4.2251	-0.2805	2.8685
C	-1.6332	-2.1984	-3.9268
H	-1.0782	-1.2767	-3.7593
H	-1.595	-2.4053	-5.0052
C	3.2287	-3.0737	2.3251
H	3.3797	-2.8006	3.3645
C	-6.1116	-2.3525	2.6218
C	4.7809	-0.3747	4.1461
H	5.6169	-1.0546	4.2838
C	-4.1006	-2.8518	-3.9218
H	-3.8313	-3.683	-4.5622
C	-0.7525	-5.6985	-2.5413
C	-5.4378	-2.6724	-3.5566
C	4.3208	0.3844	5.2329
C	-5.7368	-1.5624	-2.7506
H	-6.7627	-1.3912	-2.4362
C	-1.3969	-0.6883	6.7763
C	-6.5646	-3.6217	-4.0066
C	-1.4525	-7.0647	-2.4145
C	-2.164	-1.9639	6.3573
H	-3.1765	-1.7353	6.0122
H	-2.2533	-2.6512	7.2055
H	-1.6438	-2.4885	5.5489
C	5.6713	-2.4098	-1.3232
H	6.3048	-2.3999	-0.4261
H	5.5977	-3.451	-1.6658
C	-2.171	0.0202	7.9139
H	-1.6465	0.9236	8.2412
H	-2.273	-0.6451	8.7782
H	-3.177	0.314	7.6004
C	-2.6711	-6.9279	-1.4708
H	-3.1876	-7.889	-1.3765
H	-3.3952	-6.1961	-1.8412
H	-2.359	-6.6119	-0.4699
C	-4.9889	3.4839	0.1938
C	-4.3921	4.6759	-0.5376
H	-5.8559	3.0882	-0.3527
C	-6.7907	-3.272	1.589
H	-7.599	-2.7596	1.0573
H	-7.2284	-4.1364	2.0978
H	-6.0798	-3.6516	0.8479
C	-5.0259	-3.1745	3.3537
H	-4.2768	-3.5464	2.6471
H	-5.4745	-4.0392	3.855
H	-4.507	-2.5824	4.1129
C	-0.0238	-1.1172	7.3275
H	-0.1631	-1.7974	8.1734
H	0.5564	-0.2613	7.6873
H	0.5722	-1.644	6.5746
C	1.7974	-5.0225	3.1321
C	-7.1857	-1.9045	3.6421
H	-6.7606	-1.2903	4.4413
H	-7.6544	-2.7777	4.1089
H	-7.9707	-1.319	3.153
C	-1.9331	-7.5311	-3.8093
H	-1.0906	-7.6383	-4.5
H	-2.6427	-6.8273	-4.2544
H	-2.4339	-8.502	-3.7325
C	-0.5205	-8.1492	-1.842
H	0.3513	-8.3197	-2.4822

H	-1.0619	-9.0971	-1.7681
H	-0.1659	-7.8959	-0.8373
C	-7.6187	-2.8234	-4.8106
H	-7.1727	-2.3724	-5.7028
H	-8.0633	-2.0198	-4.2164
H	-8.43	-3.4842	-5.1341
C	-6.0479	-4.7648	-4.9004
H	-5.5817	-4.3892	-5.8171
H	-6.8836	-5.4056	-5.197
H	-5.3216	-5.3972	-4.3784
C	0.9836	-4.135	4.1029
H	0.5728	-4.7447	4.9144
H	0.1471	-3.6502	3.5896
H	1.5978	-3.3526	4.5576
C	0.8688	-6.1274	2.5936
H	1.4025	-6.829	1.9444
H	0.0217	-5.7169	2.034
H	0.4613	-6.7035	3.4298
C	-7.2375	-4.2493	-2.7629
H	-8.0452	-4.9238	-3.067
H	-7.6713	-3.4898	-2.1058
H	-6.5165	-4.8281	-2.1759
C	2.9512	-5.6991	3.9124
H	3.5466	-6.3409	3.2552
H	2.55	-6.3201	4.7205
H	3.6242	-4.962	4.3601
H	3.1629	-5.0138	-2.0416
C	5.0079	0.2625	6.6053
C	4.9061	-1.1991	7.103
C	6.4985	0.6549	6.4691
C	4.3652	1.1773	7.6647
H	4.889	1.0592	8.618
H	4.4279	2.234	7.3849
H	3.3127	0.9288	7.8368
H	5.3949	-1.3029	8.0775
H	3.8603	-1.5039	7.2154
H	5.3887	-1.9001	6.4154
H	7.0029	0.5692	7.4374
H	7.0285	0.0104	5.7617
H	6.6025	1.6885	6.1235
H	-4.3976	2.3672	3.9351
H	-5.337	3.7731	1.1958
C	6.2566	-1.5211	-2.4087
C	-2.467	1.1655	-3.5767
H	-2.1526	0.7008	-4.5222
H	-3.4806	1.559	-3.7309
C	-1.5176	2.2842	-3.1805
C	2.9681	1.9394	0.6598
C	4.3371	1.8845	-0.0079
H	2.1782	2.0658	-0.0866
H	2.9193	2.7915	1.345
C	4.7722	-1.1424	1.7458
H	5.7464	-1.5334	2.0647
H	4.9571	-0.5436	0.8551
C	1.3976	2.2416	3.6017
H	1.5937	3.1527	4.1837
H	1.2685	2.5714	2.5721
C	-0.6035	-0.6491	0.0628
N	0.8351	-1.1237	-0.0619
H	1.5206	-0.4895	0.5194
C	-1.2477	-1.276	1.3004
C	-0.5998	0.8711	0.0682
H	0.9617	-2.158	0.2906
C	-1.4767	3.4095	-4.223
H	-1.8287	2.6828	-2.2079
H	-0.5102	1.868	-3.0471
C	-0.4941	4.5305	-3.8615
H	-1.2079	2.995	-5.2048
H	-2.4833	3.8348	-4.3395
C	-0.4645	5.6728	-4.8841
H	-0.7526	4.935	-2.8726
H	0.5167	4.1081	-3.7608
C	0.5265	6.7823	-4.5209
H	-0.2143	5.2667	-5.8734
H	-1.4725	6.0984	-4.9782
H	0.5211	7.5822	-5.2676
H	0.2824	7.2315	-3.5516
H	1.55	6.3953	-4.457
C	-5.394	5.8242	-0.7146
H	-3.5147	5.0282	0.0185
H	-4.0279	4.3424	-1.518
C	-4.801	7.0328	-1.4495
H	-6.2736	5.4621	-1.2648
H	-5.7624	6.1443	0.2699
C	-5.7935	8.1878	-1.6305
H	-3.9202	7.3952	-0.8998
H	-4.4325	6.7136	-2.4349
C	-5.1922	9.39	-2.364
H	-6.6729	7.8255	-2.1797
H	-6.1614	8.5055	-0.6457
H	-5.9239	10.1961	-2.4756
H	-4.3312	9.7964	-1.8211
H	-4.8491	9.1123	-3.3671
C	7.6631	-1.9631	-2.8349
H	5.5836	-1.5301	-3.2758
H	6.2855	-0.4869	-2.0428
C	8.2678	-1.0741	-3.9288
H	8.3276	-1.9625	-1.9598
H	7.6289	-3.0023	-3.1904
C	9.6746	-1.5072	-4.3592
H	7.6041	-1.0747	-4.8053
H	8.301	-0.0341	-3.574

C	10.272	-0.6151	-5.451
H	10.3363	-1.507	-3.4826
H	9.6402	-2.5465	-4.7127
H	11.2743	-0.95	-5.7347
H	9.6516	-0.6231	-6.3543
H	10.3526	0.4246	-5.1144
C	4.6368	3.172	-0.79
H	5.1073	1.7406	0.7595
H	4.3792	1.0174	-0.678
C	6.0306	3.1753	-1.4308
H	3.877	3.3143	-1.5714
H	4.5485	4.0369	-0.1178
C	6.3476	4.4627	-2.2012
H	6.7883	3.0249	-0.649
H	6.1204	2.3148	-2.1099
C	7.7448	4.4602	-2.8282
H	5.5934	4.6101	-2.9859
H	6.2523	5.3213	-1.5232
H	7.9404	5.3904	-3.3702
H	8.5225	4.3515	-2.0641
H	7.8614	3.6322	-3.5371
C	-1.0457	4.4345	2.8609
C	-0.6917	5.3022	1.6629
H	-2.0393	4.6942	3.2533
H	-0.3242	4.579	3.6764
C	-0.7503	6.8023	1.9766
H	0.313	5.031	1.3136
H	-1.3803	5.0592	0.8445
C	-0.4031	7.6847	0.7709
H	-1.7555	7.0619	2.3367
H	-0.0637	7.0326	2.8033
C	-0.4601	9.1868	1.0744
H	0.6019	7.4259	0.4079
H	-1.0915	7.4555	-0.0556
C	-0.1231	10.0603	-0.1374
H	-1.4623	9.4431	1.4434
H	0.2323	9.4159	1.8956
H	-0.1709	11.1247	0.112
H	0.8866	9.8517	-0.5091
H	-0.8222	9.8813	-0.9624
C	1.6965	-1.5628	-4.0892
C	2.3588	-0.2027	-3.944
H	2.4349	-2.3386	-4.3275
H	0.9497	-1.554	-4.8923
C	3.0478	0.2497	-5.2387
H	1.595	0.532	-3.6557
H	3.0868	-0.2532	-3.1246
C	3.6837	1.6413	-5.1336
H	3.8193	-0.4814	-5.5175
H	2.3178	0.2487	-6.06
C	4.3615	2.0998	-6.4308

H	2.912	2.3718	-4.8506
H	4.4202	1.6445	-4.3173
C	4.9834	3.4952	-6.3276
H	5.1358	1.3728	-6.7102
H	3.6255	2.0866	-7.2458
H	5.4539	3.7924	-7.2697
H	4.2272	4.2498	-6.0827
H	5.7525	3.5312	-5.5477
H	-0.6384	-1.0508	2.1857
H	1.145	-1.1374	-1.1172
H	-2.2021	-0.765	1.4556
H	-1.6236	1.2445	0.0395
H	-0.1223	1.2651	0.9661
H	-0.0851	1.2641	-0.8134
H	-1.1004	-1.01	-0.8402
H	-1.3869	-2.3499	1.1758

______________________________--

Energy = -4607.439569 a.u.

0 imaginary frequency

$6c^{+}\subset 2b^{paco}$

O	3.532	2.2654	-2.5069
O	1.4363	-2.7339	-3.848
O	0.7871	3.657	0.191
O	-1.9725	2.5854	3.507
O	-1.974	-1.5974	3.1322
O	0.1297	-4.8042	1.0943
C	2.1228	-2.487	-2.6673
C	-5.6286	-1.2747	2.8456
H	-6.3909	-0.5428	3.0883
C	2.9211	-1.3342	-2.5595
C	0.8698	0.6237	-4.4613
H	0.703	-0.3408	-4.9252
C	-4.2885	-0.9094	3.0464
C	3.6347	-1.1187	-1.367
H	4.2772	-0.2462	-1.3235
C	-3.2979	-1.8768	2.8065
C	1.9993	-3.3844	-1.5844
C	-2.9147	2.588	2.4872
C	0.3506	4.9757	-1.7733
C	2.7382	-3.1276	-0.4273
H	2.6434	-3.8348	0.3895
C	-0.3507	-4.3972	-1.2378
C	1.3484	3.0677	-3.1824
C	-4.8517	1.6272	1.4266
H	-5.6061	0.8524	1.4038
C	2.0765	0.7998	-3.7636
C	2.3268	2.0484	-3.1633
C	-0.0784	1.645	-4.5897
C	-0.7824	-4.4718	0.1003

C	0.1718	2.8411	-3.8992
H	-0.5664	3.6359	-3.924
C	-3.8969	1.5812	2.4515
C	-0.4209	5.9481	-2.4221
H	-0.0988	6.273	-3.4052
C	-3.6223	-3.1407	2.2755
C	-4.8563	2.6255	0.4487
C	-3.9419	0.4929	3.5314
H	-2.9749	0.4864	4.0336
C	3.5876	-2.0179	-0.2942
C	-0.0394	4.5653	-0.4884
C	-2.8347	3.5677	1.4744
C	-2.1137	-4.1712	0.4478
C	-4.9742	-3.4443	2.0877
H	-5.2238	-4.4353	1.7182
C	-1.2021	5.0656	0.1265
C	-2.5489	-4.1594	1.9183
H	-1.6559	-3.9915	2.5215
H	-2.9246	-5.1562	2.1855
C	-3.8194	3.5701	0.485
H	-3.7673	4.3252	-0.2906
C	4.453	-1.8646	0.9712
C	-1.9177	6.0531	-0.563
H	-2.7875	6.4779	-0.0703
C	-3.0195	-3.8889	-0.5823
H	-4.0415	-3.6635	-0.308
C	-6.0056	-2.5401	2.3843
C	-2.6478	-3.8825	-1.9304
C	-1.5537	6.5215	-1.8332
C	-1.2964	-4.1091	-2.2252
H	-0.9512	-4.0658	-3.2532
C	-1.3345	1.5182	-5.4721
C	-3.6556	-3.6503	-3.0714
C	-7.4764	-2.9651	2.2121
C	-1.3901	0.1704	-6.214
H	-0.5283	0.0371	-6.876
H	-2.2906	0.1293	-6.8342
H	-1.428	-0.677	-5.5237
C	-2.3567	3.3467	4.6699
H	-2.4067	4.4138	4.4088
H	-3.359	3.0364	4.9921
C	-1.3178	2.646	-6.533
H	-1.335	3.6398	-6.0762
H	-2.1955	2.5647	-7.1828
H	-0.424	2.5812	-7.1616
C	-7.7672	-4.1791	3.1268
H	-8.8088	-4.4988	3.0149
H	-7.6015	-3.926	4.1789
H	-7.1298	-5.0345	2.8844
C	2.1563	-3.5538	-4.7952
C	1.2799	-3.7702	-6.0186
H	3.0976	-3.058	-5.0705
C	3.5489	-1.7259	2.217
H	2.9483	-0.8102	2.1687
H	4.1582	-1.6749	3.1255
H	2.8618	-2.5705	2.3205
C	5.3456	-3.1198	1.1288
H	5.9956	-3.2495	0.2578
H	4.7522	-4.0311	1.2423
H	5.9812	-3.0231	2.0155
C	-2.6062	1.6578	-4.6052
H	-3.5019	1.6063	-5.2333
H	-2.6324	2.61	-4.0676
H	-2.6694	0.8519	-3.8669
C	-5.9448	2.7208	-0.6379
C	5.3747	-0.6338	0.9046
H	6.0691	-0.6922	0.0609
H	5.9712	-0.5695	1.8198
H	4.8094	0.3003	0.8187
C	-8.4595	-1.8391	2.5838
H	-8.3283	-0.9553	1.9504
H	-8.3535	-1.532	3.6293
H	-9.4874	-2.1892	2.4481
C	-7.7347	-3.3621	0.7395
H	-7.5408	-2.5208	0.0657
H	-8.7775	-3.6692	0.6057
H	-7.1021	-4.1972	0.4242
C	-3.6796	-4.8966	-3.9885
H	-3.9835	-5.7876	-3.43
H	-2.6974	-5.0968	-4.4269
H	-4.3894	-4.7528	-4.8105
C	-5.0857	-3.4078	-2.5542
H	-5.4642	-4.2634	-1.9862
H	-5.7617	-3.2506	-3.4005
H	-5.145	-2.5208	-1.9147
C	-6.9785	1.5834	-0.5338
H	-7.7281	1.694	-1.3234
H	-7.5074	1.5998	0.4247
H	-6.5158	0.5981	-0.6536
C	-6.6935	4.0667	-0.4865
H	-6.0189	4.921	-0.5975
H	-7.1695	4.1422	0.4964
H	-7.4742	4.1574	-1.2496
C	-3.2275	-2.4182	-3.8993
H	-3.9226	-2.252	-4.7295
H	-2.2264	-2.5433	-4.3215
H	-3.2205	-1.5164	-3.2776
C	-5.298	2.6531	-2.0407
H	-4.5816	3.4644	-2.2003
H	-6.0661	2.7322	-2.8177
H	-4.7695	1.7049	-2.1832
H	-4.6857	0.7864	4.2844

C	-2.3785	7.6311	-2.5125
C	-2.341	8.9038	-1.6329
C	-1.8338	7.9934	-3.9071
C	-3.8443	7.1638	-2.6746
H	-4.4396	7.945	-3.1587
H	-3.9043	6.2621	-3.2932
H	-4.3133	6.9436	-1.7109
H	-2.9263	9.7036	-2.0987
H	-2.7578	8.7248	-0.6374
H	-1.3151	9.2643	-1.5069
H	-2.4518	8.7805	-4.349
H	-0.8074	8.3714	-3.8605
H	-1.8553	7.1378	-4.5904
H	2.4204	-4.512	-4.3282
C	-1.344	3.1087	5.7797
C	0.0804	-6.1948	1.4817
H	-0.965	-6.4981	1.6157
H	0.4998	-6.8106	0.6723
C	0.8561	-6.4001	2.7745
C	1.8003	4.3182	0.9988
C	2.8921	3.3349	1.3891
H	2.2197	5.1482	0.4181
H	1.3169	4.7424	1.8871
C	-1.6929	4.5944	1.4895
H	-2.0243	5.4794	2.049
H	-0.8713	4.1659	2.0641
C	1.5785	4.3969	-2.4604
H	1.936	5.1365	-3.1875
H	2.3896	4.2569	-1.7436
C	2.3568	-6.1008	2.6707
H	0.407	-5.7771	3.5591
H	0.699	-7.4428	3.0765
C	3.1407	-6.3244	3.9744
H	2.7945	-6.7191	1.8734
H	2.4808	-5.0578	2.3577
C	3.2266	-7.7854	4.4374
H	4.1607	-5.9405	3.8385
H	2.6948	-5.7152	4.7741
C	4.0797	-7.9607	5.6975
H	2.2205	-8.1798	4.6281
H	3.6438	-8.3954	3.6245
H	4.1275	-9.0099	6.0049
H	5.1065	-7.6154	5.5323
H	3.67	-7.3875	6.5369
C	1.9691	-4.626	-7.0901
H	0.3428	-4.2472	-5.7046
H	1.0081	-2.7933	-6.4385
C	1.0898	-4.8499	-8.3284
H	2.9119	-4.1467	-7.3834
H	2.2416	-5.6007	-6.6611
C	1.7182	-5.7519	-9.4029
H	0.1368	-5.2917	-8.0077
H	0.8391	-3.8767	-8.7742
C	2.9647	-5.1672	-10.0768
H	1.9642	-6.7255	-8.9576
H	0.9613	-5.9545	-10.1704
H	3.3287	-5.8295	-10.8684
H	2.7472	-4.1946	-10.5332
H	3.7876	-5.0249	-9.3686
C	0.0758	3.5928	5.4625
H	-1.716	3.6202	6.6773
H	-1.3278	2.0371	6.0174
C	1.0553	3.3752	6.6222
H	0.4413	3.0723	4.5683
H	0.0444	4.6624	5.2088
C	2.4815	3.8517	6.3222
H	0.682	3.8949	7.5158
H	1.0785	2.3071	6.8827
C	3.4508	3.6327	7.4876
H	2.8559	3.3275	5.4313
H	2.4581	4.9187	6.0611
H	4.4585	3.9829	7.2424
H	3.1216	4.1719	8.3825
H	3.5221	2.5712	7.7501
C	3.9786	4.0157	2.2361
H	2.4508	2.5044	1.9548
H	3.3372	2.914	0.4775
C	5.1113	3.0706	2.6541
H	4.4013	4.8597	1.6739
H	3.5188	4.4474	3.1353
C	6.1864	3.7528	3.5094
H	4.6906	2.2208	3.2104
H	5.5798	2.644	1.7558
C	7.3182	2.8076	3.9223
H	6.6033	4.6039	2.9545
H	5.7171	4.1769	4.4072
H	8.0662	3.3243	4.5311
H	6.9375	1.9645	4.5099
H	7.8313	2.3952	3.046
C	4.4957	2.9895	-3.3128
C	5.6984	3.3803	-2.4691
H	4.7984	2.3562	-4.1585
H	4.0148	3.8842	-3.7257
C	6.569	2.2121	-1.9925
H	6.3074	4.0585	-3.0819
H	5.3529	3.9707	-1.6103
C	7.8235	2.673	-1.2391
H	5.9763	1.5517	-1.3479
H	6.869	1.6091	-2.8614
C	8.7292	1.5217	-0.7851
H	8.4005	3.3537	-1.8808
H	7.5244	3.2667	-0.363

C	9.9805	1.9968	-0.041
H	8.1561	0.8418	-0.1401
H	9.0267	0.93	-1.6612
H	10.6061	1.1535	0.2673
H	10.5925	2.6509	-0.6718
H	9.7167	2.5619	0.8604
C	-1.6701	-1.9496	4.4994
C	-0.2367	-1.5674	4.8325
H	-2.3711	-1.4322	5.1689
H	-1.8261	-3.0286	4.6378
C	0.0889	-1.874	6.3035
H	0.441	-2.1038	4.1586
H	-0.0991	-0.4958	4.6398
C	1.5054	-1.4751	6.7472
H	-0.6382	-1.3502	6.9382
H	-0.0597	-2.9458	6.4966
C	2.6313	-2.3309	6.1526
H	1.6799	-0.418	6.4983
H	1.5588	-1.5391	7.8418
C	4.0204	-1.9182	6.6489
H	2.4508	-3.3845	6.4065
H	2.6063	-2.2739	5.0574
H	4.8043	-2.549	6.2175
H	4.2445	-0.8785	6.3831
H	4.0924	-2.0004	7.739
C	1.1059	-4.6242	-1.6361
H	1.5482	-5.3767	-0.9789
H	1.1146	-5.0395	-2.6462
C	3.0872	-0.3436	-3.7129
H	4.0938	0.0752	-3.6484
H	3.0239	-0.8814	-4.6609
C	-0.3393	0.3193	0.1262
N	0.8106	0.8722	-0.6058
H	0.9166	1.9448	-0.3862
C	-0.3296	-1.1884	-0.0643
C	-1.5843	0.9448	-0.5009
H	1.7433	0.3515	-0.3432
H	0.6987	0.7549	-1.6938
H	-1.1812	-1.6143	0.4649
H	-0.4128	-1.4682	-1.1175
H	0.5741	-1.6437	0.3471
H	-2.4711	0.6053	0.0369
H	-1.5669	2.0346	-0.4528
H	-1.6841	0.64	-1.5482
H	-0.2762	0.5716	1.1847

________________________--

Energy = -4607.429937a.u.

0 imaginary frequency

$6c^+ \subset 2b^{1,2\text{-}Alt}$

C	0.5643	0.6506	0.5572
N	-0.8985	0.6058	0.7062
H	-1.3991	1.2601	-0.0227
C	0.8824	-0.0361	-0.7676
C	1.1863	-0.081	1.742
H	-1.2875	-0.4177	0.6015
H	0.4002	0.4629	-1.6131
H	0.5847	-1.0883	-0.7551
H	-1.2171	0.9583	1.6982
H	2.2715	-0.0073	1.6596
H	0.8771	0.3596	2.6938
H	0.9337	-1.1382	1.7412
H	1.9585	-0.0119	-0.9374
O	-4.2821	0.9504	2.4183
O	-0.2246	-2.3092	4.1167
O	-2.3798	2.7606	-0.7279
O	0.3534	2.0886	-3.8847
O	2.4332	-1.7038	-2.9304
O	3.2473	-2.5731	0.8116
C	-0.9818	-2.5077	2.9747
C	5.365	0.5226	-3.0267
H	5.6233	1.4858	-3.4503
C	-2.2325	-1.8792	2.8633
C	-1.2586	0.9587	4.5188
H	-0.725	0.2554	5.1459
C	4.0298	0.1008	-3.1261
C	-2.9815	-2.0753	1.6902
H	-3.953	-1.6006	1.6342
C	3.7091	-1.2063	-2.7034
C	-0.4471	-3.2634	1.9047
C	1.2716	2.7769	-3.1013
C	-2.4741	4.4901	0.9478
C	-1.2245	-3.4079	0.7552
H	-0.813	-3.9799	-0.0668
C	3.2203	-4.0743	-1.0855
C	-2.6369	2.692	2.8049
C	3.4916	3.0122	-2.2054
H	4.4962	2.6303	-2.1003
C	-2.3886	0.481	3.8374
C	-3.1103	1.3782	3.0264
C	-0.8202	2.2845	4.4131
C	2.6852	-3.6555	0.1496
C	-1.506	3.1149	3.5111
H	-1.1627	4.1321	3.3521
C	2.5861	2.2829	-2.9876
C	-2.1608	5.7978	1.3427
H	-2.5573	6.1458	2.2901
C	4.674	-2.0197	-2.0788
C	3.1433	4.205	-1.5617
C	2.9858	1.0141	-3.7562

H	2.0754	0.4456	-3.9422
C	-2.5132	-2.8612	0.6317
C	-1.993	4.0366	-0.292
C	0.8555	3.9262	-2.399
C	1.5992	-4.3365	0.7288
C	5.97	-1.5104	-1.9416
H	6.7145	-2.1491	-1.4741
C	-1.1897	4.8502	-1.1135
C	1.8125	4.6292	-1.6643
H	1.5008	5.5197	-1.1316
C	-3.3474	-3.1644	-0.6272
C	-0.9388	6.1572	-0.678
H	-0.3692	6.8066	-1.3363
C	1.1199	-5.4971	0.0994
H	0.3219	-6.0417	0.5925
C	6.3614	-0.2611	-2.4405
C	1.6404	-5.967	-1.1072
C	-1.4074	6.6636	0.5427
C	2.6712	-5.2113	-1.6857
H	3.1085	-5.5458	-2.6225
C	0.3336	2.8515	5.264
C	1.1678	-7.2711	-1.7771
C	7.8379	0.1734	-2.3628
C	0.9869	1.7756	6.1522
H	0.274	1.3445	6.8622
H	1.797	2.2242	6.7349
H	1.418	0.9607	5.5613
C	0.3212	2.5385	-5.2558
H	-0.0748	3.5639	-5.2917
H	1.3447	2.5653	-5.6506
C	-0.2371	3.9581	6.1848
H	-0.6726	4.7801	5.6088
H	0.5564	4.3749	6.8141
H	-1.0166	3.5595	6.8417
C	8.7086	-0.8391	-3.1455
H	9.7636	-0.5474	-3.1022
H	8.4111	-0.88	-4.1982
H	8.6266	-1.85	-2.7357
C	-0.3417	-3.3416	5.1192
C	0.6132	-3.0177	6.257
H	-1.3803	-3.3876	5.4762
C	-2.6664	-2.5374	-1.8654
H	-2.6125	-1.4465	-1.7768
H	-3.2335	-2.7692	-2.7735
H	-1.6477	-2.9117	-2.0031
C	-3.4447	-4.6965	-0.8245
H	-3.908	-5.1753	0.0439
H	-2.4635	-5.1544	-0.9769
H	-4.0565	-4.9248	-1.7037
C	1.4308	3.4616	4.3618
H	2.2219	3.901	4.9784
H	1.0429	4.2535	3.7143
H	1.8902	2.6984	3.7252
C	4.155	5.0477	-0.7597
C	-4.7799	-2.6137	-0.5185
H	-5.3143	-3.0496	0.3317
H	-5.3414	-2.8611	-1.4243
H	-4.7965	-1.5253	-0.4113
C	8.071	1.5703	-2.9685
H	7.5053	2.3449	-2.4405
H	7.7988	1.6071	-4.0283
H	9.1312	1.8308	-2.8941
C	8.2982	0.2059	-0.8869
H	7.7063	0.9212	-0.3061
H	9.3498	0.506	-0.823
H	8.2055	-0.7738	-0.4089
C	2.3534	-8.264	-1.8455
H	2.7271	-8.4978	-0.8435
H	3.1877	-7.8624	-2.428
H	2.0385	-9.201	-2.3174
C	0.0226	-7.947	-0.9998
H	0.3252	-8.2249	0.0149
H	-0.2796	-8.8645	-1.5138
H	-0.8614	-7.3039	-0.9299
C	5.5651	4.4285	-0.7588
H	6.2459	5.0632	-0.1832
H	5.9724	4.3437	-1.7715
H	5.5749	3.4339	-0.3002
C	4.2512	6.4577	-1.3903
H	3.2878	6.9762	-1.3771
H	4.5846	6.3998	-2.4314
H	4.9683	7.0746	-0.838
C	0.6684	-6.9751	-3.2098
H	0.3471	-7.901	-3.6993
H	1.4496	-6.5273	-3.8312
H	-0.1835	-6.2878	-3.1951
C	3.6887	5.1789	0.7096
H	2.6992	5.6393	0.7859
H	4.3893	5.8022	1.2759
H	3.6425	4.199	1.1968
H	3.3611	1.3275	-4.7413
C	-1.1101	8.1223	0.9389
C	-1.73	9.0731	-0.1131
C	-1.694	8.4842	2.3177
C	0.4199	8.3451	0.9913
H	0.643	9.3795	1.2733
H	0.8892	7.6857	1.7291
H	0.8942	8.1596	0.0229
H	-1.5255	10.1159	0.1515
H	-1.3213	8.8989	-1.1128
H	-2.8157	8.9441	-0.1678
H	-1.452	9.5238	2.5571

H	-2.7845	8.3896	2.3385
H	-1.2785	7.8591	3.1152
H	-0.1037	-4.3178	4.6773
C	-0.5395	1.5998	-6.0861
C	4.29	-2.9392	1.7399
H	3.9369	-3.7635	2.3736
H	5.1669	-3.3021	1.1856
C	4.6585	-1.7345	2.5943
C	-3.5884	2.8182	-1.5356
C	-4.2577	1.4581	-1.6282
H	-4.2701	3.5431	-1.0758
H	-3.3285	3.1939	-2.5327
C	-0.6131	4.3672	-2.4403
H	-0.7221	5.1794	-3.1708
H	-1.1928	3.5263	-2.8203
C	-3.3613	3.627	1.8351
H	-4.0053	4.3004	2.4146
H	-4.0265	3.0182	1.2211
C	5.2684	-0.5688	1.8046
H	3.762	-1.3977	3.1319
H	5.3628	-2.0855	3.3585
C	5.5252	0.6955	2.6397
H	6.2112	-0.8995	1.3488
H	4.6054	-0.3236	0.9675
C	6.5802	0.5412	3.7439
H	5.8411	1.5026	1.9643
H	4.5796	1.0337	3.0901
C	6.8537	1.8475	4.4954
H	6.2624	-0.2261	4.4613
H	7.514	0.1726	3.298
H	7.6097	1.7103	5.2747
H	7.2148	2.6277	3.8158
H	5.9449	2.2254	4.9779
C	0.5635	-4.0603	7.3819
H	1.6306	-2.9471	5.8526
H	0.3659	-2.0257	6.6563
C	1.5251	-3.7378	8.5344
H	-0.4635	-4.1328	7.7621
H	0.8096	-5.0511	6.9744
C	1.5492	-4.7866	9.658
H	2.5393	-3.6312	8.1265
H	1.2637	-2.7582	8.9594
C	0.2391	-4.9081	10.4445
H	1.8145	-5.7643	9.2333
H	2.3584	-4.5305	10.3529
H	0.3397	-5.6265	11.2639
H	-0.0492	-3.9454	10.8823
H	-0.5897	-5.2478	9.8148
C	-2.0236	1.5751	-5.7026
H	-0.4387	1.914	-7.1337
H	-0.1201	0.5875	-6.0248
C	-2.8645	0.7075	-6.6473
H	-2.1248	1.2105	-4.6722
H	-2.4154	2.6027	-5.7092
C	-4.3537	0.6604	-6.2874
H	-2.7539	1.0816	-7.6749
H	-2.4629	-0.3159	-6.6555
C	-5.1803	-0.2024	-7.2458
H	-4.4648	0.2783	-5.263
H	-4.7553	1.6831	-6.2772
H	-6.2375	-0.2163	-6.9626
H	-5.1172	0.175	-8.2723
H	-4.8243	-1.2388	-7.2537
C	-5.5736	1.5645	-2.4177
H	-3.583	0.7456	-2.1201
H	-4.4499	1.0828	-0.6148
C	-6.3432	0.2447	-2.54
H	-6.2204	2.3119	-1.9374
H	-5.3595	1.9499	-3.4235
C	-7.6437	0.3761	-3.3429
H	-5.7007	-0.5123	-3.0112
H	-6.5754	-0.1367	-1.5359
C	-8.4191	-0.9397	-3.4539
H	-8.2811	1.139	-2.8761
H	-7.4103	0.7504	-4.3486
H	-9.3394	-0.8123	-4.0318
H	-7.821	-1.7131	-3.9489
H	-8.699	-1.3204	-2.4649
C	-5.4732	1.3889	3.1211
C	-6.7173	0.889	2.4033
H	-5.436	1.0109	4.1522
H	-5.4759	2.4845	3.1693
C	-6.9695	-0.6194	2.5114
H	-7.5714	1.4282	2.8342
H	-6.6636	1.1931	1.3498
C	-8.2718	-1.0598	1.8312
H	-6.1274	-1.168	2.0728
H	-7.0019	-0.9015	3.5734
C	-8.5552	-2.5614	1.961
H	-9.1128	-0.4952	2.2579
H	-8.2358	-0.7882	0.7661
C	-9.8553	-2.9909	1.2753
H	-7.7138	-3.1272	1.5376
H	-8.5957	-2.8301	3.0251
H	-10.0295	-4.0654	1.3871
H	-10.7192	-2.4685	1.7008
H	-9.8309	-2.7682	0.2024
C	2.2935	-2.3507	-4.2086
C	0.8236	-2.6458	-4.4674
H	2.6962	-1.6962	-4.9943
H	2.8825	-3.2785	-4.2207
C	0.6097	-3.2651	-5.8579

H	0.4536	-3.311	-3.6792
H	0.259	-1.7076	-4.3859
C	-0.8613	-3.4525	-6.2627
H	1.0949	-2.6232	-6.6055
H	1.126	-4.2337	-5.9122
C	-1.6286	-4.5135	-5.4635
H	-1.3833	-2.4881	-6.1789
H	-0.898	-3.7226	-7.3262
C	-3.076	-4.6829	-5.9357
H	-1.1027	-5.474	-5.5463
H	-1.6239	-4.2541	-4.3972
H	-3.6006	-5.4492	-5.3561
H	-3.6385	-3.7468	-5.8366
H	-3.1169	-4.9791	-6.9896
C	-2.7906	-0.9852	3.9703
H	-3.88	-1.0669	3.9587
H	-2.4459	-1.346	4.9413
C	4.4813	-3.4901	-1.7107
H	4.6636	-4.0688	-2.6253
H	5.3209	-3.751	-1.055
C	0.9502	-3.8853	2.0276
H	0.8753	-4.7574	2.691
H	1.5922	-3.1719	2.5473
H	0.9068	1.6851	0.5343

______________________--

Energy = -4607.424476 a.u.

0 imaginary frequency

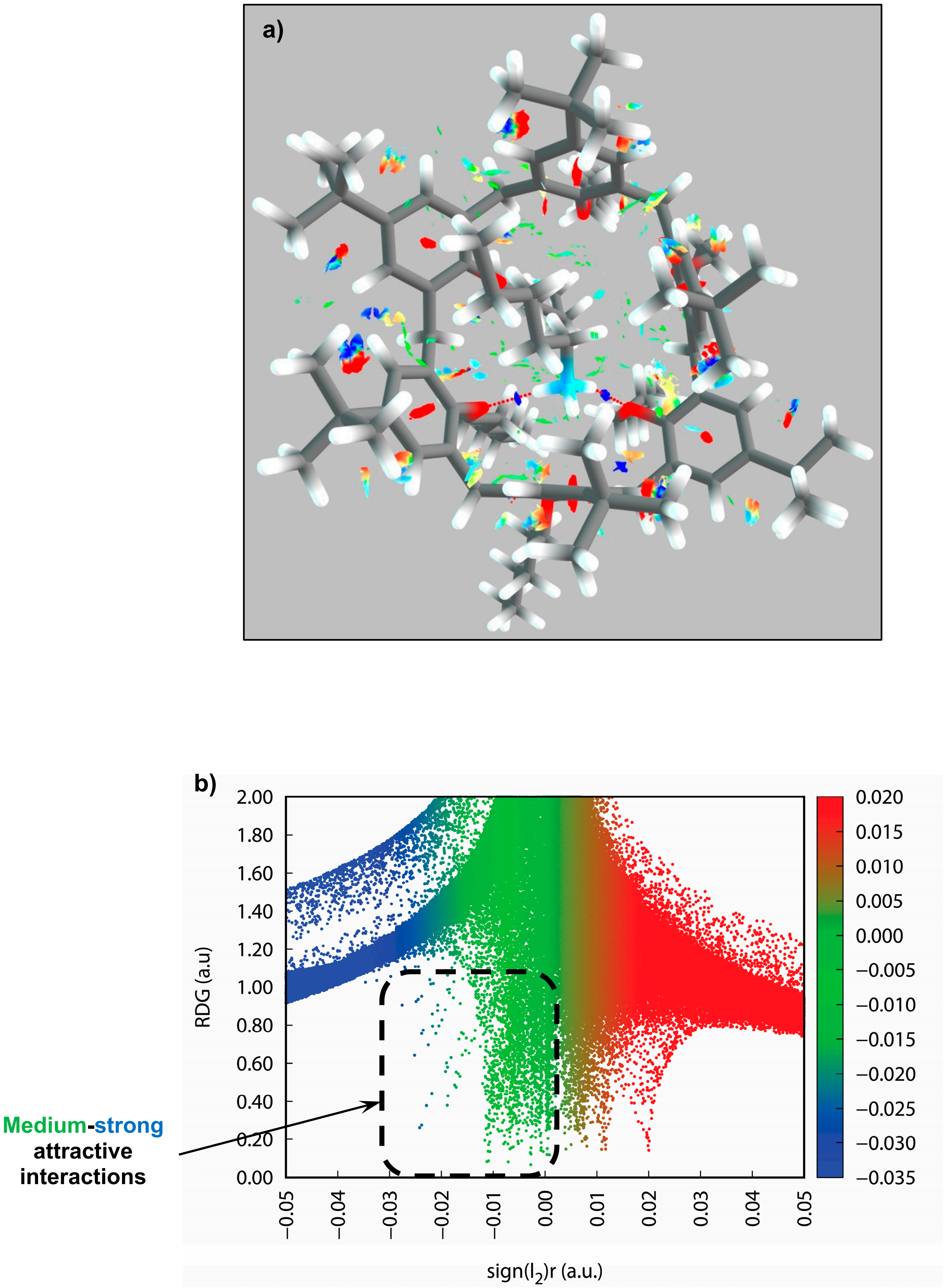

Figure S19. (a) Gradient RDG isosurfaces (0.5) for the noncovalent interaction (NCI) regions in $\mathbf{6a^{+}\subset 2b}^{cone}$ complex. (b) Plot of RDG versus sign(I_2)r for $\mathbf{6a^{+}\subset 2b}^{cone}$complex (NCI-RDG isosurfaces with S = 0.5).

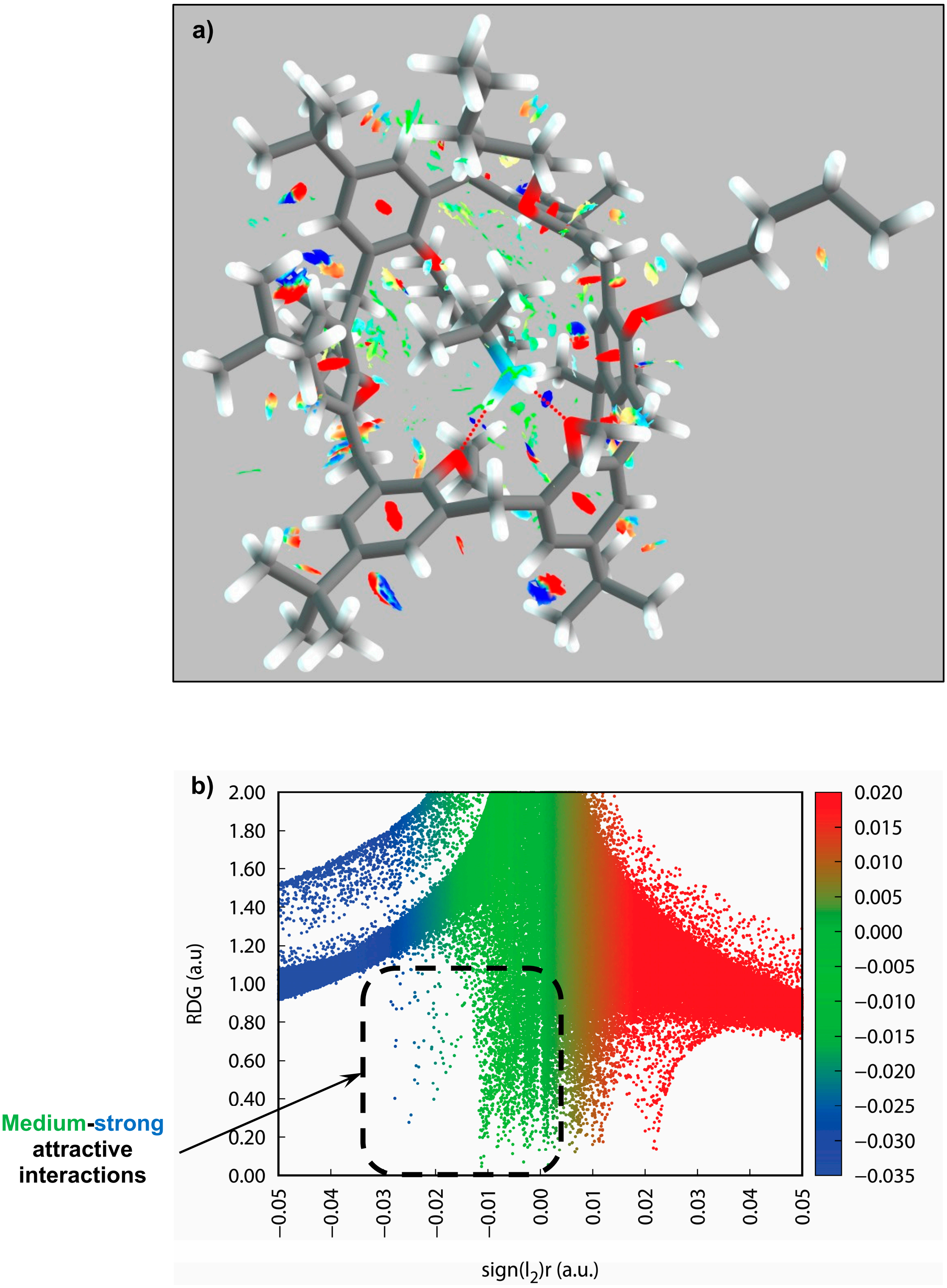

Figure S20. (a) Gradient RDG isosurfaces (0.5) for the noncovalent interaction (NCI) regions in $\mathbf{6b}^{+}\subset\mathbf{2b}^{cone}$ complex (one alkyl chain is omitted for clarity). (b) Plot of RDG versus sign(I_2)r for $\mathbf{6b}^{+}\subset\mathbf{2b}^{1,2,3\text{-}alt}$ complex (NCI-RDG isosurfaces with S = 0.5).

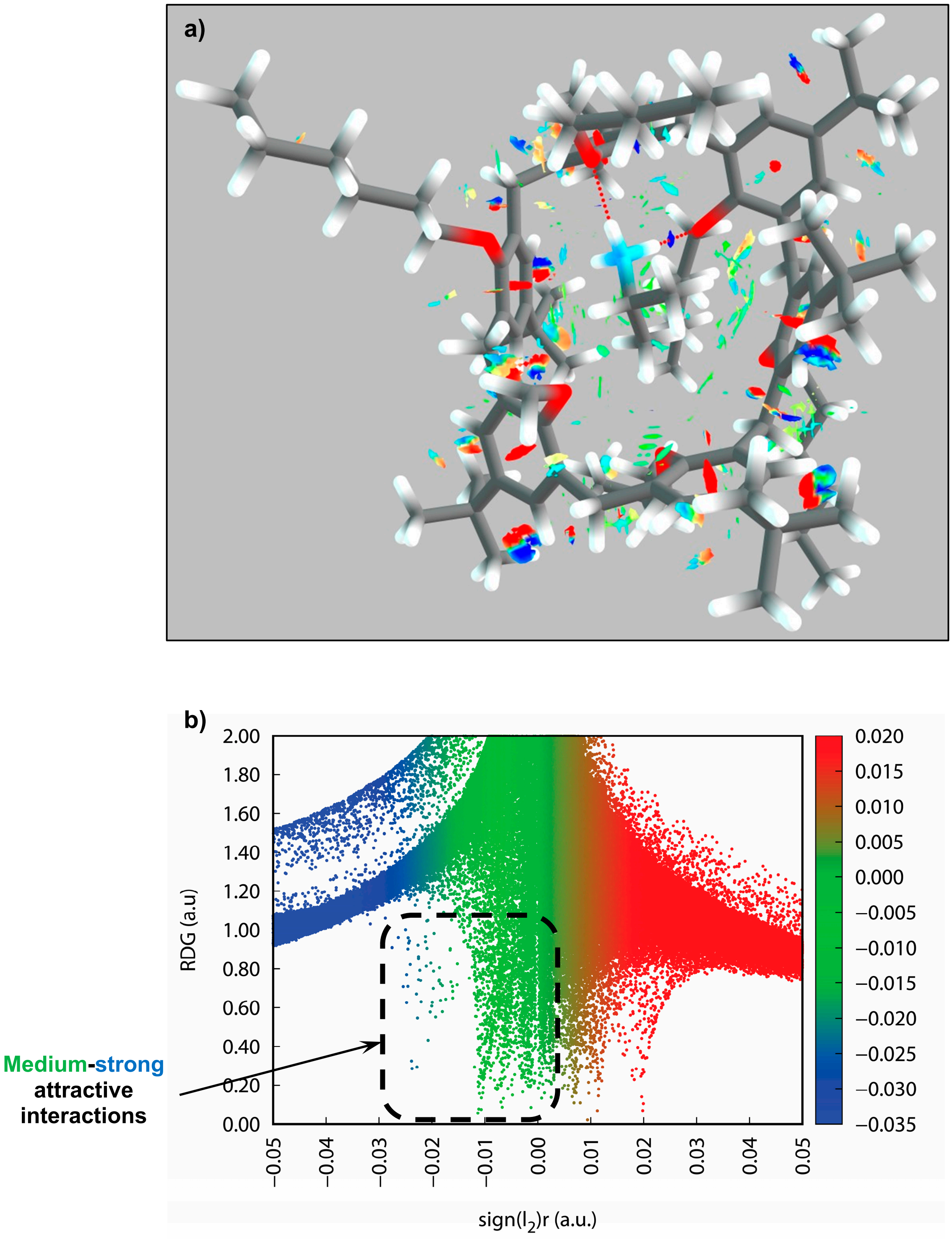

Figure S21. (a) Gradient RDG isosurfaces (0.5) for the noncovalent interaction (NCI) regions in **6c**$^+$⊂**2b**$^{1,2,3\text{-}alt}$ complex (one alkyl chain is omitted for clarity). (b) Plot of RDG versus sign(I_2)r for **6c**$^+$⊂**2b**$^{1,2,3\text{-}alt}$ complex (NCI-RDG isosurfaces with S = 0.5).

References

[1] Gaeta, C.; Troisi, F.; Neri, P. *endo*-Cavity Complexation and Through-the-Annulus Threading of Large Calixarenes Induced by Very Loose Alkylammonium Ion Pairs *Org. Lett.* **2010**, *12*, 2092.
[2] Frisch, M.J.; Trucks, G.W.; Schlegel, H.B.; Scuseria, G.E.; Robb, M.A.; Cheeseman, J.R.; Scalmani, G.; Barone, V.; Mennucci, B.; Petersson, G.A.; et al. Gaussian 16, revision A.03. Gaussian Inc.: Wallingford, CT, USA, **2016**.
[3] Krieger, E. and Vriend, G. YASARA View-molecular graphics for all devices-from smartphones to workstations. *Bioinformatics*, **2014**, *30*, 2981.
[4] Weinhold, F. and Landis, C. R. Valency and Bonding: A Natural Bond Orbital Donor-Acceptor Perspective, Cambridge University Press, 1st edn, **2005**.
[5] Johnson, E. R.; Keinan, S.; Mori-Sánchez, P.; Contreras-García, J.; Cohen, A. J.; Yang, W. Revealing Noncovalent Interactions. *J. Am. Chem. Soc.* **2010**, *132*, 6498.
[6] Lu, T. and Chen, F. W. Multiwfn: A multifunctional wavefunction analyzer *J. Comput. Chem.* **2012**, *33*, 580.
[7] Talotta, C.; Concilio, C., De Rosa, M., Soriente, A., Gaeta, C., Rescifina, A., Ballester, P., Placido, N. Expanding Coefficient: A Parameter To Assess the Stability of Induced-Fit Complexes. *Org. Lett.* **2021**, *23*, 1804.
[8] Jurcik, A.; Bednar, D.; Byska, J.; Marques, S. M.; Furmanova, K.; Daniel, L.; Kokkonen, P.; Brezovsky, J.; Strnad, O.; Stourac, J.; Pavelka, A.; Manak, M.; Damborsky, J.; Kozlikova, B. CAVER Analyst 2.0: Analysis and Visualization of Channels and Tunnels in Protein Structures and Molecular Dynamics Trajectories. Bioinform. **2018**, *34*, 3586.
